# Stereoselective Iridium-N,P-Catalyzed Double Hydrogenation of Conjugated Enones to Saturated Alcohols

**DOI:** 10.1021/jacs.2c02422

**Published:** 2022-05-05

**Authors:** Bram B. C. Peters, Jia Zheng, Suppachai Krajangsri, Pher G. Andersson

**Affiliations:** † Department of Organic Chemistry, Stockholm University, Svante Arrhenius väg 16C, SE-10691Stockholm, Sweden; ‡ School of Chemistry and Physics, University of Kwazulu-Natal, Private Bag X54001, 4000Durban, South Africa

## Abstract

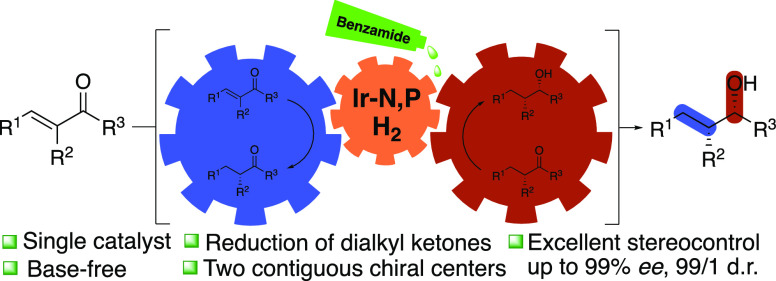

Asymmetric hydrogenation of prochiral substrates such as ketones and olefins constitutes an important instrument for the construction of stereogenic centers, and a multitude of catalytic systems have been developed for this purpose. However, due to the different nature of the π-system, the hydrogenation of olefins and ketones is normally catalyzed by different metal complexes. Herein, a study on the effect of additives on the Ir-N,P-catalyzed hydrogenation of enones is described. The combination of benzamide and the development of a reactive catalyst unlocked a novel reactivity mode of Crabtree-type complexes toward C=O bond hydrogenation. The role of benzamide is suggested to extend the lifetime of the dihydridic iridium intermediate, which is prone to undergo irreversible trimerization, deactivating the catalyst. This unique reactivity is then coupled with C=C bond hydrogenation for the facile installation of two contiguous stereogenic centers in high yield and stereoselectivity (up to 99% *ee*, 99/1 d.r.) resulting in a highly stereoselective reduction of enones.

## Introduction

In the past few decades, there has been an expansion in the number of reported catalytic asymmetric reactions, providing access to various elegant transformations. However, the majority of the established methodologies are focused on the transformation of only a single functional group. Therefore, the development of catalytic reactions that can stereoselectively transform more than one functional group at once is of high interest to improve synthesis efficiency.

**Scheme 1:**
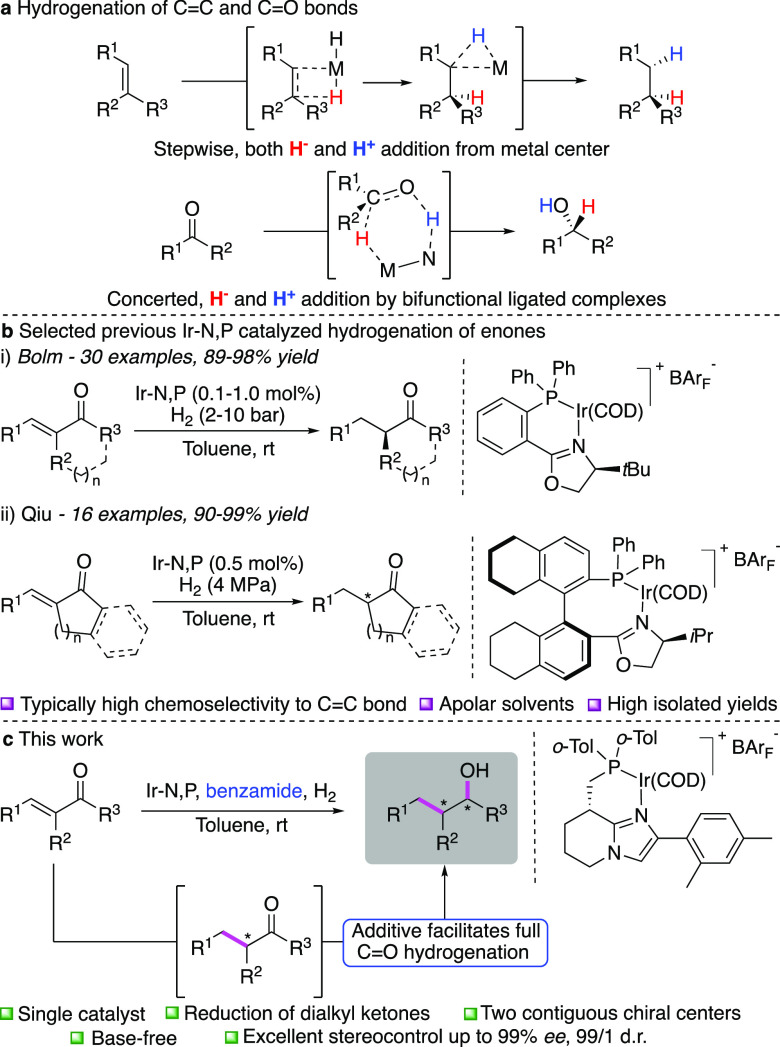
Hydrogenation of Enones (a) Typical reduction of olefins and ketones by hydrogen. (b) Reported asymmetric hydrogenation of enones by Crabtree-type catalysts. (c) This work.

The asymmetric reduction of olefins to chiral alkanes and the reduction of ketones to chiral alcohols are perhaps two of the most well studied areas of stereoselective synthesis.^1^ Normally, the hydrogenation of a C=C or C=O π-bond is catalyzed by different metal complexes since olefins and carbonyls are hydrogenated *via* different mechanisms (Scheme 1a). The addition of hydrogen to an olefin usually only involves a stepwise inner-sphere mechanism.^2^ Ketones are most frequently reduced by an outer-sphere mechanism using bifunctional metal catalysts that operate under basic conditions and reduce the C=O bond *via* a concerted addition of a hydride and a proton.^3^ In these two separate systems, catalysts that perform well in olefin hydrogenation are usually not efficient for carbonyl reduction and vice versa. As an example, nearly perfect chemoselectivity is observed when enones are reduced using Crabtree-type iridium catalysts and molecular hydrogen and give chiral ketones in high yields.^4^ This is demonstrated by contributions from Bolm^4a^ and Qiu^4b^ where Ir-N,P catalysts were used to produce chiral ketones starting from enones (Scheme 1b).

Due to the different nature of the π-system in the C=C and C=O double bond, there are few reports of catalytic systems that reduce both functional groups under the same reaction conditions. Among these, the substrates are normally limited to cyclic aryl ketones under base-mediated (transfer) hydrogenation conditions^5a−5h^ or are directing group-assisted.^5i^ To date, no chiral catalyst is reported that is highly stereoselective in the hydrogenation of both olefins and simple dialkyl ketones. As a result, the hydrogenation of such enones to saturated alcohols still requires a two-step approach.^5c,6^

The introduction of an additive into asymmetric reactions can sometimes largely enhance its catalytic performance. Metal-catalyzed asymmetric hydrogenations have in some cases been reported to benefit from the assistance of additives which have been utilized to either activate pre-catalysts, *in situ* formation of new catalysts, facilitate H_2_ uptake, or increase reactivity and/or selectivity.^7^ In the latter case, additives that are involved in the enantio-determining step have been proposed to either take part in the inner sphere as a ligand, such as in the hydrogenation of imines^8^ or the H_2_-mediated C–C coupling chemistry developed by the Krische group,^9^ or in the outer sphere to open the possibility for a six-membered cyclic transition state.^10^ Regardless of its role, the use of different additives leads to a change in stereoselectivity.

By serendipity, we observed a trace amount of saturated alcohol formation in a previous study on the C=C bond hydrogenation of enones by Ir-N,P catalysts.^4k^ During the optimization of the reaction, we found that the use of additives into the catalytic system greatly improved the reactivity toward carbonyl hydrogenation and, once optimized, granted access to a unique reactivity that conceptually differs from the well studied resolution of α-stereogenity by transfer hydrogenation. Herein, we communicate the asymmetric hydrogenation of enones to produce saturated alcohols catalyzed by a single Ir-N,P catalyst (Scheme 1c). Even though the asymmetric hydrogenation of dialkyl ketones was recently investigated,^11^ the combined hydrogenation of both a ketone and an olefin involves another magnitude of complexity. This facile protocol efficiently installs two contiguous stereogenic centers and overcomes previous limitations in the stereoselective reduction of olefins and ketones.

## Results and Discussion

Our study began by the observation that trace amounts (≤5%) of alcohol **3a** were sometimes formed when attempting to hydrogenate the C=C double bond in enone **1a** to produce chiral ketone **2a** using catalyst **A** (Table 1, Entry 1). However, the formation of **3a** was not reproducible and in most cases the reaction terminated after formation of chiral ketone **2a** in 99% *ee*. Analysis of product **3a** revealed that the catalyst was highly stereoselective, also in the carbonyl reduction, and produced (2*R*,3*R*)-**3a** as a single stereoisomer which absolute configuration was assigned by comparison with reported data.^12^ As explained in the introduction, the hydrogenation of C=C and C=O bonds normally requires different metal complexes. Chiral ligands that are highly enantioselective in olefin hydrogenation often suit the reduction of ketones to a significantly less extent. This motivated us to further investigate the hydrogenation of enones to saturated alcohols using a single catalyst, and a major effort to optimize the reaction began. A brief summary of this work is shown in Table 1 (for more details, see the Supporting Information). First, the structure of the ligand was studied. When catalyst **B** was used, the amount of alcohol formation increased to 28% although accompanied with a loss in stereoselectivity to 90% *ee*, 81/19 d.r. (Table 1, entry 2). In this case, **2a** was also formed with a lower optical purity of 91% *ee*. We rationalized that the change in phosphine substituents could account for the increased reactivity and the imidazole substituent for the selectivity. Therefore, catalyst **C** was developed and gratifyingly hydrogenated **1a** to produce similar amounts of **3a** as **B** but in addition also resulted in excellent control of stereoselectivity (Table 1, Entry 3, 30% **3a**, 99% *ee*, 99/1 d.r.).

**Table 1:**
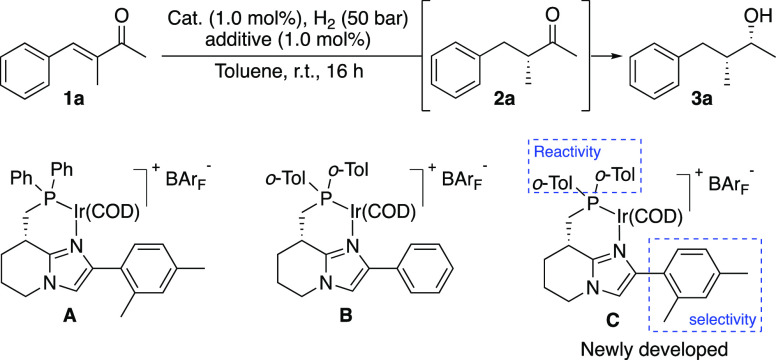

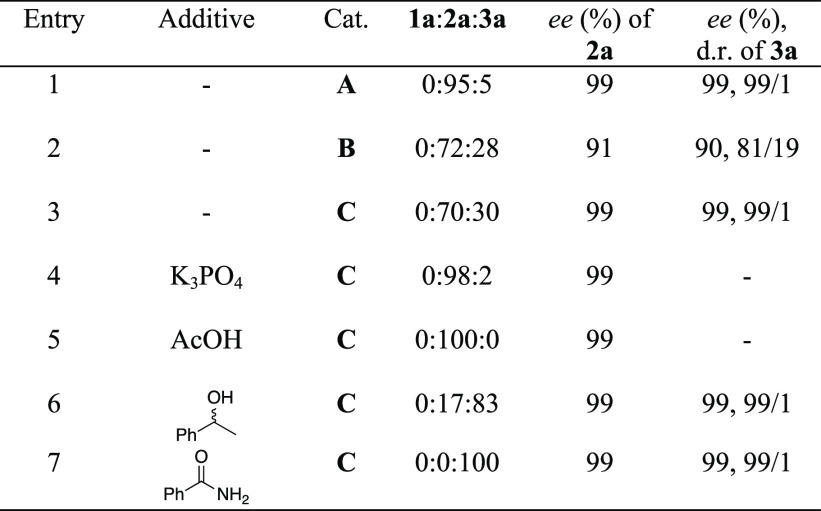
Optimization^a^

a Reaction conditions: 0.05 mmol of the substrate, 1.0 mol % catalyst, 1.0 mol % additive, 1 mL of toluene, 50 bar H_2_, 16 h, rt. Product distribution was determined by ^1^H NMR spectroscopy. Stereoselectivity was determined by GC analysis, using the Chiraldex β-DM stationary phase.

However, the amount of double hydrogenation was still varying from one experiment to another, and we started to investigate the impact of several kinds of additives on the hydrogenation. Addition of an acid or base to the reaction, which is common for the hydrogenation of ketones, had no effect on the hydrogenation of the olefin but instead completely inhibited ketone reduction (Table 1, entries 4–5). Extensive attempts to further promote the reduction of the ketone by different additives were unsuccessful. Finally, we wanted to see if the hydrogenation might be either accelerated or retarded by the product alcohol. Therefore, *rac*-1-phenylethanol was added to the reaction, and it surprisingly increased the conversion of **3a** significantly to 83% (Table 1, entry 6). Further evaluation of numerous additives revealed that benzamide, in an equimolar ratio to the catalyst loading, was optimal to consistently convert **1a** quantitatively to **3a** in 99% *ee* and 99/1 d.r. (Table 1, entry 7).^13^

**Figure 1:**
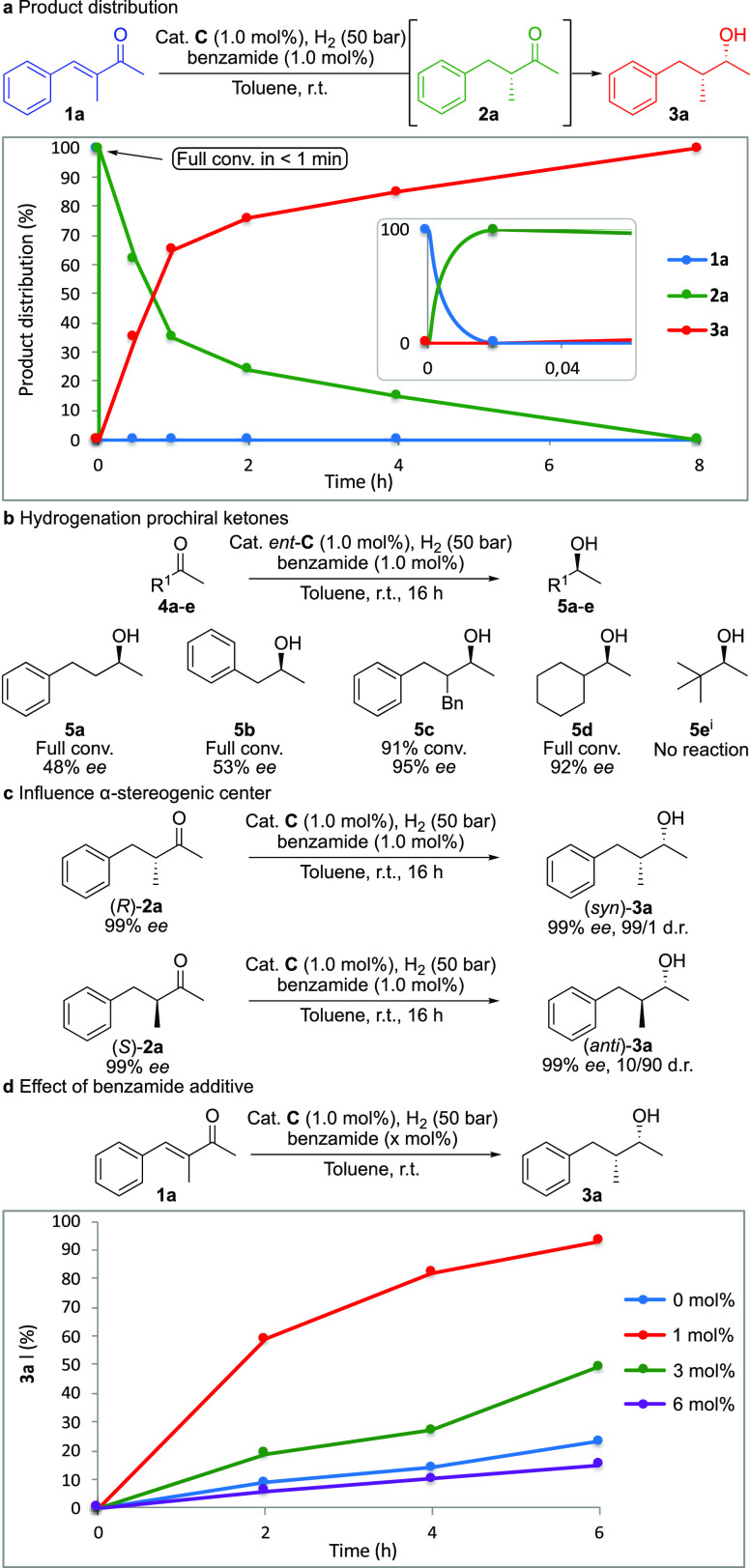
Kinetic plots and control experiments. (a) Product distribution over time. (b) Hydrogenation of prochiral ketones. (c) Influence of the preformed α-stereogenic center. (d) Effect of the benzamide additive. (i) DCM was used.

With the optimized conditions in hand, we continued to investigate the hydrogenation. Analysis of the reaction mixture over time clearly showed that the hydrogenation involves two separate processes (Figure 1a). First, a fast hydrogenation of the olefin (full conversion of **1a** in <1 min) forms **2a**, which is gradually consumed over the course of 8 h to produce fully saturated alcohol **3a**.

The finding that the hydrogenation takes place in two discrete steps prompted us to see whether the catalyst could also hydrogenate an ordinary ketone. Various prochiral ketones (**4a**–**e**) were evaluated (Figure 1b). Ketones bearing two linear alkyl substituents were hydrogenated in poor enantioselectivity (**4a**–**b**). Intriguingly, the enantioselectivity increased tremendously when a branch was introduced in the α-position, and **4c**–**d** were hydrogenated in 95% and 92% *ee*, respectively. Pinacolone (**4e**) did not undergo any reaction, most likely due to steric hindrance. Finally, to study the asymmetric induction of the catalyst *versus* the stereogenic α-carbon, intermediate **2a** was synthesized in both optically pure forms and was individually subjected to the hydrogenation (Figure 1c). When (*R*)-**2a** was evaluated, (2*R*,3*R*)-**3a** was formed in the same stereoselectivity (99% *ee*, 99/1 d.r.) as the reaction proceeds starting from enone **1a**. The pre-installed chirality in the α-position was retained when (*S*)-**2a** was subjected to hydrogenation, forming (2*R*,3*S*)-**3a** in 10/90 d.r. This suggests that the ketone hydrogenation does not progress *via* an enol(ate) intermediate. Moreover, the absolute orientation of the α-chiral center only has a minor influence on the hydrogenation of the ketone (99/1 to 10/90 d.r.), and in both cases, the ketone is preferentially reduced from the same diastereotopic face and in high stereoselectivity. Advantageously, the reduction of both the olefin and the carbonyl proceeds in a stereochemical “matched” manner to produce **3a** as a single stereoisomer.

Then, the reaction was monitored in the presence and absence of benzamide as an additive (Figure 1d). Complete consumption of **1a** was obtained within 1 min in all cases. In the absence of benzamide, 22% of alcohol **3a** was reached in 6 h after which no further conversion was observed; however, consistent reproduction of the reaction was found difficult (blue line). Moreover, half-reduced **2a** was always the dominant species after 16 h in the absence of benzamide. On the other hand, the presence of benzamide in an equimolar amount to the catalyst consistently led to the formation of **3a** as the single product after 16 h, and 93% was formed within 6 h (red line). The use of larger amounts of the additive had a negative effect on the reaction. When 3 and 6 mol % of benzamide was added, the ketone hydrogenation was retarded (green and purple lines, respectively).

**Figure 2:**
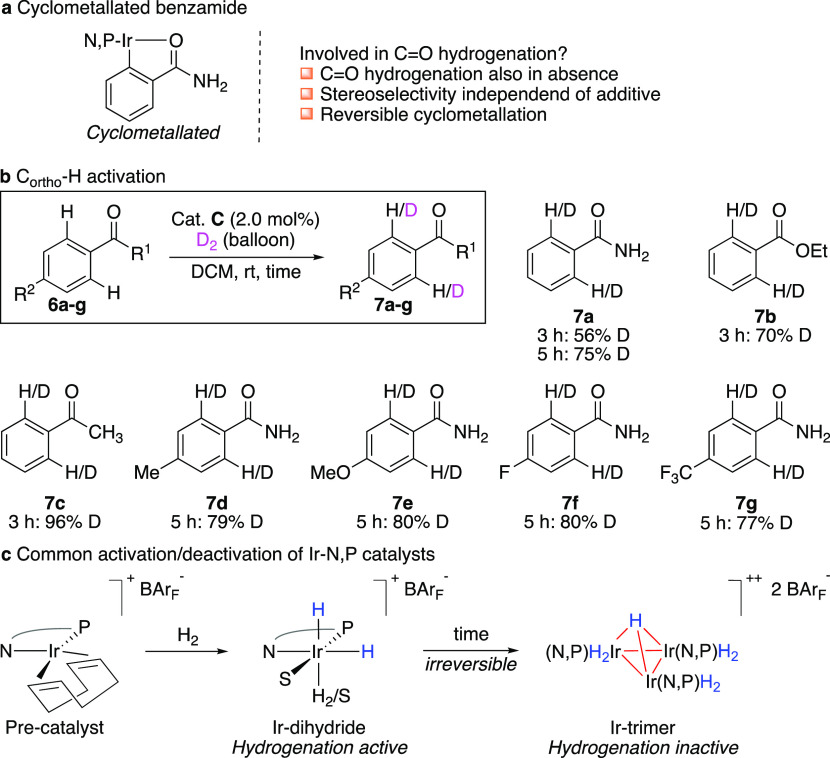
Studies on benzoic acid derivatives. (a) Cyclometallated benzamide. (b) H/D exchange. (c) Trimerization of activated Crabtree-type catalysts.

Iridium complexes have in numerous cases been reported to associate ligands by a cyclometallation reaction. Examples of species that can undergo cyclometallation are imines prior to the hydrogenation of imines,^8^ benzoic acids as observed in C–C bond formations,^9^ and phosphoramidites as described for allylic substitutions.^14^ The cyclometallation is normally an irreversible process, and thus, the properties of the newly formed complex can be fine-tuned to obtain the desired reactivity and selectivity of the catalyst. We hypothesized whether a similar cyclometallated Ir species could be involved in our reaction, accounting for the hydrogenation of the ketone (Figure 2a). Therefore, benzoic acid derivatives **6a–g** were stirred together with catalyst **C** under a D_2_ atmosphere, and it was found that the catalyst can efficiently activate the C_ortho_–H bond in short reaction times (Figure 2b). Interestingly, the least amount of isotope exchange was observed for benzamide **6a** (56% in 3 h, compared to 70 and 96% for **6b** and **6c**, respectively) among these three benzoic acid derivatives. Control experiments using benzamide under a H_2_ atmosphere in the presence of D_2_O or *i*-PrOD (5 equiv.) did not lead to any deuterium incorporation. Substituting the para position of benzamide gave H/D exchange in the same range as benzamide (**7d–g**).

Despite the deuterium experiment is clearly showing that catalyst **C** activates the C_ortho_–H bond in benzamide derivatives, it is unlikely that a cyclometallated Ir species is involved in the ketone hydrogenation since (1) catalyst **C** also can, although to a less extent, hydrogenate the C=O bond in the absence of benzamide, (2) saturated alcohol **3a** is formed in the same stereoselectivities regardless of which additive **6a–c** is used; this would not be expected if catalysts having different ligands **6a–c** would be involved, (3) the reaction was sensitive to the concentration of additives; larger quantities inhibited the hydrogenation, and (4) the addition of benzamide does not promote carbonyl hydrogenation for catalysts that are not able to reduce the C=O bond in the absence of an additive (see the Supporting Information).

Several research groups have investigated the fate of activated iridium catalysts once the hydrogenation is complete. Studies by Crabtree and Pfaltz revealed that these Ir-N,P-dihydride species have the tendency to aggregate and irreversibly form hydrogenation inactive trimers (in the form of [Ir_3_(μ_3_-H)H_6_(N,P)_3_][BAr_F_]_2_) (Figure 2c).^15^ Especially in non-coordinative solvents, that do not particularly stabilize Ir dihydrides, trimerization is a common phenomenon. This event of deactivation takes place either when the starting material is fully consumed or when the hydrogenation rate of a substrate is too slow so that deactivation occurs before complete consumption of the substrate. We propose that the additive in the hydrogenation of the ketone slows down the rate of trimerization by means of reversibly activating the C_ortho_–H bond that consequently minimizes the required concentration of iridium dihydrides to form a trimer. The relatively slow C_ortho_–H bond activation in benzamide most likely assures high concentrations of hydrogenation active species compared to other additives. The presence of benzamide in equimolar quantities to the catalyst accounts for a careful balance between extending the catalyst lifetime and inhibition of the hydrogenation. Larger quantities of benzamide interfered with both C=C and C=O bond hydrogenation (see the Supporting Information).

Lastly, the scope of the hydrogenation was explored (Table 2a). A variety of electron-withdrawing substituents in the para position of the aromatic ring was well tolerated to provide the saturated alcohol in high stereoselectivity (**3b–f**, 99% *ee* ≥98/2 d.r.). Enones bearing electron-donating substituents in all positions of the aromatic ring were also smoothly hydrogenated (**3g–j**). Both naphthyl- and thiophene-substituted enones were found to be compatible (**3k–m**). The α-substituent and the ketone side chain could also be extended, albeit with slightly suppressed yields (**3n–q**). To our delight, the arene ring was exchanged for an alkyl group, and **3r** was produced in excellent stereoselectivity (99% *ee*, 92/8 d.r.).^16^ In addition, the hydrogenation is scalable to at least half a gram scale (Table 2b).

**Table 2:**
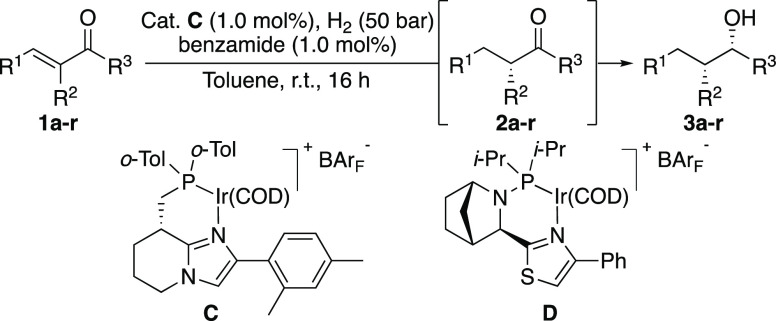

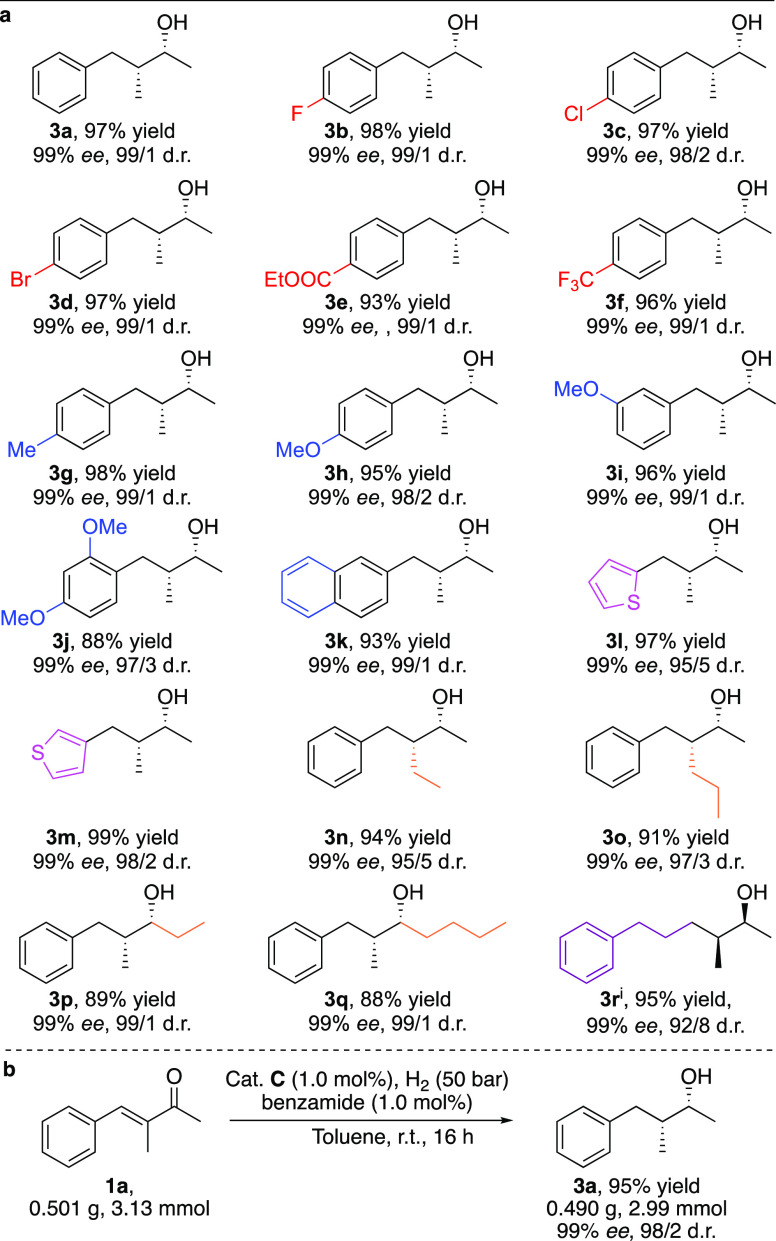
Scope of the Asymmetric Double Hydrogenation of Enones^a^

a Reaction conditions: 0.1 mmol of the substrate, 1.0 mol % catalyst, 1.0 mol % additive, 2 mL of toluene, 50 bar H_2_, 16 h, rt. Product distribution was determined by ^1^H NMR spectroscopy. Stereoselectivity was determined by SFC or GC analysis, using chiral stationary phases. (i) Catalyst **D** was used.

In summary, we have presented the effect of additives on the asymmetric hydrogenation of enones catalyzed by Ir-N,P complexes. By the use of benzamide together with a newly developed reactive catalyst, a novel reactivity toward dialkyl ketones was unlocked. It is suggested that the additive plays a role in the suppression of irreversible deactivation pathways of the catalyst as a result of an efficient C_ortho_–H bond activation. The new reactivity was then coupled with olefin hydrogenation to produce saturated alcohols from enones catalyzed by a single Ir-N,P complex. The presented method allows an atom-economical construction of two contiguous stereogenic centers in excellent control over the formation of possible stereoisomers (up to 99% *ee*, 99/1 d.r.).

## Supporting Information Available

Experimental procedures, optimization of the hydrogenation, control experiments, characterization data of new compounds, separation of chiral products, and NMR spectra of new compounds (PDF)

## References

[ref1] aVerendel J. J.; Pàmies O.; Diéguez M.; Andersson P. G. Asymmetric Hydrogenation of Olefins Using Chiral Crabtree-Type Catalysts: Scope and Limitations. Chem. Rev. 2014, 114, 2130–2169. 10.1021/cr400037u. 24568181

[ref2] aHalpern J. Mechanism and Stereoselectivity of Asymmetric Hydrogenation. Science 1982, 217, 401–407. 10.1126/science.217.4558.401. 17782965

[ref3] aDub P. A.; Gordon J. C. The Role of the Metal-Bound N-H Functionality in Noyori-Type Molecular Catalysts. Nat. Rev. Chem. 2018, 2, 396–408. 10.1038/s41570-018-0049-z.

[ref4] aLu S.-M.; Bolm C. Highly Enantioselective Synthesis of Optically Active Ketones by Iridium-Catalyzed Asymmetric Hydrogenation. Angew. Chem., Int. Ed. 2008, 47, 8920–8923. 10.1002/anie.200803709. 18855959

[ref5] aMolina Betancourt R.; Phansavath P.; Ratovelomanana-Vidal V. Rhodium-Catalyzed Asymmetric Transfer Hydrogenation/Dynamic Kinetic Resolution of 3-Benzylidene-Chromanones. Org. Lett. 2021, 23, 1621–1625. 10.1021/acs.orglett.1c00047. 33600184

[ref6] aLi J.; Lu Y.; Zhu Y.; Nie Y.; Shen J.; Liu Y.; Liu D.; Zhang W. Selective Asymmetric Hydrogenation of Four-Membered Exo-α,β-Unsaturated Cyclobutanones Using RuPHOX-Ru as a Catalyst. Org. Lett. 2019, 21, 4331–4335. 10.1021/acs.orglett.9b01514. 31124691

[ref7] For a detailed review concerning the use of additives in asymmetric catalysis, see: Hong L.; Sun W.; Yang D.; Li G.; Wang R. Additive Effects on Asymmetric Catalysis. Chem. Rev. 2016, 116, 4006–4123. 10.1021/acs.chemrev.5b00676. 26881289

[ref8] aSchramm Y.; Barrios-Landeros F.; Pfaltz A. Discovery of an Iridacycle Catalyst with Improved Reactivity and Enantioselectivity in the Hydrogenation of Dialkyl Ketimines. Chem. Sci. 2013, 4, 2760–2766. 10.1039/c3sc50587a.

[ref9] aKim I. S.; Ngai M.-Y.; Krische M. J. Enantioselective Iridium-Catalyzed Carbonyl Allylation from the Alcohol or Aldehyde Oxidation Level via Transfer Hydrogenative Coupling of Allyl Acetate: Departure from Chirally Modified Allyl Metal Reagents in Carbonyl Addition. J. Am. Chem. Soc. 2008, 130, 14891–14899. 10.1021/ja805722e. 18841896 PMC2890235

[ref10] Ito J.-I.; Teshima T.; Nishiyama H. Enhancement of Enantioselectivity by Alcohol Additives in Asymmetric Hydrogenation with Bis(oxazolinyl)phenyl Ruthenium Catalysts. Chem. Commun. 2012, 48, 1105–1107. 10.1039/c1cc16057e. 22080393

[ref11] Zhang F.-H.; Zhang F.-J.; Li M.-L.; Xie J.-H.; Zhou Q.-L. Enantioselective Hydrogenation of Dialkyl Ketones. Nat. Catal. 2020, 3, 621–627. 10.1038/s41929-020-0474-5.

[ref12] Abate A.; Brenna E.; Fuganti C.; Gatti F. G.; Giovenzana T.; Malpezzi L.; Serra S. Chirality and Fragrance Chemistry: Stereoisomers of the Commercial Chiral Odorants Muguesia and Pamplefleur. J. Org. Chem. 2005, 70, 1281–1290. 10.1021/jo048445j. 15704962

[ref13] Other benzoic acid derivatives such as ethyl benzoate and acetophenone also had a positive effect on the hydrogenation of the ketone compared to no additive, however, complete formation of **3a** was not achieved using these additives.

[ref14] aCheng Q.; Tu H.-F.; Zheng C.; Qu J.-P.; Helmchen G.; You S.-L. Iridium-Catalyzed Asymmetric Allylic Substitution Reactions. Chem. Rev. 2019, 119, 1855–1969. 10.1021/acs.chemrev.8b00506. 30582688

[ref15] aChodosh D. F.; Crabtree R. H.; Felkin H.; Morris G. E. A Tri-Coordinate Hydrogen Ligand in a Trinuclear Iridium Cluster. J. Organomet. Chem. 1978, 161, C67–C70. 10.1016/s0022-328x(00)92254-x.

[ref16] Despite that the *exo*-cyclic olefin in the hydrogenation of enones prepared by a condensation between benzaldehyde and cyclohexanone or cycloheptanone could efficiently be reduced (99% *ee* in both cases), ≤5% of ketone hydrogenation was observed. Also, catalyst **C** is not reactive towards the C=C bond in the hydrogenation of tetrasubstituted enones under optimized reaction conditions and no conversion was observed.

