# Supplementary material for: Stereoselective Iridium-N,P-Catalyzed Double Hydrogenation of Conjugated Enones to Saturated Alcohols
Source: J Am Chem Soc. 2022 May 5;144(19):8734–40. doi: 10.1021/jacs.2c02422 (PMC9121388; doi:10.1021/jacs.2c02422)
Supplement: Supplementary file 1 — ja2c02422_si_001.pdf [file ja2c02422_si_001.pdf]

SUPPORTING INFORMATION

Stereoselective Iridium-N,P Catalyzed Double Hydrogenation of Conjugated Enones to Saturated Alcohols

Bram B. C. Peters,[a] Jia Zheng,[a] Suppachai Krajangsri,[a] and Pher G. Andersson*[ab]

* Pher G. Andersson, E-mail: Pher.Andersson@su.se

[a] Department of Organic Chemistry, Stockholm University, Svante Arrhenius väg 16C, SE-10691 Stockholm, Sweden.

[b] School of Chemistry and Physics, University of KwaZulu-Natal, Private Bag X54001, Durban, 4000, South Africa.

Table of contents

General information……………………………………………………..…………..S-3

Experimental details and characterization data of new compounds…………………………S-4

Synthesis of enones **1a-r**…………………………………………………………..S-4

General procedures for the synthesis of enones……………………………………...S-4

Preparation of catalyst **C**…………………………………………………………..S-5

Optimization of the asymmetric hydrogenation……………………………………………...S-7

General procedure for the asymmetric hydrogenation……………………………………….S-8

Characterization of hydrogenated products…………………………………………S-8

Procedure for the half gram-scale asymmetric hydrogenation……………………………...S-11

Synthesis of (*R*)-**2a** and (*S*)-**2a**………………………………………….......................S-11

General procedure for the H/D exchange……………………..……………………………S-12

Control experiments H/D exchange………………………..…………………..……………S-12

Control experiments using excess of benzamide……………………………….………...S-13

Control experiments using other hydrogenation catalysts…………………………………..S-14

References……………………………………………………………………………………S-15

NMR spectra - Enones……………………………………………………………………...S-16

NMR spectra – Hydrogenated products……………………………………………………….S-17

NMR spectra – Catalyst **C** and intermediates………………………………………………….S-25

Separation of chiral products…………………………………………………………………S-29

Chromatograms………………………………………………………………………………S-31

General information

All reaction vessels were dried in a vacuum oven (160 °C) and cooled down to room temperature under a flow of nitrogen prior to use. Toluene was dried using sodium and freshly distilled under nitrogen. THF was distilled from sodium-benzophenone under nitrogen. The commercially available chemicals were used directly or purified by either distillation or column chromatography. Chromatographic separations were performed on Kiesel gel 60 H silica gel (particle size: 0.063-0.100 mm). Thin-layer chromatography (TLC) was performed on aluminum plates coated with Kiesel gel 60 (0.20 mm, UV 254 nm) and visualized under ultraviolet light followed by staining with potassium permanganate or phosphomolybdic acid. ^{1}H NMR spectra were recorded at 400 MHz in $CDCl_3$ and referenced internally to the residual $CHCl_3$ signal (7.26 ppm). ^{13}C NMR spectra were recorded at 100 MHz in $CDCl_3$ and referenced to the central peak of $CHCl_3$ (77.16 ppm). ^{19}F NMR spectra were recorded at 376 MHz in $CDCl_3$. ^{31}P NMR spectra were recorded at 162 MHz in $CDCl_3$. Chemical shifts were reported in ppm (δ scale), and coupling constants (J) were reported in Hertz (Hz). High resolution mass spectrometric (HRMS) data were obtained from a Bruker microTOF-Q II instrument operated at ambient temperatures. Melting points were determined on a Stuart SMP30 melting point apparatus and reported without correction. Optical rotation was recorded on a thermostated polarimeter using a sodium lamp (589 nm) and a 10 cm cell. Enantiomeric excesses were determined using either GC-MS (30 m column, helium gas carrier at 1.0 mL/min, constant pressure) or SFC-DIAD (250 mm Chiralcel or Chiralpak columns, CO_2/MeOH, 2.0 mL/min) using chiral stationary phases. Racemic compounds were in all cases used for comparison.

Experimental details and characterization data of new compounds

Synthesis of enones 1a-r

Enones **1a**,[1] **1b**,[1] **1c**,[1] **1d**,[2] **1e**,[2] **1f**,[1] **1g**,[3] **1h**,[2] **1i**,[1] **1k**,[1] **1l**,[2] **1m**,[2] **1n**,[1] **1o**,[3] **1p**[2] and **1q**[1] were prepared according to reported procedures and spectroscopic data was in agreement with the values reported therein.

General procedure for the synthesis of enones

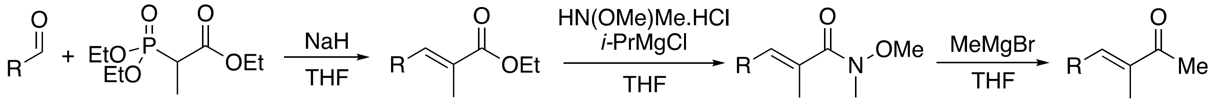

Triethyl-2-phosphonopropionate (2.86 g, 12 mmol, 1.2 equiv.) was added dropwise towards a stirring suspension of NaH (60% in mineral oil, 0.48 g, 12 mmol, 1.2 equiv.) in THF (50 mL) at 0 °C. Stirring was continued for 30 min followed by the addition of suitable aldehyde (10 mmol, 1.0 equiv.). Saturated aqueous NH_4Cl solution (20 mL) was added after 16 h to quench the reactants and the mixture was extracted with Et_2O (3x, 20 mL). The combined organic phases were washed with brine (20 mL), dried over Na_2SO_4 and evaporated *in vacuo* to dryness to give the crude. The crude was purified by column chromatography (pentane/Et_2O, 95:5) to yield the desired α,β-unsaturated ester.

The isolated α,β-unsaturated ester (10 mmol, 1.0 equiv.) was charged in a flask containing HN(OMe)Me.HCl (1.94 g, 20 mmol, 2.0 equiv.) in THF (50 mL) followed by the dropwise addition of ^{i}PrMgCl (3.0M in THF, 13.3 mL, 40 mmol, 4.0 equiv.) at 0 °C over a time period of 30 min. After stirring at room temperature for 1 h, saturated aqueous NH_4Cl solution (20 mL) was added to quench the reactants and the mixture was extracted with Et_2O (3x, 20 mL). The combined organic phases were washed with brine (20 mL), dried over Na_2SO_4 and evaporated *in vacuo* to dryness to give the crude. The crude was purified by column chromatography (pentane/Et_2O, 70:30) to yield the desired α,β-unsaturated Weinreb amide.

The isolated α,β-unsaturated Weinreb amide (10 mmol, 1.0 equiv.) was dissolved in THF (50 mL) followed by the dropwise addition of MeMgBr (1.5M in THF, 7.3 mL, 11 mmol, 1.1 equiv.) at 0 °C. After stirring at room temperature for 1 h, saturated aqueous NH_4Cl solution (20 mL) was added to quench the reactants and the mixture was extracted with Et_2O (3x, 20 mL). The combined organic phases were washed with brine (20 mL), dried over Na_2SO_4 and evaporated *in vacuo* to dryness to give the crude. The crude was purified by column chromatography (pentane/Et_2O, 95:5) to yield the desired α,β-unsaturated ketone.

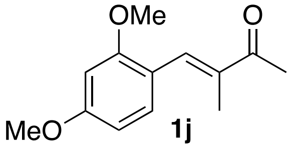

(*E*)-4-(2,4-Dimethoxyphenyl)-3-methylbut-3-en-2-one (1j)

White solid (1.58 g, 72% yield over three steps), prepared according to the general procedure for the synthesis of enones on a 10 mmol scale. **^{1}H NMR** (400 MHz, $CDCl_3$) δ 7.70 (s, 1H), 7.32 (d, *J* = 8.5 Hz, 1H), 6.53 (dd, *J* = 8.5, 2.4 Hz, 1H), 6.48 (d, *J* = 2.4 Hz, 1H), 3.85 (d, *J* = 3.9 Hz, 6H), 2.45 (s, 3H), 2.00 (d, *J* = 1.4 Hz, 3H) ppm. **^{13}C NMR** (100 MHz, $CDCl_3$) δ 200.6, 161.7, 159.0, 136.1, 135.4, 131.3, 117.8, 104.4, 98.4, 55.7, 55.6, 25.9, 13.2 ppm. **HRMS-ESI**: Found $[M+Na]^+$ = 243.0996; $C_{13}H_{16}O_3Na$ requires 243.0992. **m.p.** 82.5 ºC.

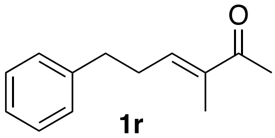

(*E*)-3-Methyl-6-phenylhex-3-en-2-one (1r)

Colorless oil (1.23 g, 67% yield over three steps), prepared according to the general procedure for the synthesis of enones on a 10 mmol scale. Spectroscopic data was in agreement with reported values.[4]

Preparation of catalyst C

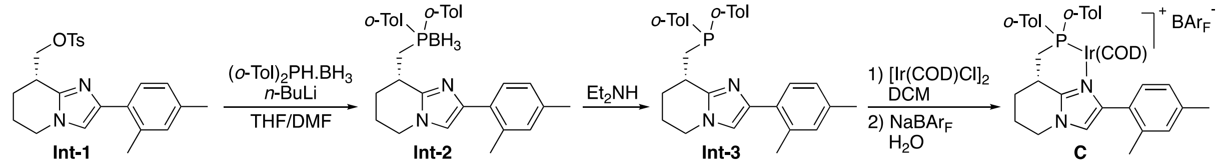

(*S*)-(2-(2,4-Dimethylphenyl)-5,6,7,8-tetrahydroimidazo[1,2-*a*]pyridin-8-yl)methyl 4-methylbenzenesulfonate (**Int-1**) was prepared according to reported procedure[5] and spectroscopic data was in agreement with reported values.[6]

(*S*)-8-((Di-*o*-tolylphosphaneyl)methyl)-2-(2,4-dimethylphenyl)-5,6,7,8-tetrahydroimidazo[1,2-*a*]pyridine borane (**Int-2**) Towards a stirring solution of (*o*-Tol)$_2$PH.BH_3 (0.103 g, 0.65 mmol, 1.5 equiv.) in freshly distilled THF (5 mL) was added dropwise *n*-BuLi (1.5M in hexane, 0.43 mL, 0.65 mmol, 1.5 equiv.) at -78 °C under nitrogen atmosphere. After 10 min, the temperature was increased to 0 °C and stirring was continued for an additional 30 min. Then, tosylate **Int-1** (0.175 g, 0.43 mmol, 1.0 equiv.) in anhydrous DMF (3 mL) was added dropwise and the mixture was stirred over-night after which it was poured into aq. $NaHCO_3$ (10%, 15 mL) and the aqueous phase was extracted with DCM (3x, 15 mL). The combined organic phases were washed with bine (25 mL), dried over Na_2SO_4 and evaporated *in vacuo* to dryness to give the crude. The crude was quickly filtered over a short plug of silica (toluene) to yield **Int-2** (77%, 0.152 g, 0.33 mmol). The product was directly used in further transformations without detailed characterization.

(*S*)-8-((Di-*o*-tolylphosphaneyl)methyl)-2-(2,4-dimethylphenyl)-5,6,7,8-tetrahydroimidazo[1,2-*a*]pyridine (**Int-3**) Borane complex **Int-2** (0.152 g, 0.33 mmol, 1.0 equiv.) was stirred over-night at room temperature in freshly distilled Et_2NH (6 mL) under nitrogen atmosphere. The solvent was evaporated *in vacuo* and the crude was purified by column chromatography (toluene) using deactivated silica to yield **Int-3** (White solid, 70%, 0.106 g, 0.23 mmol). All proceeding steps after evaporation of Et_2NH were always carefully performed under nitrogen atmosphere and degassed solvents were used for the isolation of the product. The product was used in further transformations without detailed characterization. **^{1}H NMR** (400 MHz, $CDCl_3$) δ 7.70 (d, *J* = 7.7 Hz, 1H), 7.68 - 7.63 (m, 1H), 7.25 - 7.23 (m, 2H), 7.20 - 7.11 (m, 4H), 7.10 - 7.00 (m, 3H), 6.84 (s, 1H), 4.04 - 3.90 (m, 2H), 3.25 (ddd, *J* = 14.5, 4.3, 3.0 Hz, 1H), 3.11 - 2.99 (m, 1H), 2.49 (s, 3H), 2.44 (s, 3H), 2.41 (s, 3H), 2.40 - 2.34 (m, 1H), 2.31 (s, 3H), 2.14 - 2.00 (m, 2H), 1.97 - 1.85 (m, 1H), 1.83 - 1.72 (m, 1H) ppm. **^{13}C NMR** (100 MHz, $CDCl_3$) δ 147.9 (d, *J* = 12.2 Hz), 142.7 (d, *J* = 25.2 Hz), 141.8 (d, *J* = 26.1 Hz), 140.2, 137.3 (d, *J* = 11.8 Hz), 136.1, 135.9 (d, *J* = 13.4 Hz), 134.8, 132.0, 131.7, 131.5, 131.3, 130.1 (dd, *J* = 16.2, 4.8 Hz), 128.6 (d, *J* = 3.0 Hz), 128.5, 126.7, 126.5, 126.1, 116.3, 68.1, 45.0, 33.6 (d, *J* = 18.0 Hz), 31.5 (d, *J* = 12.8 Hz), 29.8, 28.2 (d, *J* = 10.8 Hz), 25.8, 22.1, 21.8, 21.5, 21.2 (d, *J* = 5.1 Hz) ppm. **^{31}P NMR** (162 MHz, $CDCl_3$) δ -42.1 ppm. **$[\alpha]_D^{25}$** = + 71 (c = 0.1, $CHCl_3$). **HRMS-ESI**: Found $[M+H]^+$ = 453.2444; $C_{30}H_{34}PN_2$ requires 453.2454.

Catalyst (**C**) Ligand **Int-3** (0.052 g, 0.12 mmol, 1.0 equiv.) and $[Ir(COD)Cl]_2$ (0.040 g, 0.06 mmol, 0.5 equiv.) were stirred in freshly distilled DCM (10 mL) under reflux conditions. After 1 h, the mixture was cooled to room temperature, $NaBAr_F$ (0.125 g, 0.14 mmol, 1.2 equiv.) and H_2O (10 mL) were added and stirring was continued for an additional 30 min. The phases were separated and the aqueous phase was extracted with DCM (2x, 10 mL). The combined organic phases were dried over Na_2SO_4 and evaporated *in vacuo* to dryness to give the crude. The crude was purified by column chromatography (pentane/DCM, 50:50) to yield catalyst **C** (Orange solid, 73%, 130 mg, 0.8 mmol). **^{1}H NMR** (400 MHz, $CDCl_3$) δ 8.78 (ddd, *J* = 16.5, 5.7, 3.4 Hz, 1H), 7.72 (s, 8H), 7.52 (s, 6H), 7.37 - 7.33 (m, 3H), 7.30 (d, *J* = 7.9 Hz, 1H), 7.20 (d, *J* = 7.9 Hz, 1H), 7.11 (s, 2H), 6.82 (s, 1H), 6.70 - 6.62 (m, 1H), 4.75 - 4.67 (m, 1H), 4.04 - 3.81 (m, 3H), 3.56 (p, *J* = 7.4 Hz, 1H), 3.47 - 3.42 (m, 1H), 2.72 (s, 3H), 2.52 - 2.43 (m, 1H), 2.41 (s, 3H), 2.38 - 2.32 (m, 1H), 2.26 (s, 3H), 2.23 - 2.10 (m, 5H), 2.07 (s, 3H), 2.01 - 1.95 (m, 2H), 1.86 - 1.72 (m, 2H), 1.31 - 1.14 (m, 4H) ppm. **^{13}C NMR** (100 MHz, $CDCl_3$) δ 161.8 (dd, *J* = 99.7, 49.9 Hz), 146.8 (d, *J* = 3.5 Hz), 142.3, 142.1 (d, *J* = 28.8 Hz), 140.4, 139.9, 139.8, 136.4, 134.9, 133.3 (d, *J* = 5.3 Hz), 133.1 (d, *J* = 2.4 Hz), 132.8 - 132.1 (m), 131.7, 131.6, 131.1, 129.0 (dddd, *J* = 63.0, 31.6, 5.2, 3.0 Hz), 128.8, 128.1, 126.5 (d, *J* = 10.5 Hz), 126.4, 126.0, 125.9 (d, *J* = 15.9 Hz), 124.5 (d, *J* = 48.6 Hz), 123.3, 120.6, 119.8, 117.7 - 117.5 (m), 95.7 (d, *J* = 9.7 Hz), 91.2 (d, *J* = 14.3 Hz), 62.6 (d, *J* = 40.7 Hz), 46.1, 36.4 (d, *J* = 5.2 Hz), 34.5 - 34.3 (m), 33.4, 28.3 (d, *J* = 15.6 Hz), 27.9, 26.9 (d, *J* = 32.1 Hz), 26.1, 22.9, 22.6 (d, *J* = 5.9 Hz), 21.3, 20.5, 20.4 ppm. **^{31}P NMR** (162 MHz, $CDCl_3$) δ 18.1 ppm. **^{19}F NMR** (377 MHz, $CDCl_3$) δ -62.4 (d, *J* = 2.5 Hz) ppm. **$[\alpha]_D^{27}$** = + 8 (c = 0.1, $CHCl_3$). **HRMS-ESI**: Found $[M-B_{ArF}]^+$ = 753.2442; $C_{38}H_{45}IrPN_2$ requires 753.2446.

Optimization of the asymmetric hydrogenation

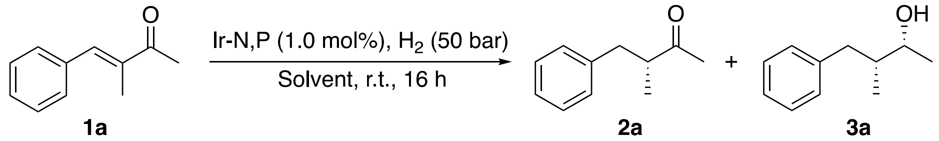

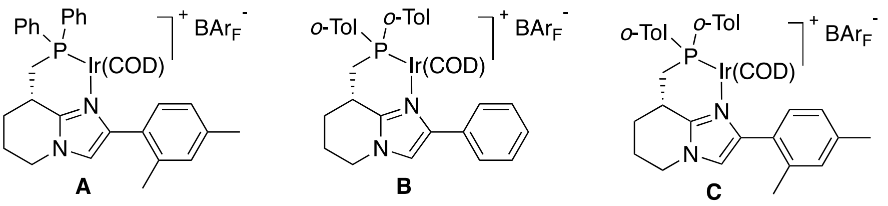

Entry	Solvent	Catalyst	**1a**:**2a**:**3a**	*ee* (%) of **2a**	*ee* (%), d.r. of **3a**
1	Toluene	**A**	0:95:5	99	99, 99/1
2	Toluene	**B**	0:72:28	91	90, 81/19
3	Toluene	**C**	0:70:30	99	99, 99/1
4	DCM	**C**	0:74:26	99	99, 99/1
5	MeOH	**C**	100:0:0	-	-

Reaction conditions: 0.05 mmol of substrate, 1.0 mol% catalyst, 1 mL solvent, 50 bar H_2, 16 h, rt. Product distribution was determined by ^{1}H NMR spectroscopy. Stereoselectivity was determined by GC analysis using Chiraldex β-DM stationary phase.

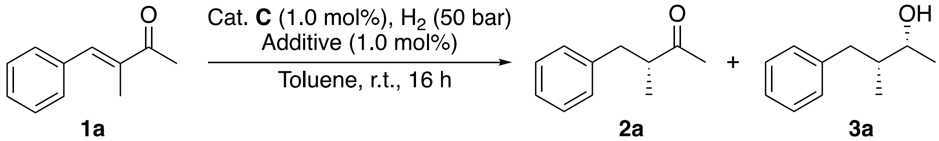

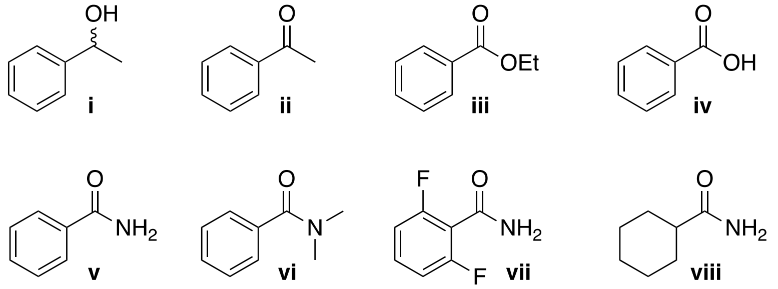

Entry	Additive	**1a**:**2a**:**3a**	*ee* (%) of **2a**	*ee* (%), d.r. of **3a**
1	K_3PO_4	0:98:2	99	-
2	HOAc	0:100:0	99	-
3	**i**	0:17:83	99	99, 99/1
4	**ii**	0:56:44	99	99, 98/2
5	**iii**	0:34:66	99	99, 98/2
6	**iv**	0:100:0	99	-
7	**v**	0:0:100	-	99, 99/1
8	**vi**	0:71:29	99	99, 99/1
9	**vii**	0:50:50	99	99, 99/1
10	**viii**	0:85:15	99	99, 99/1
11[a]	**v**	0:32:68	99	99, 99/1
12[b]	**v**	0:21:79	99	99, 99/1
12[c]	**v**	0:65:35	99	99, 99/1

Reaction conditions: 0.05 mmol of substrate, 1.0 mol% catalyst, 1.0 mol% additive, 1 mL toluene, 50 bar H_2, 16 h, rt. Product distribution was determined by ^{1}H NMR spectroscopy. Stereoselectivity was determined by GC analysis using Chiraldex β-DM stationary phase. [a]2 mol% benzamide was used. [b]0.5 mol% benzamide was used. [c]20 bar H_2.

General procedure for the asymmetric hydrogenation

An oven-dried vial was charged with an enone (0.1 mmol, 1.0 equiv.), Ir-N,P-catalyst (1.0 mol%) and benzamide (1.0 mol%). Distilled toluene (2 mL) and a magnetic stirring bar were added and the vial was placed in a high-pressure hydrogenation apparatus. The reactor was purged three times with Ar, purged three times with H_2 and then pressurized with H_2 (50 bar). The reaction was stirred at room temperature for 16 h before the H_2 pressure was released and the solvent was removed under reduced pressure. The residue was purified by flash chromatography (pentane/Et_2O, 50/50) on silica gel to give the alcohol. The *ee* value was determined by GC analysis or SFC analysis using a chiral stationary phase. The corresponding racemic product was used for comparison. The racemate was prepared by $NaBH_4$ reduction in methanol and subsequent hydrogenation on a 0.1 mmol scale using Pd/C as the catalyst, following the same asymmetric hydrogenation procedure. The absolute configuration was determined by comparison of the optical rotation with reported values.

Characterization of hydrogenated products

Spectroscopic data of alcohols **3a**,[7] **3b**,[7] **3c**,[7] **3d**,[7] **3g**,[7] **3h**,[7] **3i**,[7] **3m**,[7] **3p**,[7] **3q**[7] and **3r**[8] was in agreement with reported values.

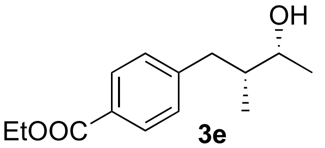

Ethyl 4-((2*R*,3*R*)-3-hydroxy-2-methylbutyl)benzoate (3e)

Colorless oil (21.9 mg, 93% yield), prepared according to the general procedure for the asymmetric hydrogenation on a 0.1 mmol scal e. **^{1}H NMR** (400 MHz, $CDCl_3$) δ 7.96 (dt, *J* = 8.3, 1.8 Hz, 2H), 7.24 (dt, *J* = 8.3, 1.8 Hz, 2H), 4.36 (q, *J* = 7.1 Hz, 2H), 3.79 - 3.69 (m, 1H), 2.89 (dd, *J* = 13.3, 5.8 Hz, 1H), 2.44 (dd, *J* = 13.3, 9.0 Hz, 1H), 1.85 - 1.74 (m, 1H), 1.38 (t, *J* = 7.1 Hz, 3H), 1.20 (d, *J* = 6.4 Hz, 3H), 0.85 (d, *J* = 6.8 Hz, 3H) ppm. **^{13}C NMR** (100 MHz, $CDCl_3$) δ 166.8, 146.9, 129.7, 129.3, 128.4, 70.4, 60.9, 41.8, 39.4, 20.6, 14.5, 13.6 ppm. **$[\alpha]_D^{27}$** = + 10 (c = 0.1, $CHCl_3$). **HRMS-ESI**: Found $[M+Na]^+$ = 259.1309; $C_{14}H_{20}O_3Na$ requires 259.1305.

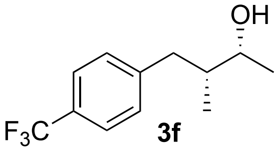

(2*R*,3*R*)-3-Methyl-4-(4-(trifluoromethyl)phenyl)butan-2-ol (3f)

Colorless oil (22.3 mg, 96% yield), prepared according to the general procedure for the asymmetric hydrogenation on a 0.1 mmol scale. **^{1}H NMR** (400 MHz, $CDCl_3$) δ 7.53 (d, *J* = 7.6 Hz, 2H), 7.29 (d, *J* = 7.8 Hz, 2H), 3.80 - 3.72 (m, 1H), 2.90 (dd, *J* = 13.3, 5.8 Hz, 1H), 2.45 (dd, *J* = 13.3, 9.1 Hz, 1H), 1.85 - 1.74 (m, 1H), 1.21 (d, *J* = 6.4 Hz, 3H), 0.86 (d, *J* = 6.8 Hz, 3H) ppm. **^{13}C NMR** (100 MHz, $CDCl_3$) δ 145.7 (q, $^4J_{C\text{-}F}$ = 1.3 Hz), 129.7, 128.5 (q, $^2J_{C\text{-}F}$ = 32.4 Hz), 125.4 (q, $^3J_{C\text{-}F}$ = 3.8 Hz), 123.3 (q, $^1J_{C\text{-}F}$ = 271.9 Hz), 70.4, 41.8, 39.3, 20.7, 13.7 ppm. **^{19}F NMR** (377 MHz, $CDCl_3$) δ -62.3 ppm. **$[\alpha]_D^{26}$** = + 6 (c = 0.1, $CHCl_3$). **HRMS-ESI**: Found $[M+Na]^+$ = 255.0966; $C_{12}H_{15}OF_3Na$ requires 255.0967.

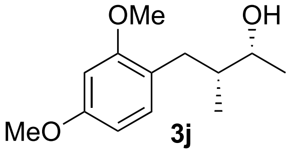

3j **(2*R*,3*R*)-4-(2,4-Dimethoxyphenyl)-3-methylbutan-2-ol (3j)**

Colorless oil (19.7 mg, 88% yield), prepared according to the general procedure for the asymmetric hydrogenation on a 0.1 mmol scale. **^{1}H NMR** (400 MHz, $CDCl_3$) δ 7.02 (d, *J* = 8.6 Hz, 1H), 6.46 - 6.43 (m, 2H), 3.81 (s, 3H), 3.80 (s, 3H), 3.68 – 3.60 (m, 1H), 2.75 (dd, *J* = 13.6, 8.2 Hz, 1H), 2.33 (dd, *J* = 13.6, 7.0 Hz, 1H), 1.72 - 1.65 (m, 1H), 1.13 (d, *J* = 6.5 Hz, 3H), 0.90 (d, *J* = 6.8 Hz, 3H) ppm. **^{13}C NMR** (100 MHz, $CDCl_3$) δ 159.3, 158.4, 131.3, 121.8, 104.5, 98.6, 68.9, 55.6, 55.5, 41.1, 33.1, 20.2, 13.6 ppm. **$[\alpha]_D^{27}$** = + 1 (c = 0.1, $CHCl_3$). **HRMS-ESI**: Found $[M+Na]^+$ = 247.1306; $C_{13}H_{20}O_3Na$ requires 247.1305.

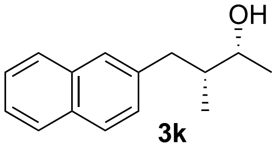

3k **(2*R*,3*R*)-3-Methyl-4-(naphthalen-2-yl)butan-2-ol (3k)**

White solid (19.9 mg, 93% yield), prepared according to the general procedure for the asymmetric hydrogenation on a 0.1 mmol scale. **^{1}H NMR** (400 MHz, $CDCl_3$) δ 7.83 - 7.76 (m, 3H), 7.62 (s, 1H), 7.44 (pd, *J* = 6.9, 1.6 Hz, 2H), 7.34 (dd, *J* = 8.4, 1.8 Hz, 1H), 3.85 - 3.77 (m, 1H), 2.99 (dd, *J* = 13.3, 5.9 Hz, 1H), 2.57 (dd, *J* = 13.4, 8.9 Hz, 1H), 1.96 - 1.84 (m, 1H), 1.23 (d, *J* = 6.4 Hz, 3H), 0.91 (d, *J* = 6.8 Hz, 3H) ppm. **^{13}C NMR** (100 MHz, $CDCl_3$) δ 138.9, 133.7, 132.1, 128.0, 127.9, 127.7, 127.6, 127.5, 126.0, 125.3, 70.5, 41.8, 39.6, 20.7, 13.8 ppm. **$[\alpha]_D^{26}$** = + 4 (c = 0.1, $CHCl_3$). **HRMS-ESI**: Found $[M+Na]^+$ = 237.1250; $C_{15}H_{18}ONa$ requires 237.1250. **m.p.** 47.2 °C.

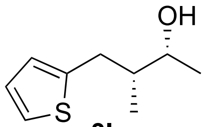

3l **(2*R*,3*R*)-3-Methyl-4-(thiophen-2-yl)butan-2-ol (3l)**

Colorless oil (16.5 mg, 97% yield), prepared according to the general procedure for the asymmetric hydrogenation on a 0.1 mmol scale. **^{1}H NMR** (400 MHz, $CDCl_3$) δ 7.13 (dd, *J* = 5.1, 1.2 Hz, 1H), 6.92 (dd, *J* = 5.1, 3.4 Hz, 1H), 6.81 - 6.79 (m, 1H), 3.85 - 3.77 (m, 1H), 3.01 (dd, *J* = 14.6, 6.1 Hz, 1H), 2.68 (dd, *J* = 14.6, 8.6 Hz, 1H), 1.86 - 1.74 (m, 1H), 1.20 (d, *J* = 6.4 Hz, 3H), 0.93 (d, *J* = 6.8 Hz, 3H) ppm. **^{13}C NMR** (100 MHz, $CDCl_3$) δ 144.0, 126.9, 125.3, 123.4, 70.1, 42.3, 33.6, 20.7, 13.7 ppm. **$[\alpha]_D^{27}$** = + 12 (c = 0.1, $CHCl_3$). **HRMS-ESI**: Found $[M+Na]^+$ = 193.0673; $C_9H_{14}SONa$ requires 193.0658.

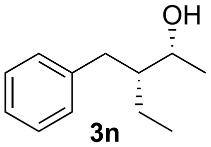

3n **(2*R*,3*R*)-3-Benzylpentan-2-ol (3n)**

Colorless oil (16.7 mg, 94% yield), prepared according to the general procedure for the asymmetric hydrogenation on a 0.1 mmol scale. **^{1}H NMR** (400 MHz, $CDCl_3$) δ 7.31 - 7.25 (m, 2H), 7.22 - 7.16 (m, 3H), 3.91 - 3.82 (m, 1H), 2.76 (dd, J = 13.6, 6.8 Hz, 1H), 2.53 (dd, J = 13.6, 7.7 Hz, 1H), 1.68 - 1.58 (m, 1H), 1.43 - 1.31 (m, 2H), 1.18 (d, J = 6.4 Hz, 3H), 0.92 (t, J = 7.5 Hz, 3H) ppm. **^{13}C NMR** (100 MHz, $CDCl_3$) δ 141.6, 129.3, 128.4, 125.9, 68.6, 48.6, 35.9, 21.7, 20.0, 12.0 ppm. **$[\alpha]_D^{27}$** = - 7 (c = 0.1, $CHCl_3$). **HRMS-ESI**: Found $[M+Na]^+$ = 201.1277; $C_{12}H_{18}ONa$ requires 201.1250.

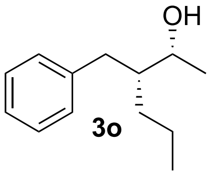

3o **(2*R*,3*R*)-3-Benzylhexan-2-ol (3o)**

Colorless oil (17.5 mg, 91% yield), prepared according to the general procedure for the asymmetric hydrogenation on a 0.1 mmol scale. **^{1}H NMR** (400 MHz, $CDCl_3$) δ 7.32 - 7.24 (m, 2H), 7.23 - 7.14 (m, 3H), 3.84 (s, 1H), 2.75 (dd, J = 13.6, 7.1 Hz, 1H), 2.53 (dd, J = 13.6, 7.3 Hz, 1H), 1.74 - 1.67 (m, 1H), 1.37 - 1.28 (m, 4H), 1.16 (d, J = 6.4 Hz, 3H), 0.87 (t, J = 6.8 Hz, 3H) ppm. **^{13}C NMR** (100 MHz, $CDCl_3$) δ 141.8, 129.4, 128.5, 126.0, 68.9, 46.8, 36.5, 31.4, 20.9, 20.1, 14.6 ppm. **$[\alpha]_D^{27}$** = - 4 (c = 0.1, $CHCl_3$). **HRMS-ESI**: Found $[M+Na]^+$ = 215.1406; $C_{13}H_{20}ONa$ requires 215.1406.

Procedure for the half gram-scale asymmetric hydrogenation

An oven-dried vial was charged with enone **1a** (0.501 g, 3.13 mmol, 1.0 equiv.), Ir-N,P-catalyst **C** (50.6 mg, 1.0 mol%) and benzamide (3.8 mg, 1.0 mol%). Distilled toluene (10 mL) and a magnetic stirring bar were added and the vial was placed in a high-pressure hydrogenation apparatus. The reactor was purged three times with Ar, purged three times with H_2 and then pressurized with H_2 (50 bar). The reaction was stirred at room temperature for 16 h before the H_2 pressure was released and the solvent was removed under reduced pressure. The residue was purified by flash chromatography (pentane/Et_2O, 50/50) on silica gel to give the alcohol **3a** (0.490 g, 2.99 mmol, 95% yield). The *ee* value was determined by GC analysis using Chiraldex β-DM chiral stationary phase. The corresponding racemic product was used for comparison. The racemate was prepared by $LiAlH_4$ reduction in methanol and subsequent hydrogenation on a 0.1 mmol scale using Pd/C as the catalyst, following the same asymmetric hydrogenation procedure. The absolute configuration was determined by comparison of the optical rotation with reported values.

Synthesis of (*R*)-2a and (*S*)-2a

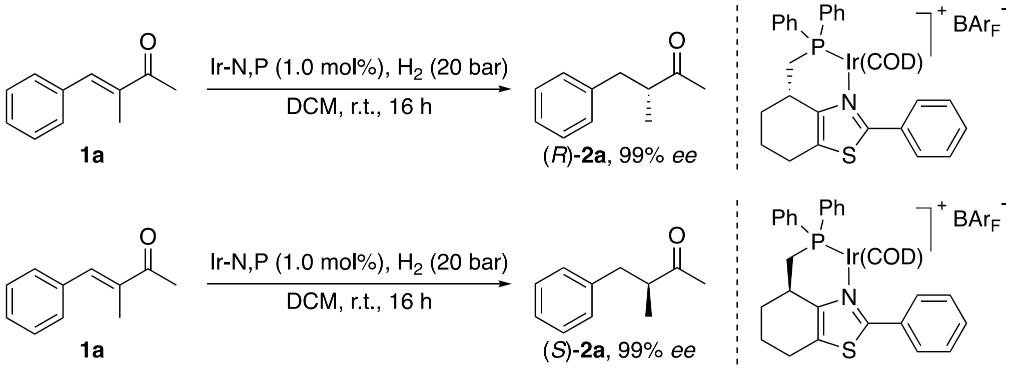

An oven-dried vial was charged with enone **1a** (32 mg, 0.2 mmol, 1.0 equiv.) and Ir-N,P-catalyst (1.6 mg, 0.5 mol%). Freshly distilled DCM (2 mL) and a magnetic stirring bar were added and the vial was placed in a high-pressure hydrogenation apparatus. The reactor was purged three times with Ar, purged three times with H_2 and then pressurized with H_2 (20 bar). The reaction was stirred at room temperature for 16 h before the H_2 pressure was released and the solvent was removed under reduced pressure. The residue was purified by flash chromatography (pentane/Et_2O, 50/50) on silica gel to give the ketone **2a**. The *ee* value was determined by GC analysis using Chiraldex β-DM stationary phase (80 °C iso, 90 min). The corresponding racemic product was used for comparison. The racemate was prepared by hydrogenation on a 0.1 mmol scale using Pd/C as the catalyst, following the same asymmetric hydrogenation procedure. The absolute configuration was determined by comparison of the optical rotation with reported values.

General procedure for the H/D exchange

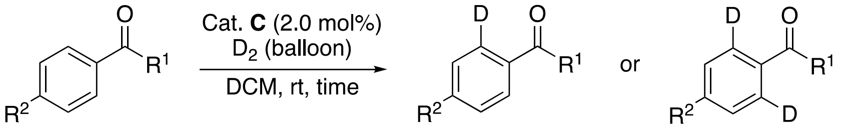

An oven-dried vial was charged with a benzoic acid derivative (0.05 mmol, 1.0 equiv.) and Ir-N,P-catalyst **C** (2.0 mol%). Freshly distilled DCM (1 mL) and a magnetic stirring bar were added and the vial was sealed with a septum. The atmosphere was evacuated and refilled with N_2 three times, evacuated and refilled with D_2 three times and then filled with D_2 (balloon). The reaction was stirred at room temperature for 3 or 5 h before the D_2 atmosphere was released and the solvent was removed under reduced pressure. The amount of C_{ortho}-H isotope exchange was measured by ^{1}H NMR analysis of the crude product in $CDCl_3$.

Control experiments H/D exchange

Control experiments in the presence of D_2O (5 equiv.) or *i*PrOD (5 equiv.) did not lead to any incorporation of deuterium in the C_{ortho} positions of benzamide, suggesting that the C_{ortho}-H bond activation is not mediated by any alcohol product formed during the hydrogenation.

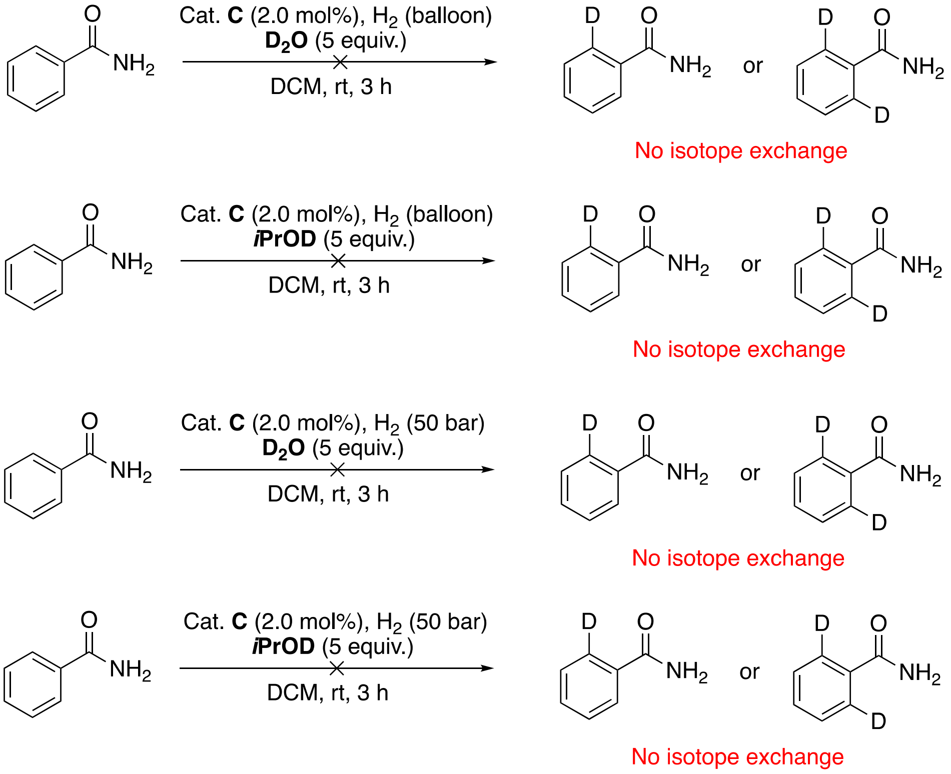

Control experiments using excess of benzamide

The hydrogenation of α-methyl styrene is complete in 2.5 h and under balloon pressure of hydrogen. When 1.0 equiv. of benzamide is added to the reaction, the hydrogenation rate decreases significantly to give 53% conv. after 2.5 h. This demonstrates that the hydrogenation rate is of olefins is negatively affected by the presence of benzamide in large quantities.

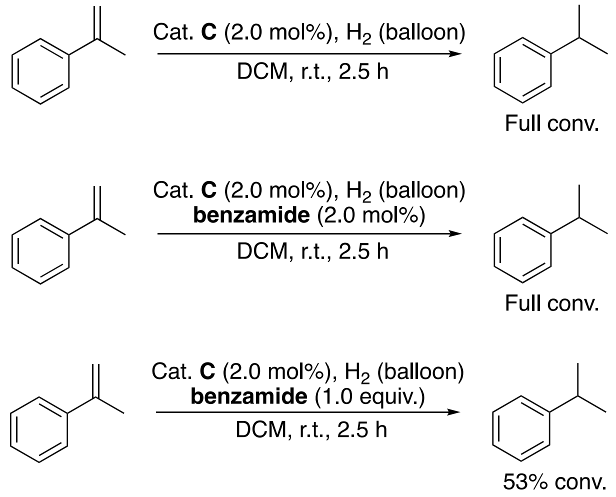

Reaction conditions: 0.05 mmol of substrate, 2.0 mol% catalyst, 2.0 mol% or 1 equiv. of benzamide, 1 mL DCM, balloon pressure H_2, 2.5 h, rt. Conversion was determined by ^{1}H NMR spectroscopy.

Control experiments using other hydrogenation catalysts

The asymmetric hydrogenation of ketones is not promoted for catalysts that are unable to hydrogenate the ketone in the absence of benzamide. In all cases, complete conversion towards ketone **2a** was obtained, regardless of the addition of benzamide (1 mol%).

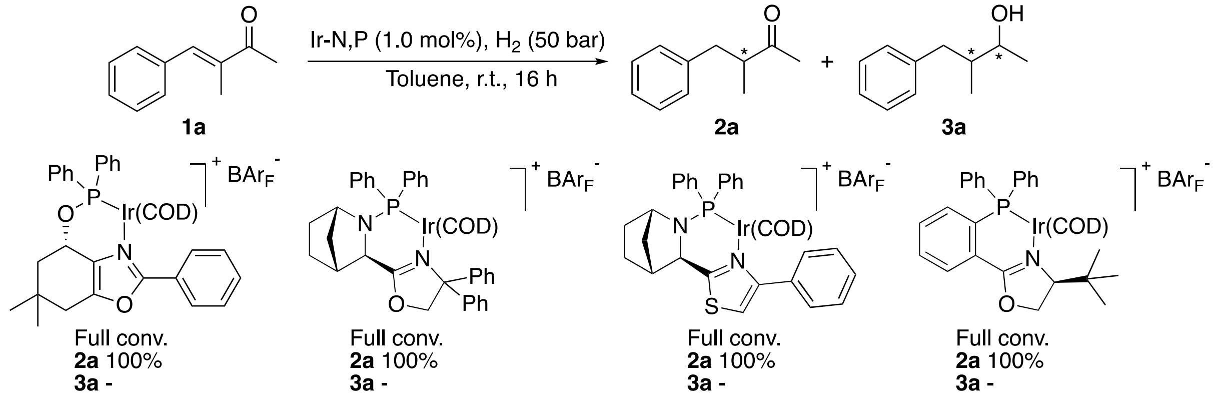

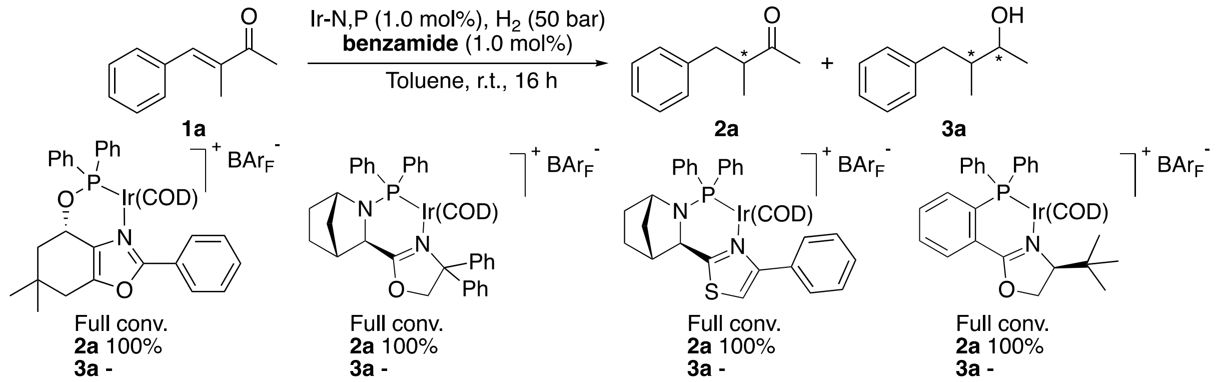

Reaction conditions: 0.05 mmol of substrate, 1.0 mol% catalyst, 1.0 mol% benzamide, 1 mL toluene, 50 bar H_2, 16 h, rt. Conversion was determined by ^{1}H NMR spectroscopy.

References

1 Peters, B. B. C.; Jongcharoenkamol, J.; Krajangsri, S.; Andersson, P. G. *Org. Lett.* **2021**, *23*, 242-246.

2 Peters, B. B. C.; Zheng, J.; Birke, N.; Singh, T.; Andersson, P. G. *Nat. Commun.* **2022**, *13*, 361.

3 Lu, S.-M.; Bolm, C. *Angew. Chem. Int. Ed.* **2008**, *47*, 8920-8923.

4 Grant, T. N.; West, F. G. *Org. Lett.* **2007**, *9*, 3789-3792.

5 Kaukoranta, P.; Engman, M.; Hedberg, C.; Bergquist, J.; Andersson, P. G. *Adv. Synth. Catal.* **2008**, *350,* 1168-1176.

6 Peters, B. K.; Liu, J.; Margarita, C.; Rabten, W.; Kerdphon, S.; Orebom, A.; Morsch, T.; Andersson, P. G. *J. Am. Chem. Soc.* **2016**, *138*, 11930-11935.

7 Liu, J.; Krajangsri, S.; Yang, J. Li, J.-Q.; Andersson, P. G. *Nat. Catal.* **2018**, *1*, 438-443.

8 Akeboshi, T.; Ohtsuka, Y.; Ishihara, T.; Sugai, T. *Adv. Synth. Catal.* **2001**, *343*, 624-637.

NMR spectra – Enones

^{1}H NMR (400 MHz, $CDCl_3$)

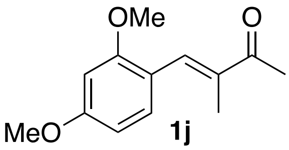

1j

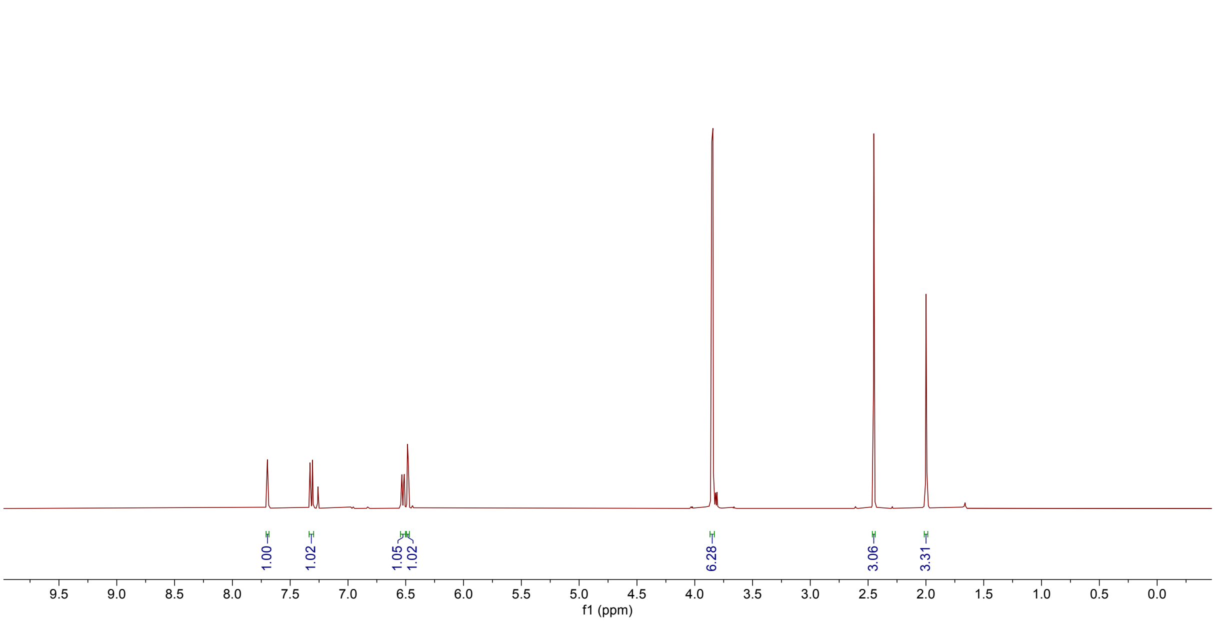

^{13}C NMR (100 MHz, $CDCl_3$)

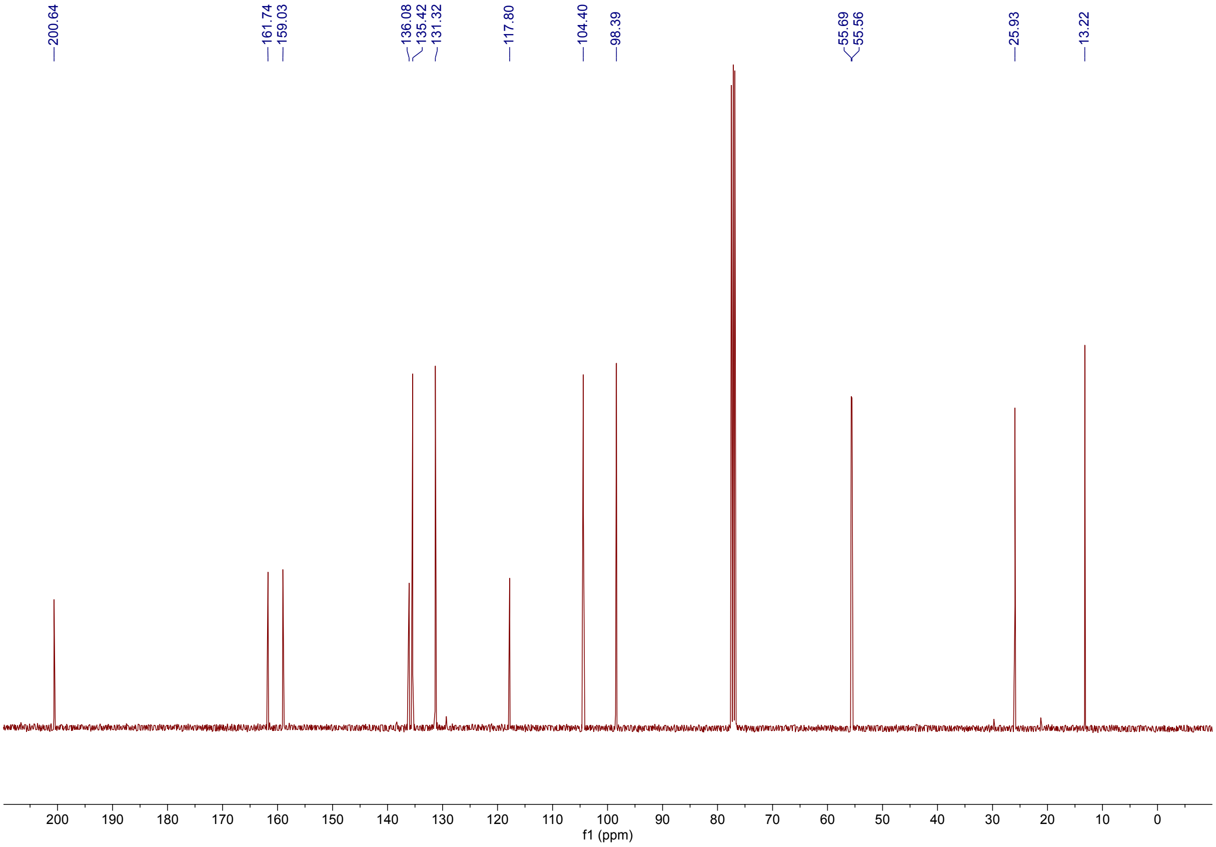

NMR spectra – Hydrogenated products

^{1}H NMR (400 MHz, $CDCl_3$)

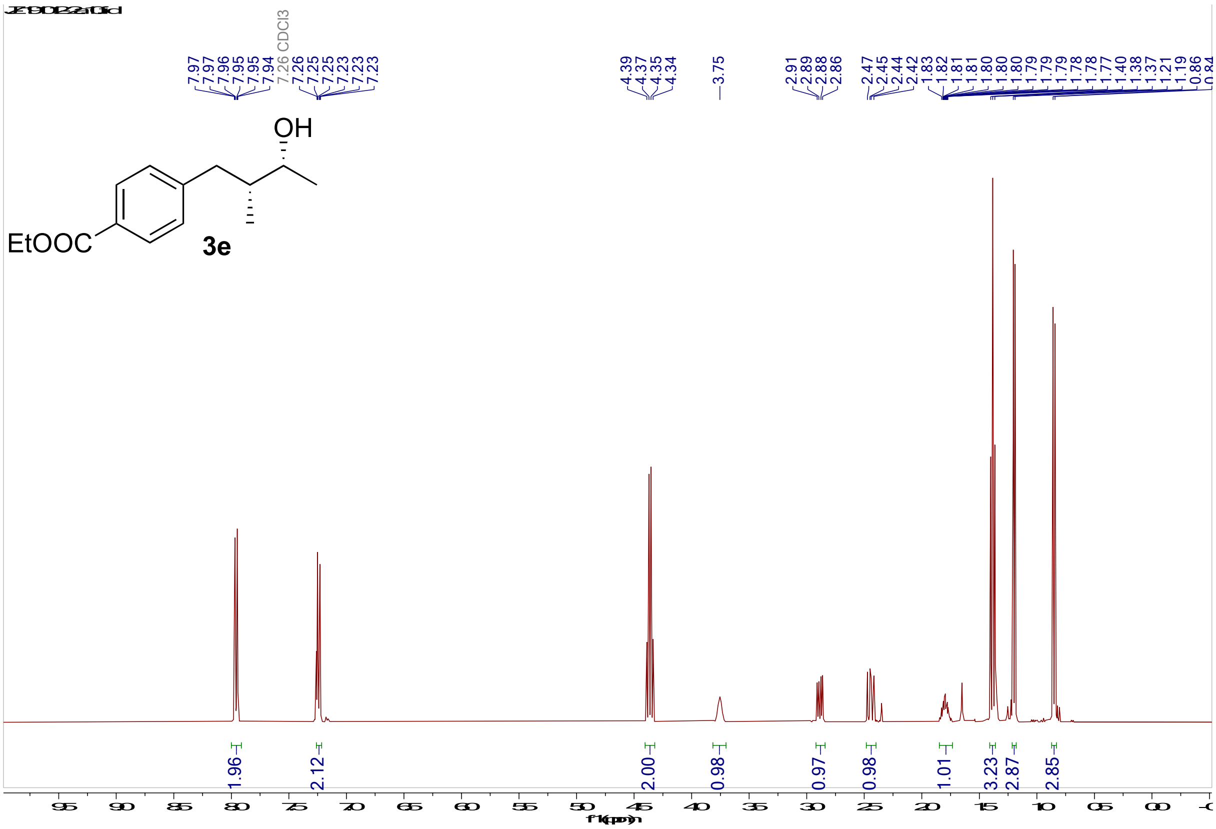

^{13}C NMR (100 MHz, $CDCl_3$)

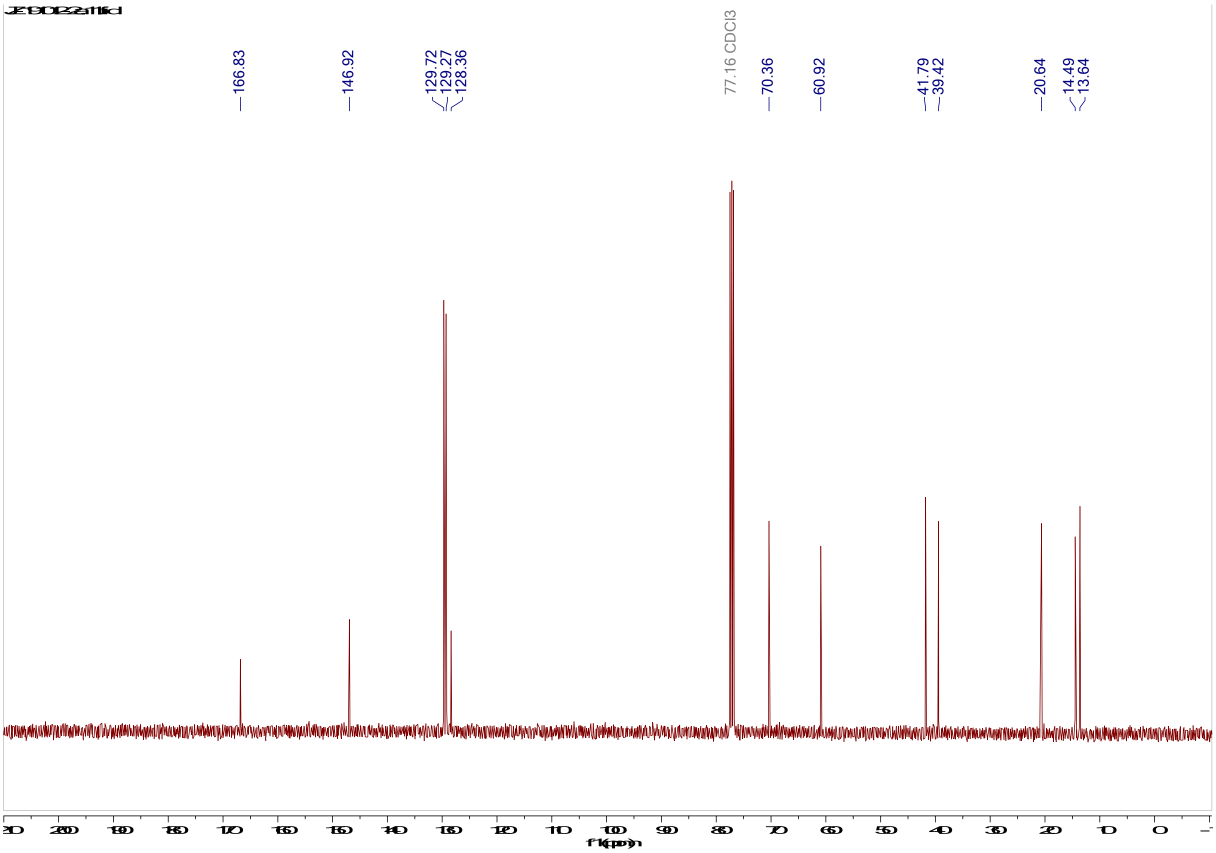

^{1}H NMR (400 MHz, $CDCl_3$)

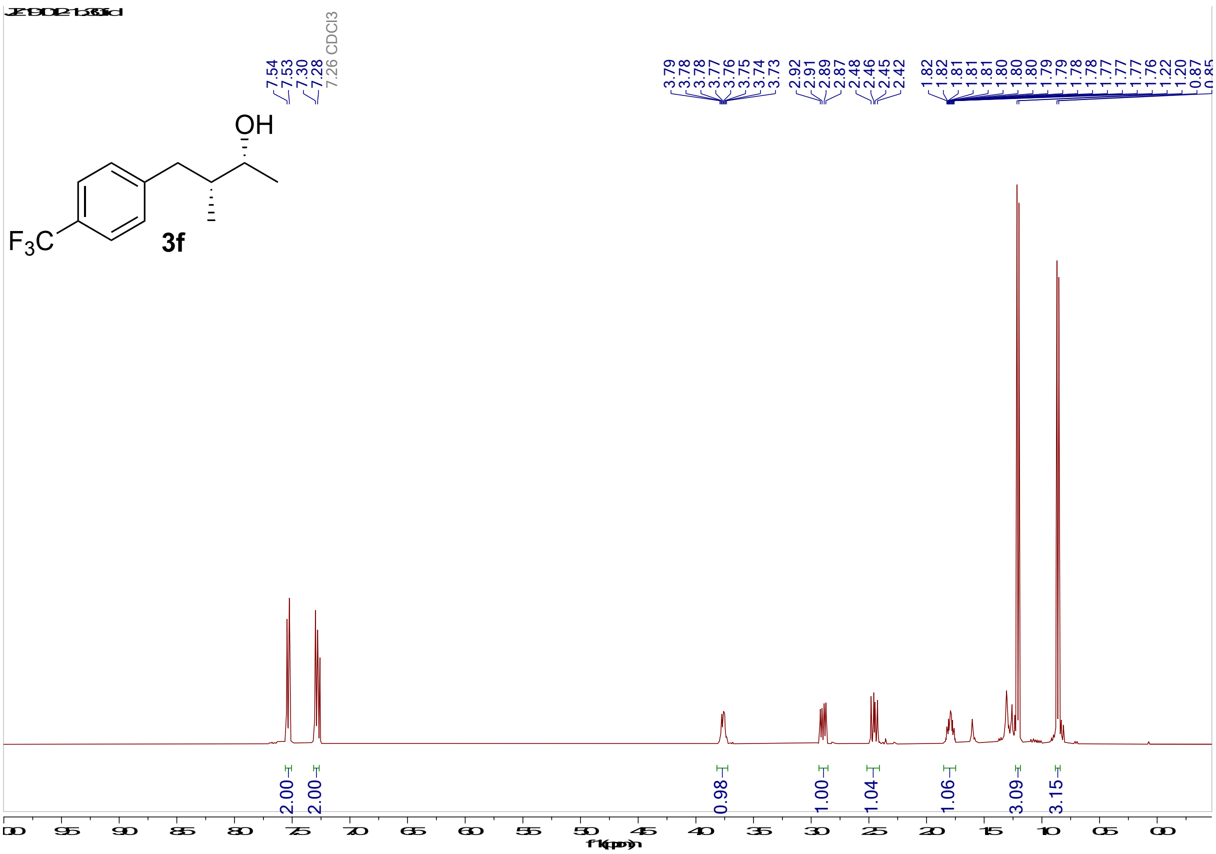

^{13}C NMR (100 MHz, $CDCl_3$)

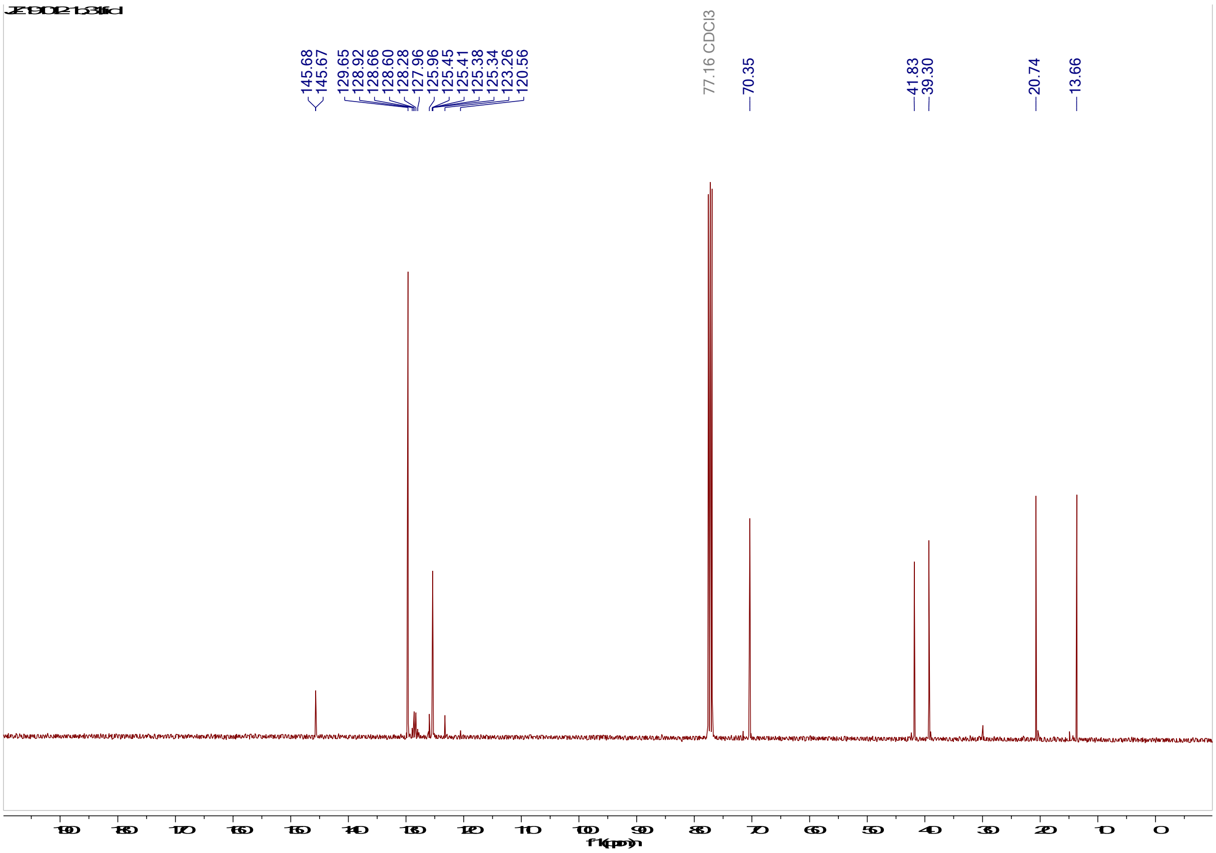

^{19}F NMR (377 MHz, $CDCl_3$)

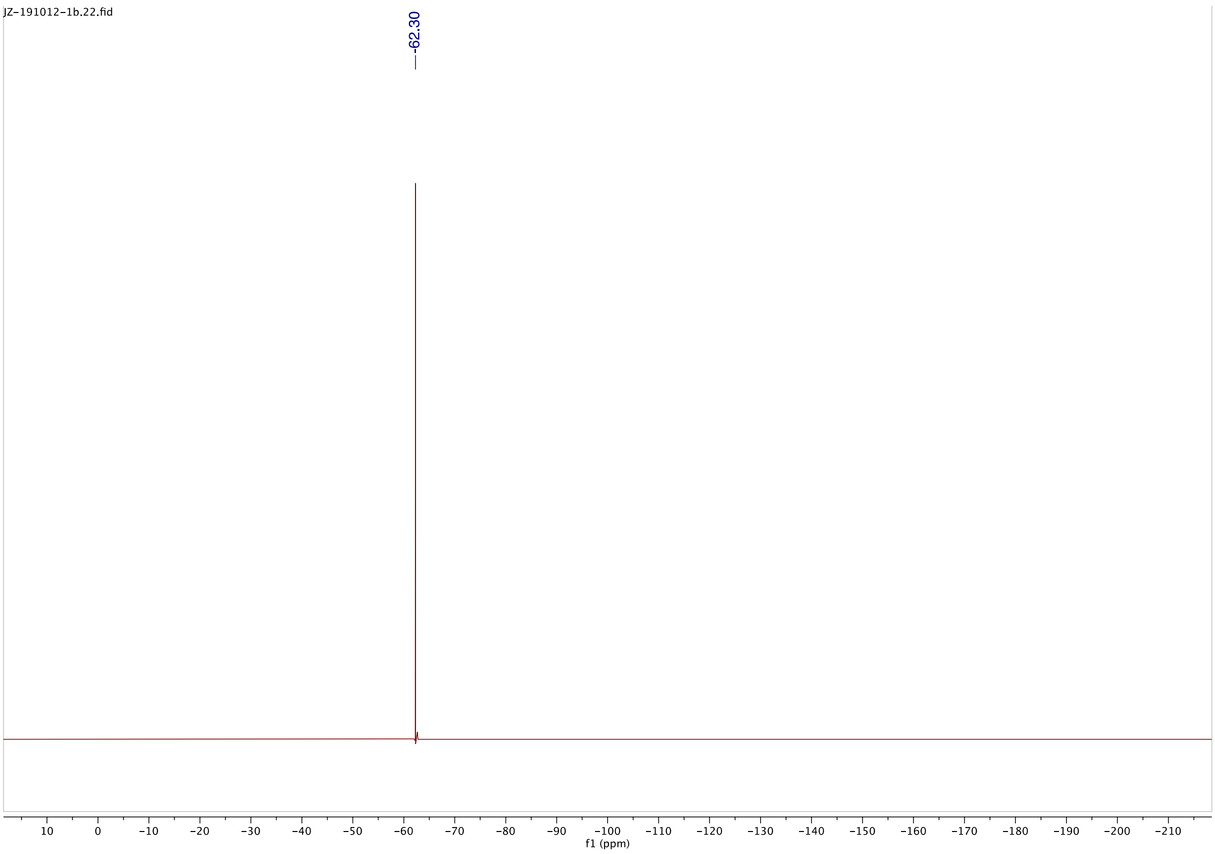

^{1}H NMR (400 MHz, $CDCl_3$)

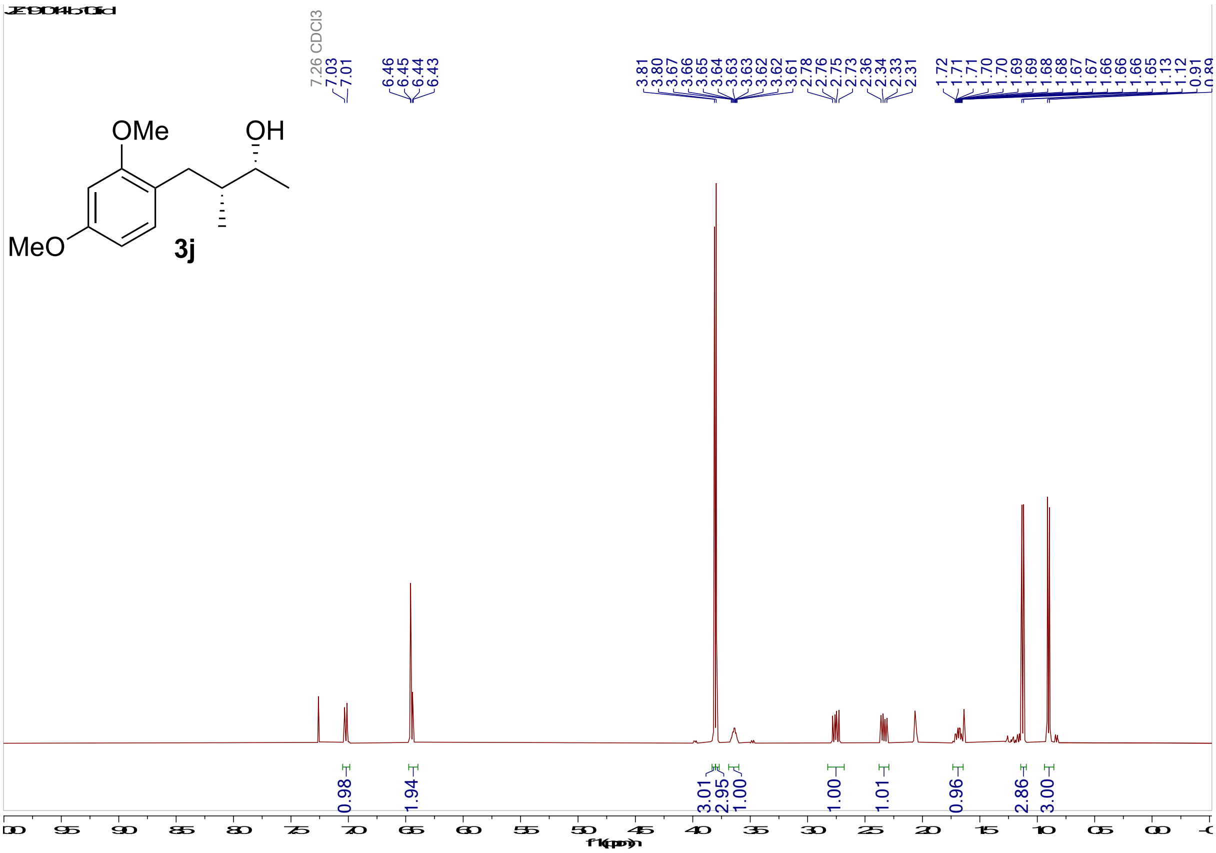

^{13}C NMR (100 MHz, $CDCl_3$)

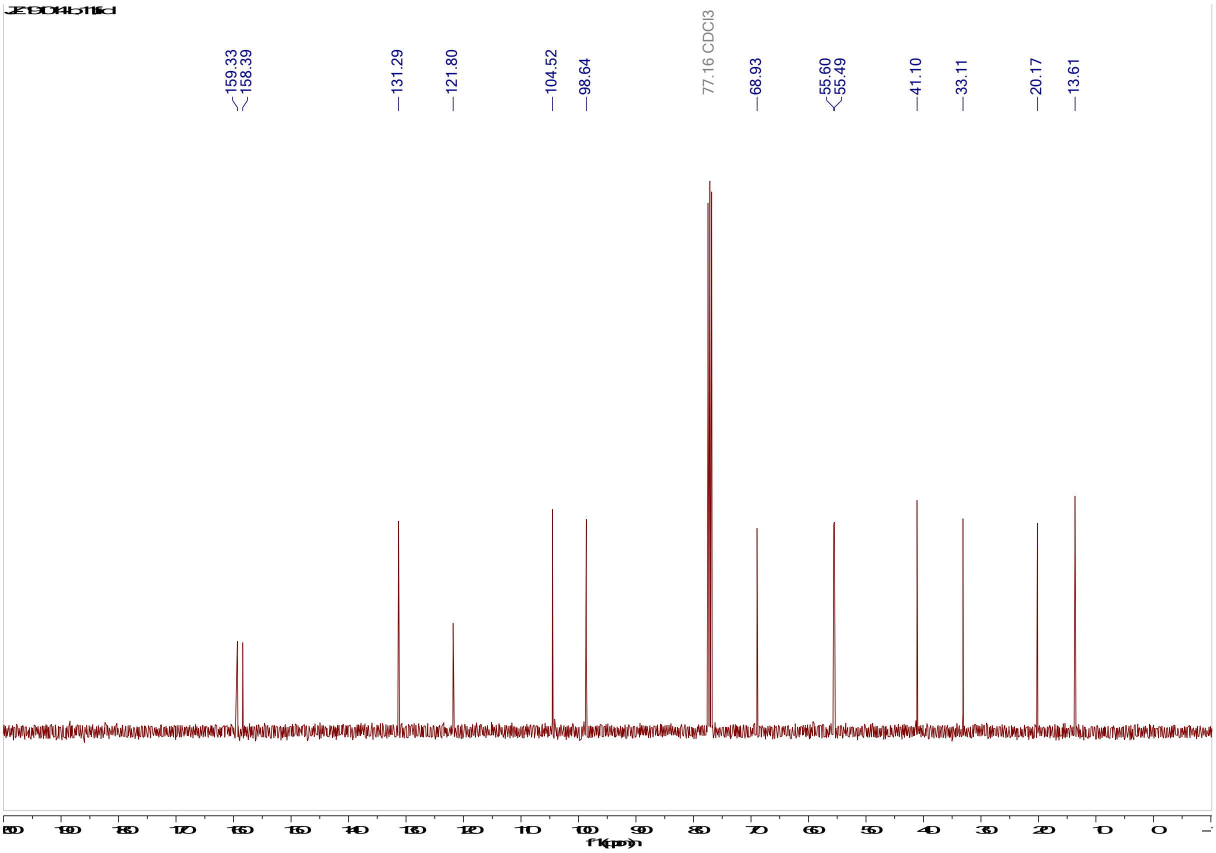

^{1}H NMR (400 MHz, $CDCl_3$)

3k

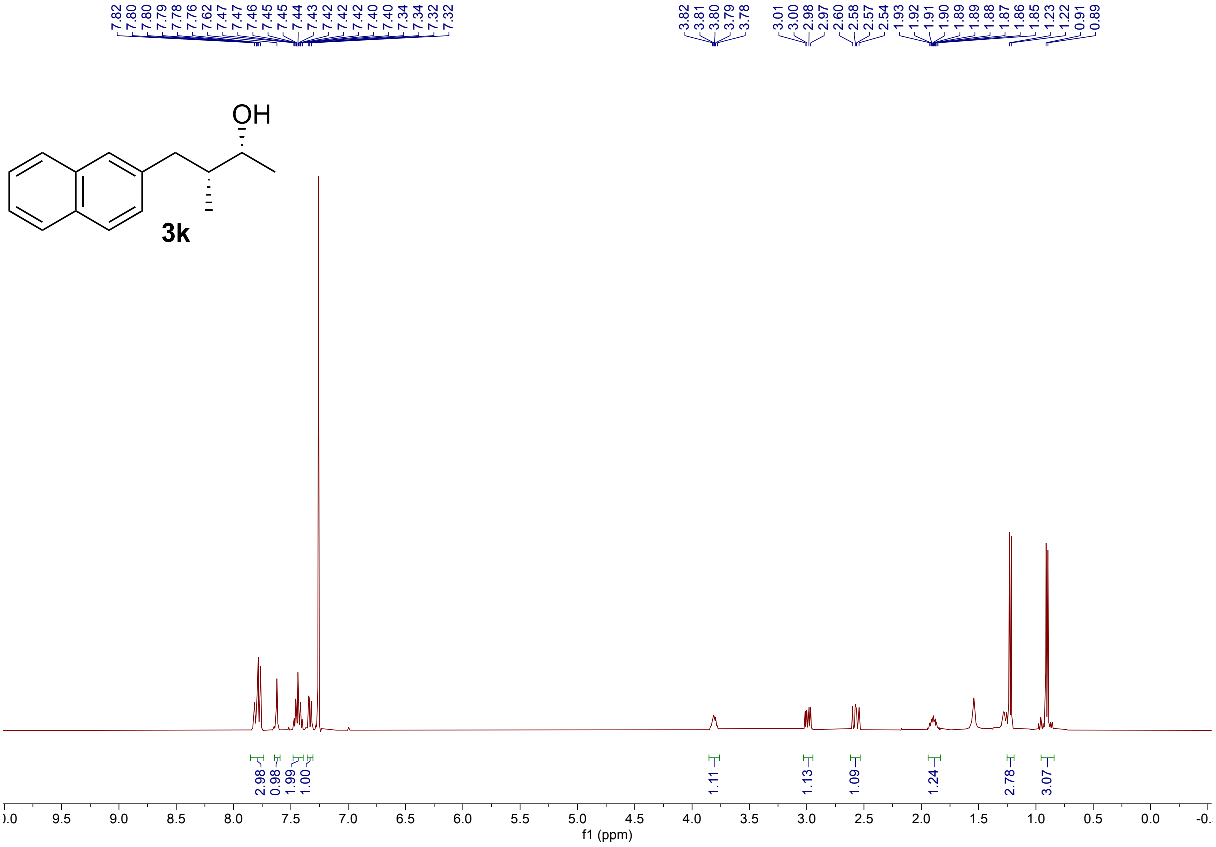

^{13}C NMR (100 MHz, $CDCl_3$)

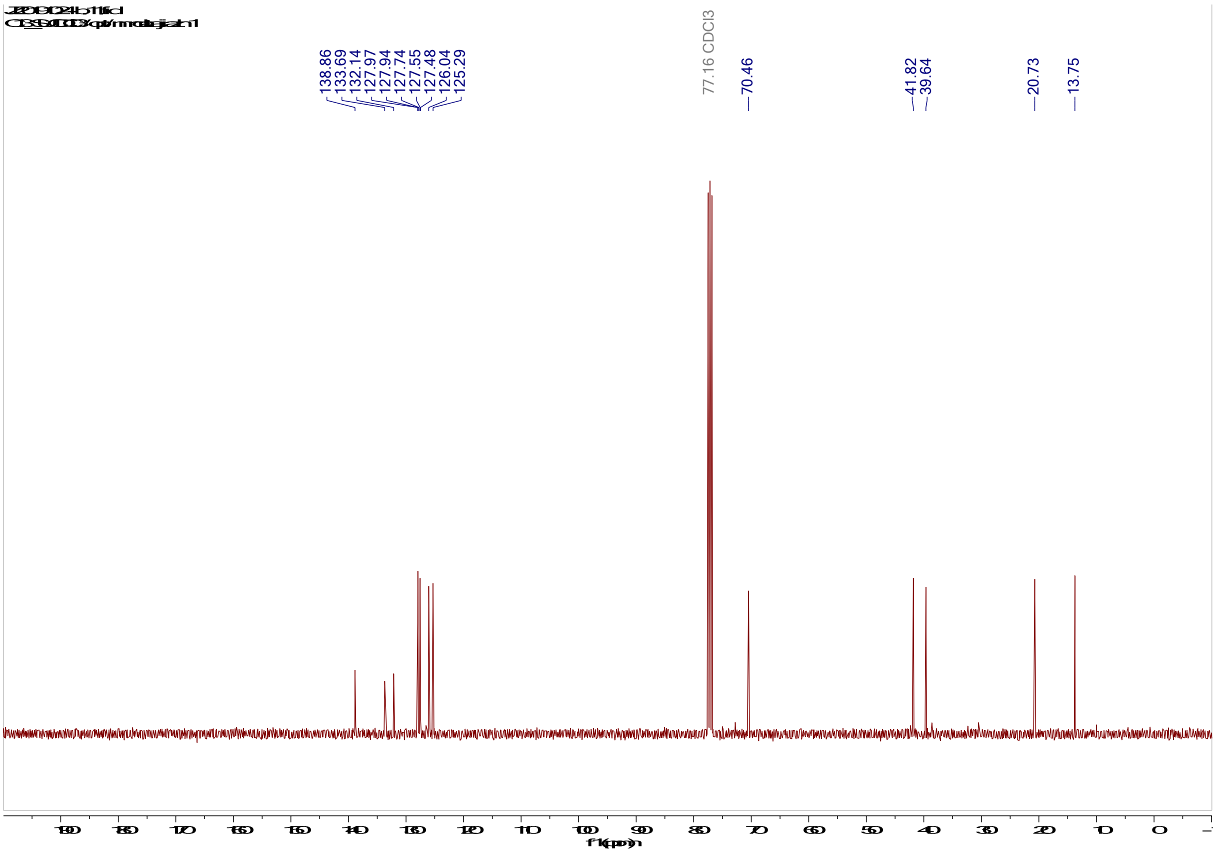

^{1}**H NMR** (400 MHz, $CDCl_3$)

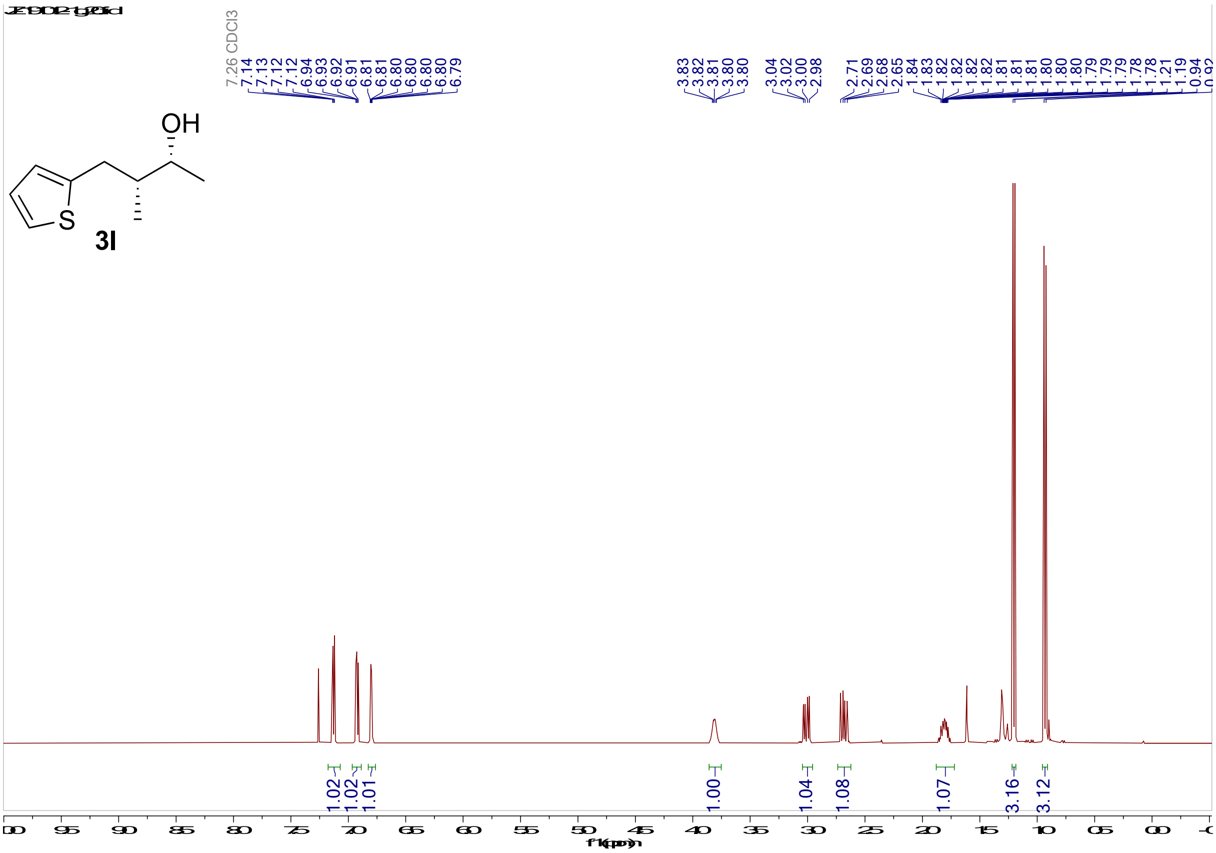

^{13}**C NMR** (100 MHz, $CDCl_3$)

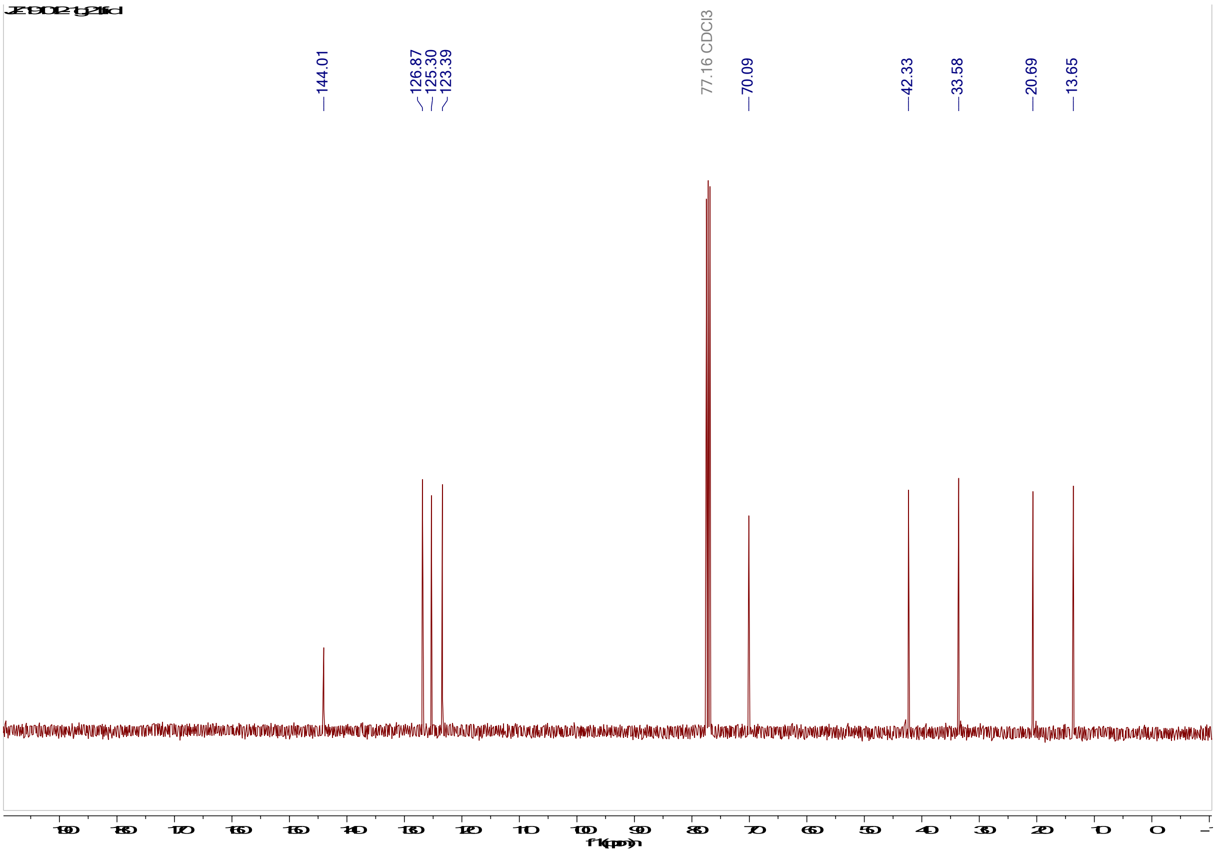

^{1}H NMR (400 MHz, $CDCl_3$)

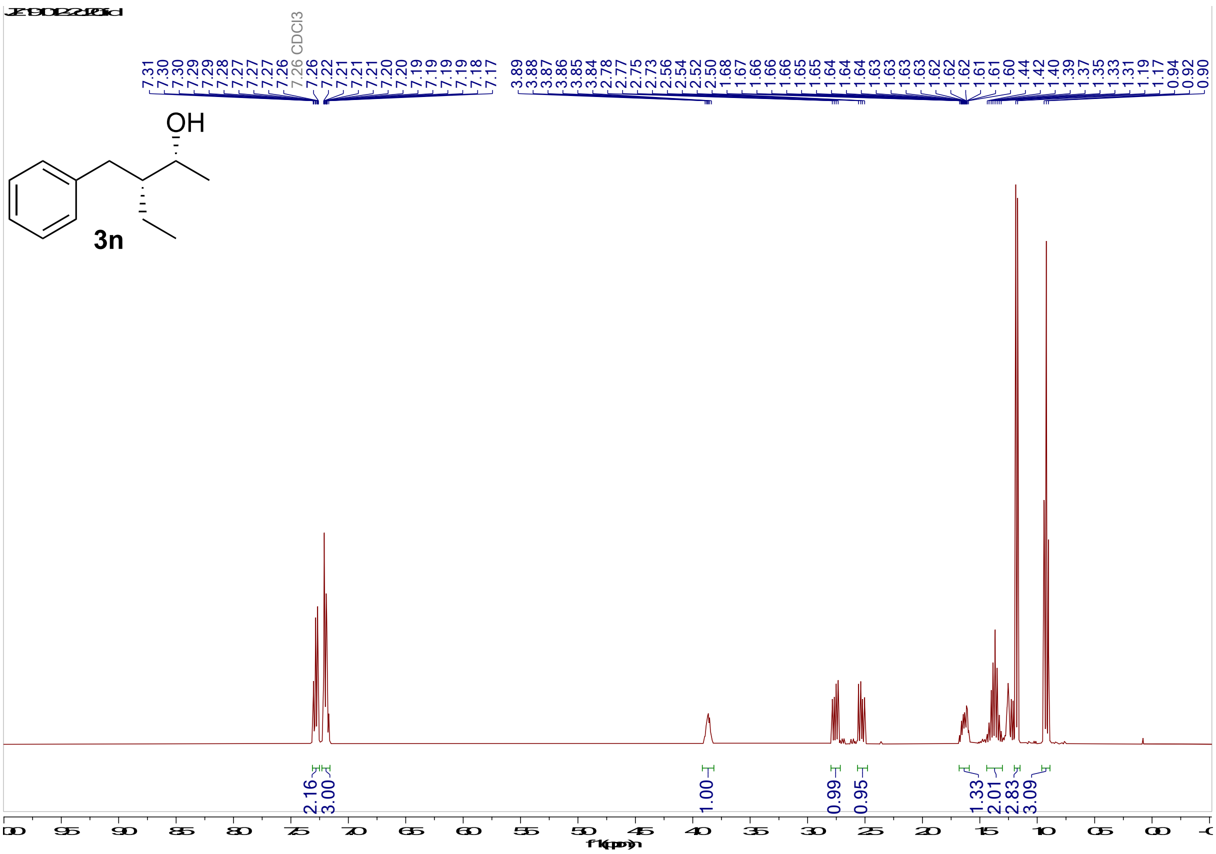

^{13}C NMR (100 MHz, $CDCl_3$)

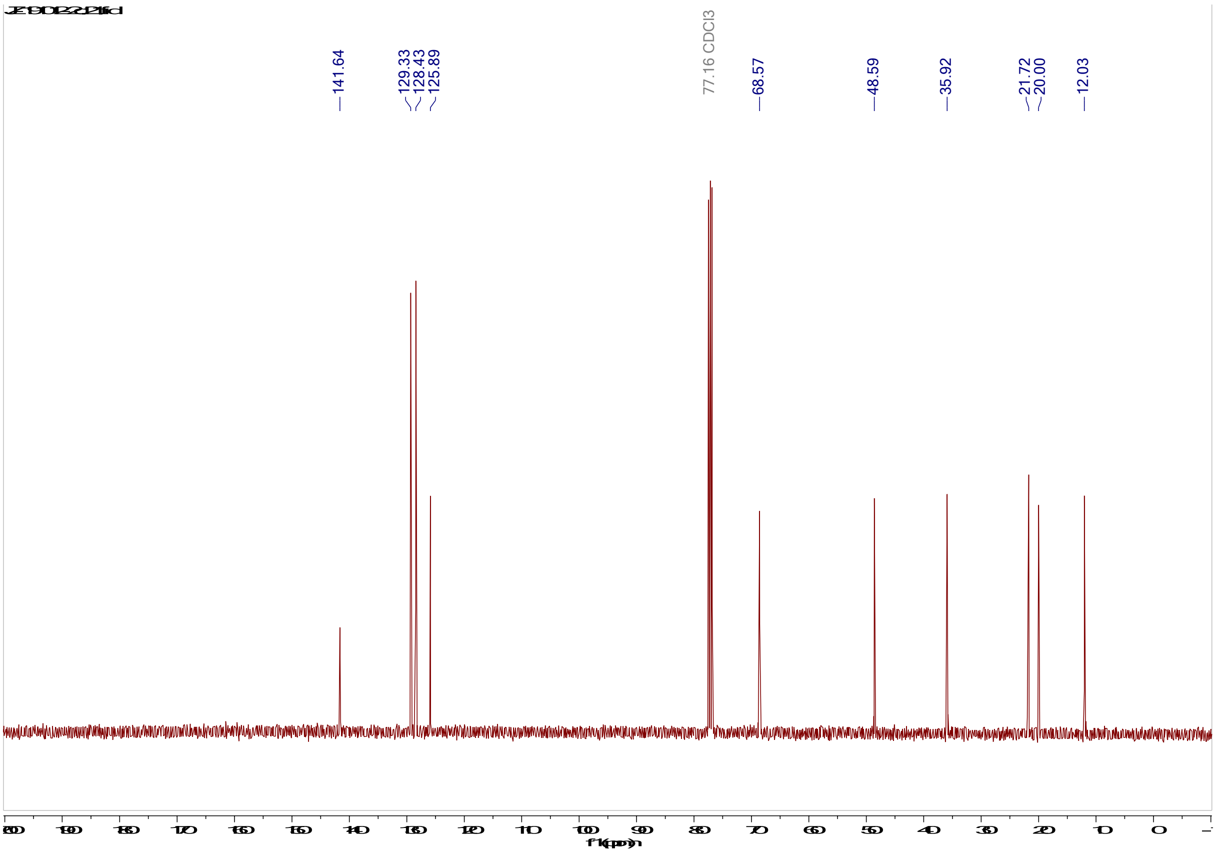

^{1}**H NMR** (400 MHz, $CDCl_3$)

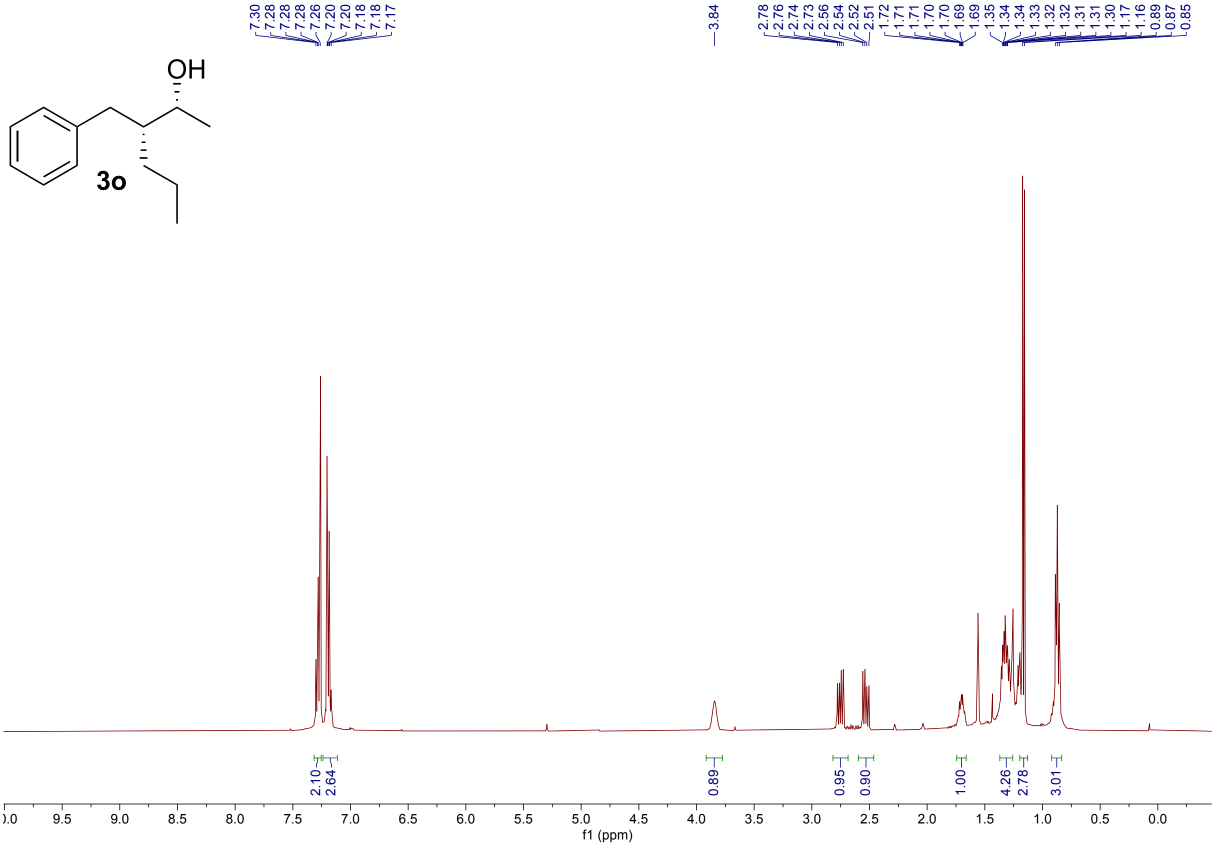

^{13}**C NMR** (100 MHz, $CDCl_3$)

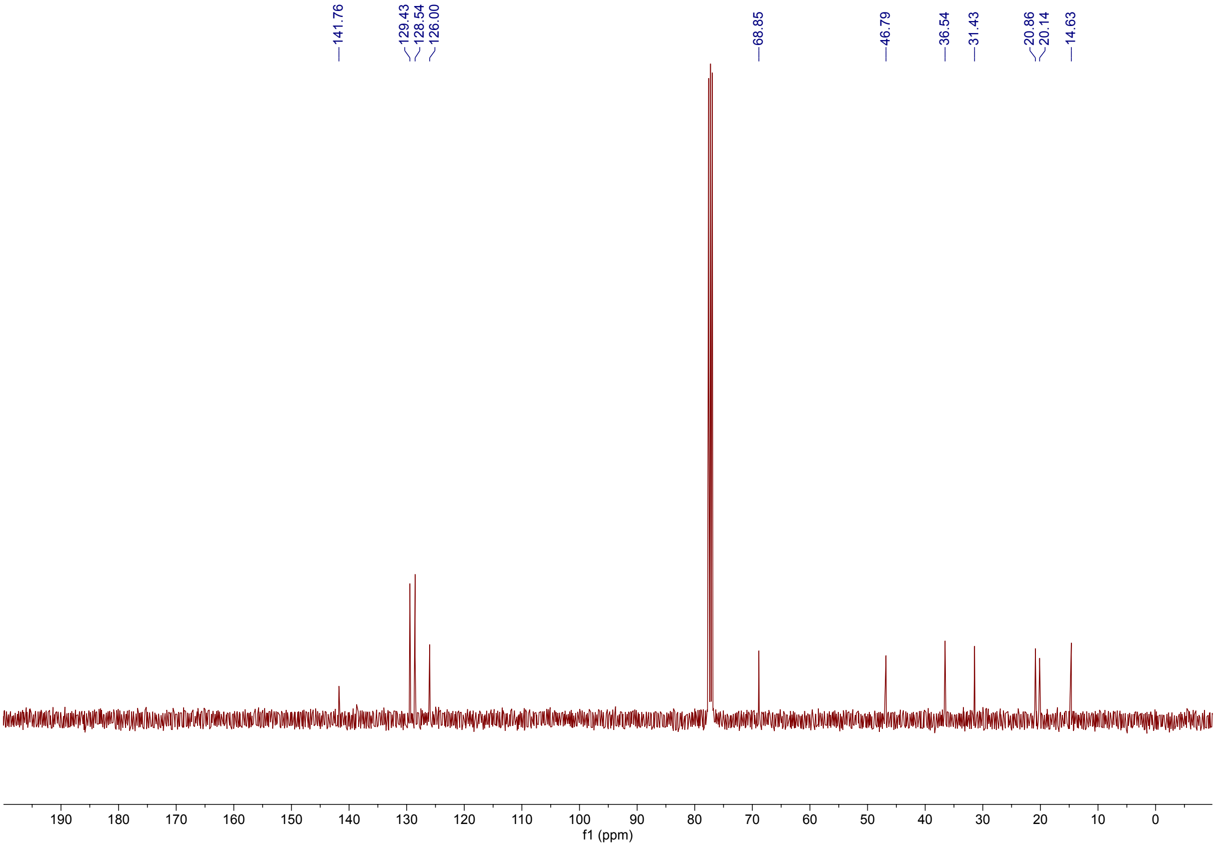

NMR spectra – Catalyst C and intermediates

^{1}H NMR (400 MHz, $CDCl_3$)

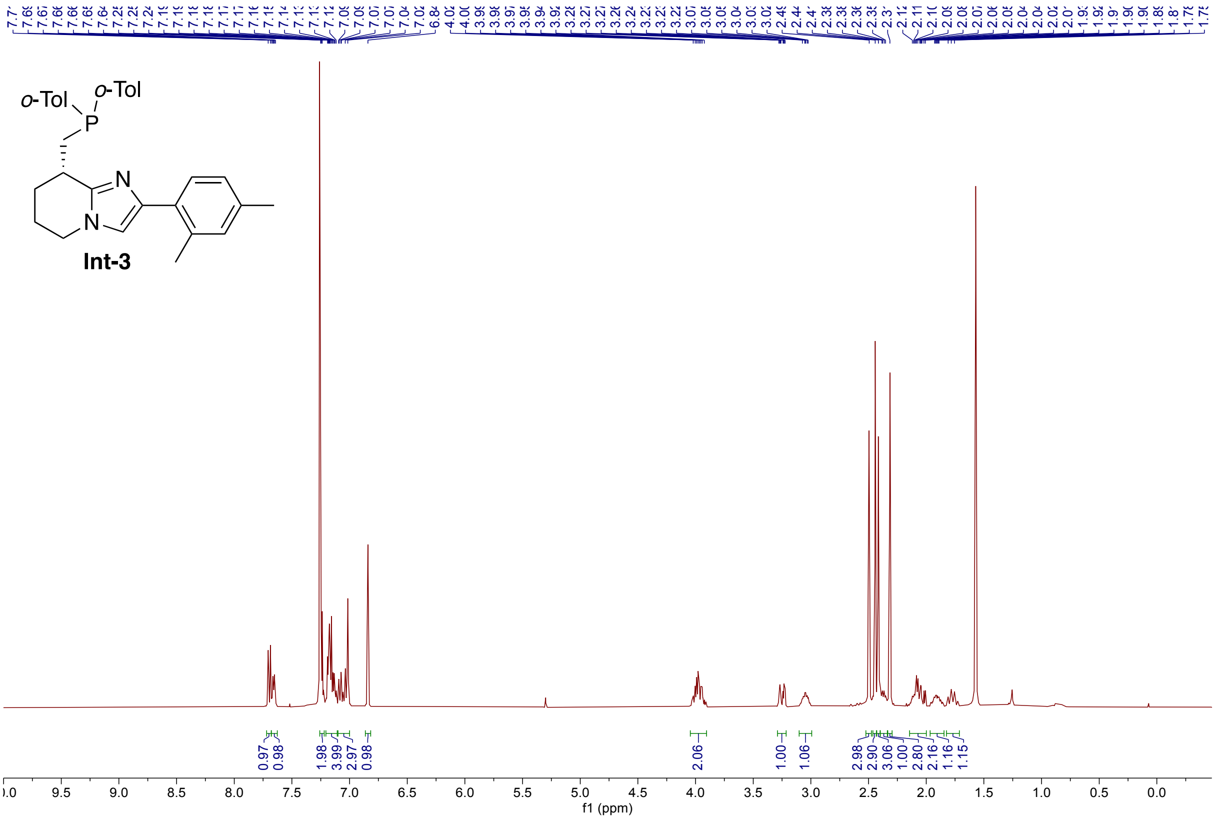

^{13}C NMR (100 MHz, $CDCl_3$)

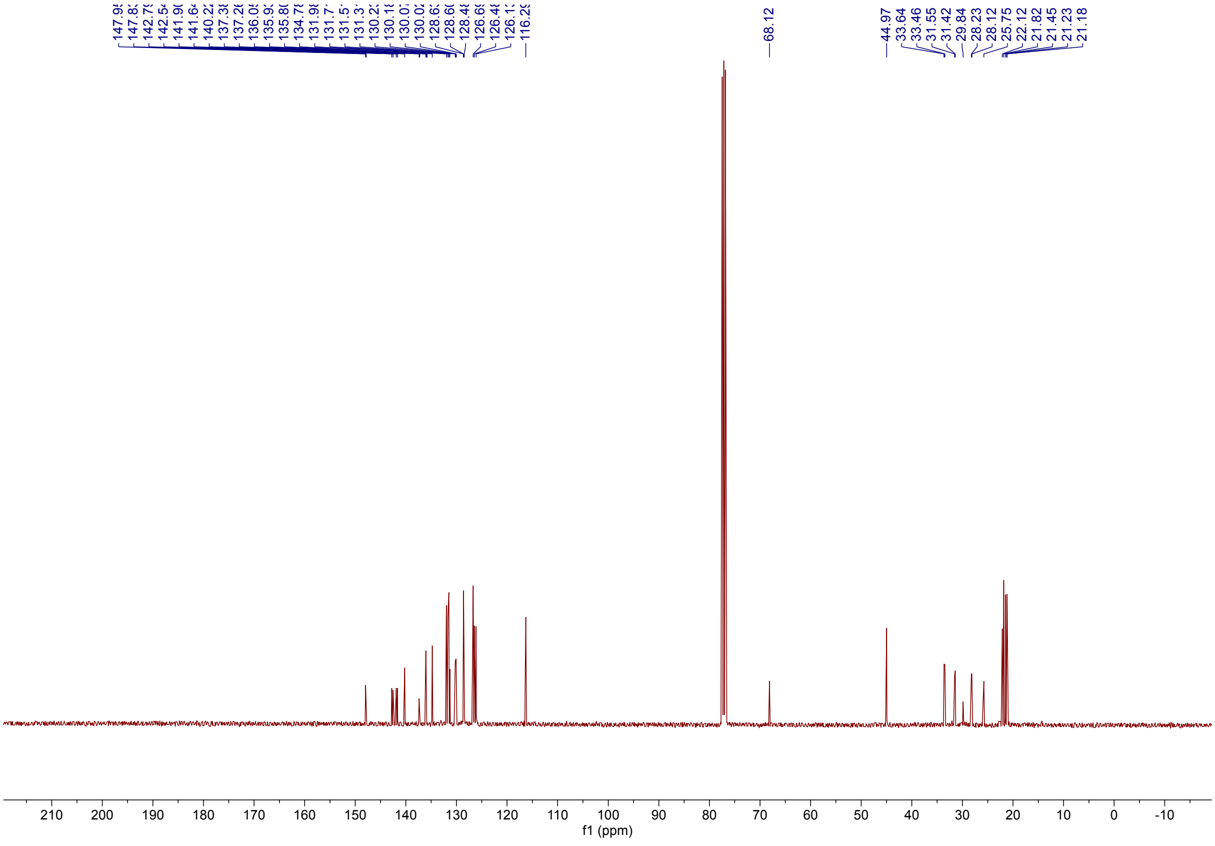

^{31}P NMR (162 MHz, $CDCl_3$)

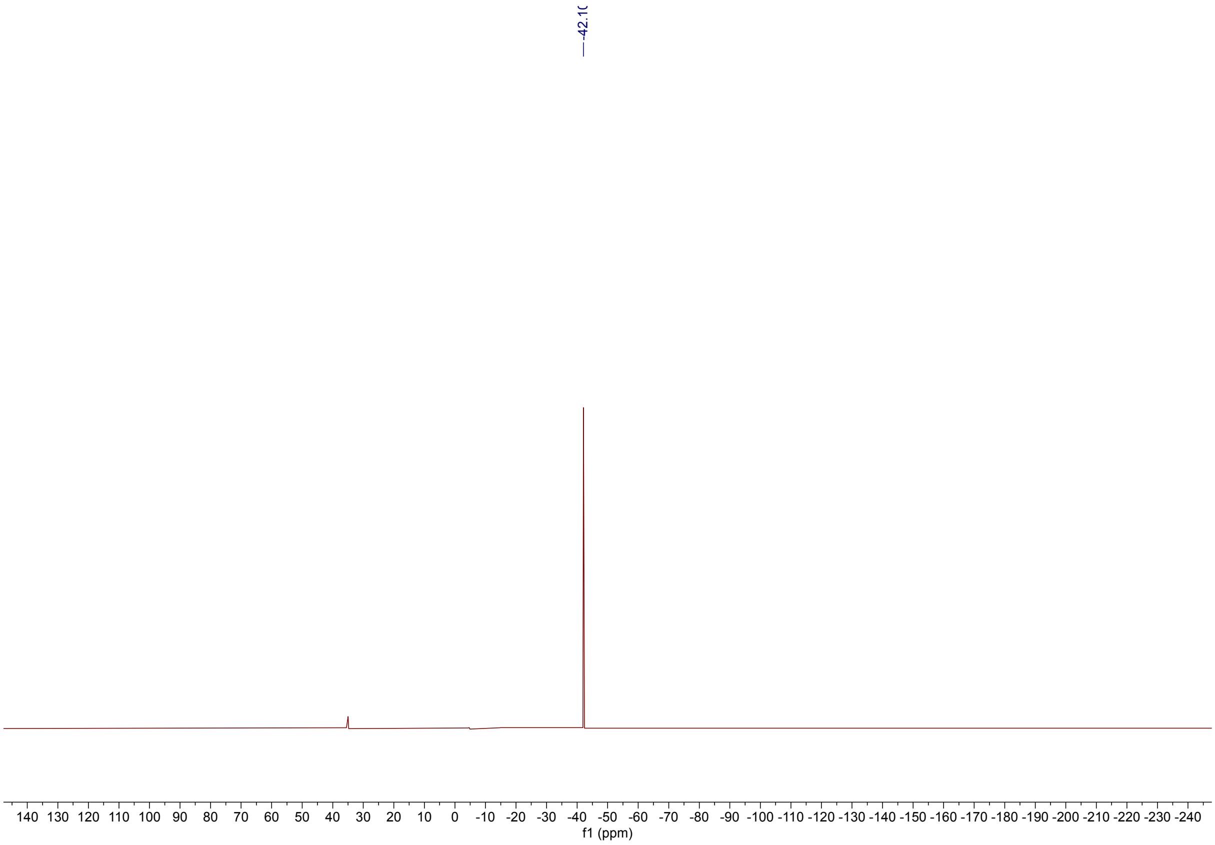

^{1}H NMR (400 MHz, $CDCl_3$)

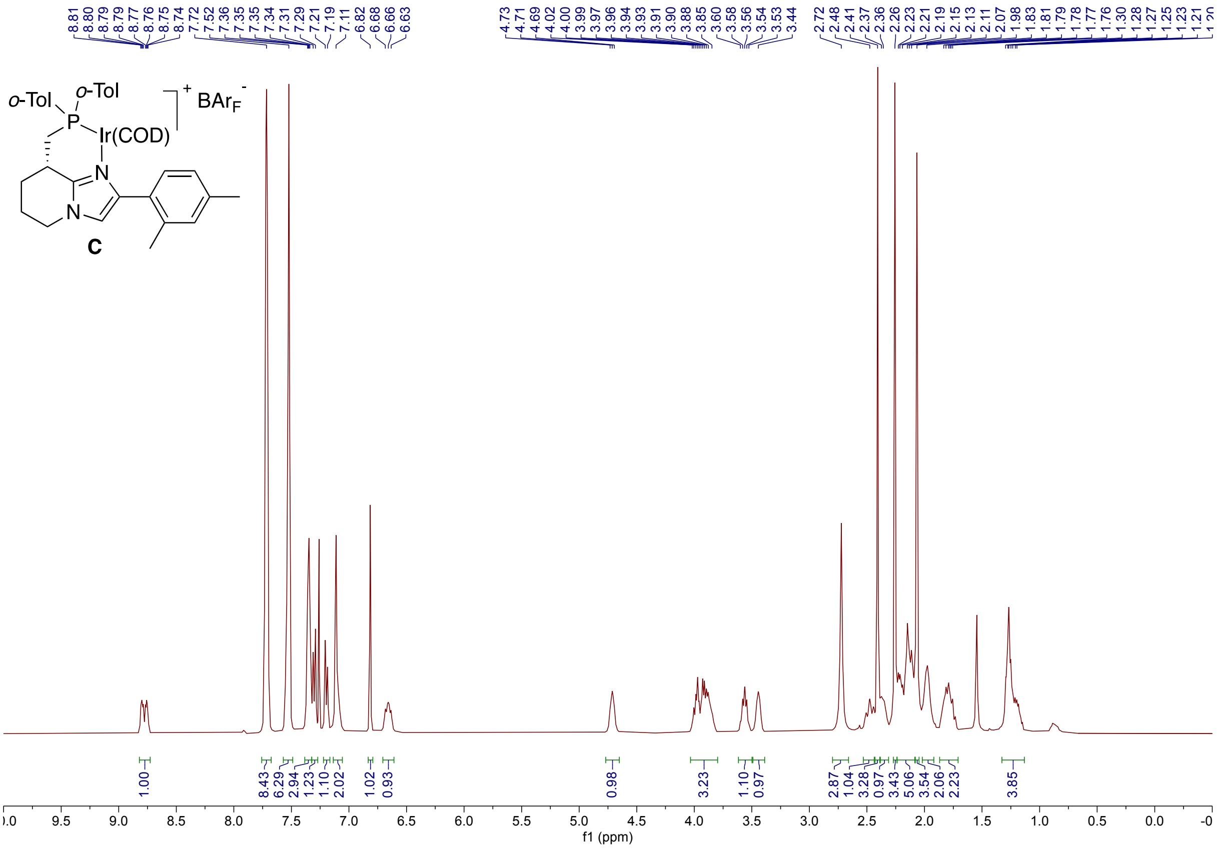

^{13}C NMR (100 MHz, $CDCl_3$)

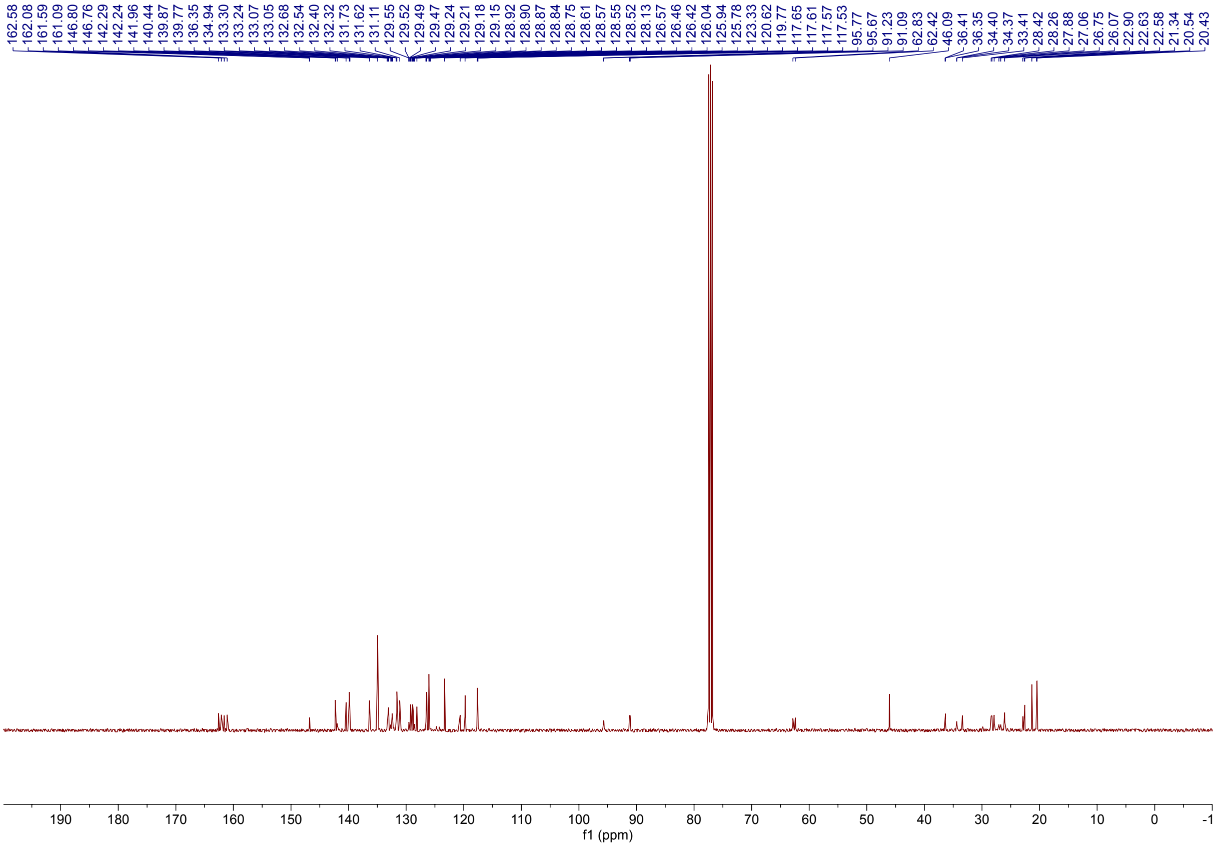

^{31}P NMR (162 MHz, $CDCl_3$)

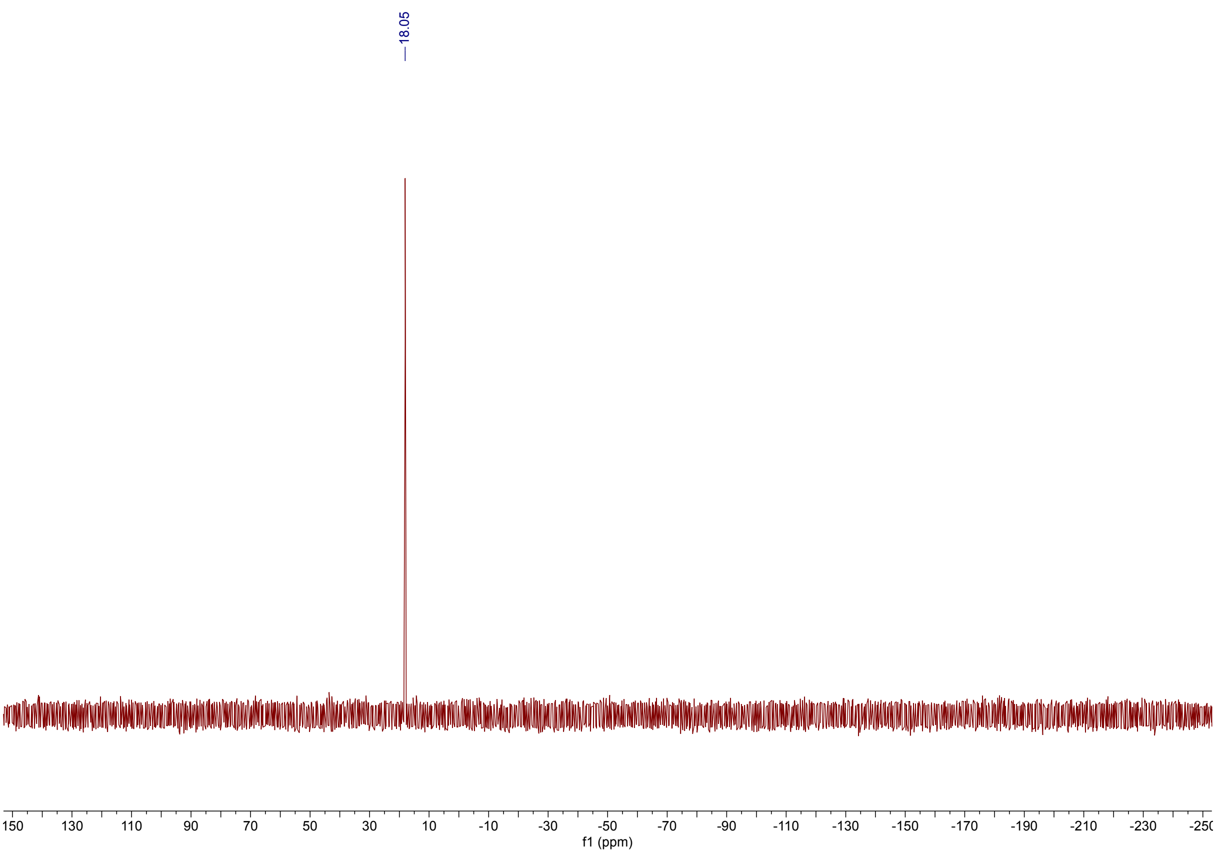

^{19}F NMR (377 MHz, $CDCl_3$)

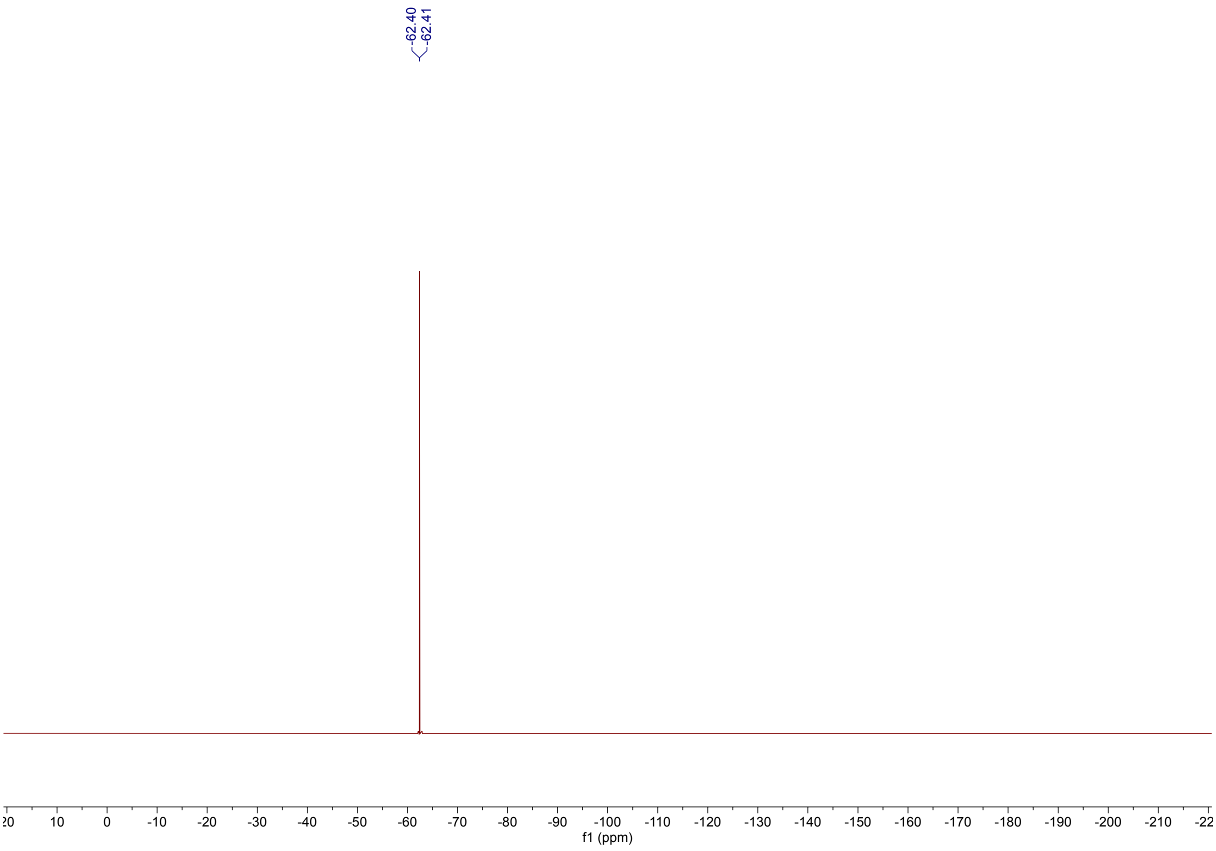

Separation of chiral products

Entry	Product	Separation method	Optical rotation	*ee* (%), d.r.
1	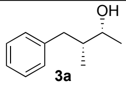 3a	GC-MS: Chiraldex β-DM column (90 iso 120 min), t_R = 40.7 min (major) / 41.9 min (minor) / 44.9 min (minor) / 46.5 min (minor)	$[\alpha]_D^{26}$ = + 8 (c = 0.1, $CHCl_3$)	99, 99/1
2	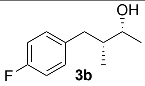 3b	GC-MS: Chiraldex β-DM column (80 iso 200 min), t_R = 99.2 min (major) / 105.4 min (minor) / 111.2 min (minor) / 115.2 min (minor)	$[\alpha]_D^{26}$ = + 8 (c = 0.1, $CHCl_3$)	99, 99/1
3	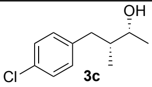 3c	SFC: Chiralpak IC column (97% CO_2 3% MeOH), 2.0 mL/min, t_R = 18.2 min (minor) / 19.0 min (major) / 20.4 min (minor) / 22.1 min (minor)	$[\alpha]_D^{26}$ = + 9 (c = 0.1, $CHCl_3$)	99, 98/2
4	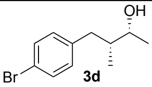 3d	GC-MS: Chiraldex β-DM column (110 iso 120 min), t_R = 69.4 min (major) / 73.9 min (minor) / 76.3 min (minor) / 78.5 min (minor)	$[\alpha]_D^{26}$ = + 9 (c = 0.1, $CHCl_3$)	99, 99/1
5	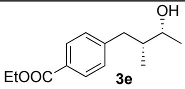 3e	SFC: Chiralpak IC column (95% CO_2 5% MeOH), 2.0 mL/min, t_R = 53.1 min (minor) / 57.7 min (major) / 60.3 min (minor) / 64.7 min (minor)	$[\alpha]_D^{27}$ = + 10 (c = 0.1, $CHCl_3$)	99, 99/1
6	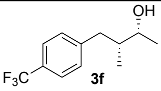 3f	GC-MS: Chiraldex β-DM column (100 iso 200), t_R = 33.2 min (major) / 35.3 min (minor) / 36.3 min (minor) / 38.6 min (minor)	$[\alpha]_D^{26}$ = + 6 (c = 0.1, $CHCl_3$)	99, 99/1
7	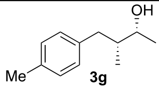 3g	SFC: Chiralcel AY-H column (95% CO_2 5% MeOH), 2.0 mL/min, t_R = 6.4 min (minor) / 6.9 min (minor) / 7.7 min (major) / 8.2 min (minor)	$[\alpha]_D^{27}$ = + 10 (c = 0.1, $CHCl_3$)	99, 99/1
8	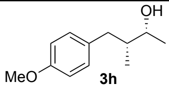 3h	SFC: Chiralcel OJ-H column (90% CO_2 10% MeOH), 2.0 mL/min, t_R = 8.8 min (major) / 9.5 min (minor) / 10.1 min (minor) / 11.0 min (minor)	$[\alpha]_D^{26}$ = + 8 (c = 0.1, $CHCl_3$)	99, 99/1
9	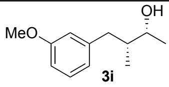 3i	SFC: Chiralpak IB column (90% CO_2 10% MeOH), 2.0 mL/min, t_R = 9.4 min (minor) / 10.7 min (major) / 11.1 min (minor) / 13.0 min (minor)	$[\alpha]_D^{27}$ = + 11 (c = 0.1, $CHCl_3$)	99, 99/1
10	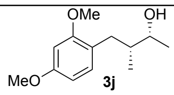 3j	SFC: Chiralpak IC column (95% CO_2 5% MeOH), 2.0 mL/min, t_R = 10.1 min (minor) / 10.9 min (major) / 13.5 min (minor) / 14.0 min (minor)	$[\alpha]_D^{27}$ = + 1 (c = 0.1, $CHCl_3$)	99, 97/3

11	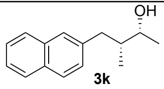 3k	SFC: Chiralpak IC column (90% CO_2 10% MeOH), 2.0 mL/min, t_R = 18.1 min (minor) / 20.2 min (major) / 21.4 min (minor) / 22.7 min (minor)	$[\alpha]_D^{26}$ = + 4 (c = 0.1, $CHCl_3$)	99, 99/1
12	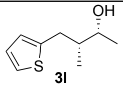 3l	SFC: Chiralpak IC column (95% CO_2 5% MeOH), 2.0 mL/min, t_R = 6.2 min (minor) / 6.7 min (major) / 7.1 min (minor) / 7.5 min (minor)	$[\alpha]_D^{27}$ = + 12 (c = 0.1, $CHCl_3$)	99, 95/5
13	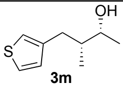 3m	SFC: Chiralpak IC column (95% CO_2 5% MeOH), 2.0 mL/min, t_R = 7.0 min (major) / 7.3 min (minor) / 7.6 min (minor) / 8.0 min (minor)	$[\alpha]_D^{27}$ = + 10 (c = 0.1, $CHCl_3$)	99, 98/2
14	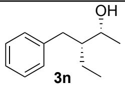 3n	SFC: Chiralcel OD-H column (90% CO_2 10% MeOH), 2.0 mL/min, t_R = 7.3 min (minor) / 8.3 min (minor) / 8.9 min (major) / 9.8 min (minor)	$[\alpha]_D^{27}$ = - 7 (c = 0.1, $CHCl_3$)	95, 97/3
15	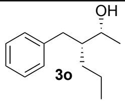 3o	SFC: Chiralcel OD-H column (95% CO_2 5% MeOH), 2.0 mL/min, t_R = 6.6 min (minor) / 7.6 min (minor) / 8.2 min (major) / 9.1 min (minor)	$[\alpha]_D^{27}$ = - 4 (c = 0.1, $CHCl_3$)	99, 97/3
16	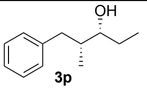 3p	GC-MS: Chiraldex β-DM column (80 iso 400 min), t_R = 108.4 min (major) / 121.8 min (minor) / 131.3 min (minor) / 133.3 min (minor)	$[\alpha]_D^{27}$ = - 3 (c = 0.1, $CHCl_3$)	99, 99/1
17	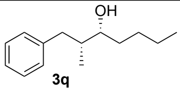 3q	SFC: Chiralpak ID column (90% CO_2 10% MeOH), 2.0 mL/min, t_R = 6.1 min (minor) / 7.3 min (major) / 7.6 min (minor) / 8.2 min (minor)	$[\alpha]_D^{27}$ = + 5 (c = 0.1, $CHCl_3$)	99, 99/1
18	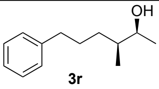 3r	SFC: Chiralpak ID column (90% CO_2 10% MeOH), 2.0 mL/min, t_R = 7.4 min (minor) / 8.5 min (minor) / 9.6 min (major) / 11.7 min (minor)	$[\alpha]_D^{27}$ = -13 (c = 0.1, $CHCl_3$)	99, 92/8

Chromatograms

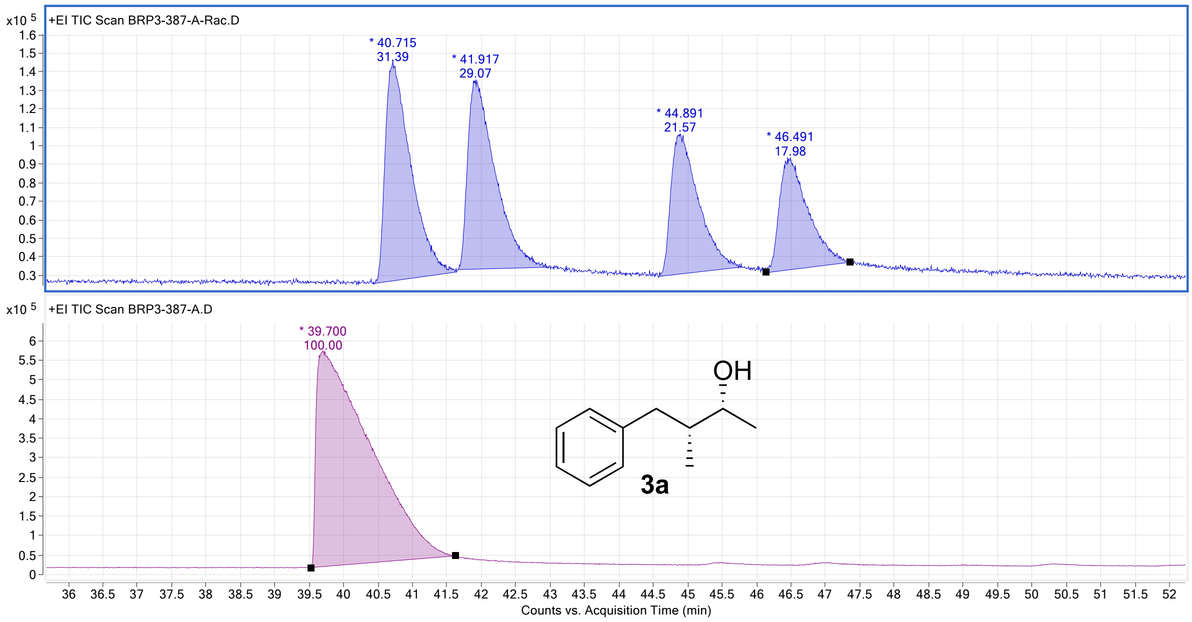

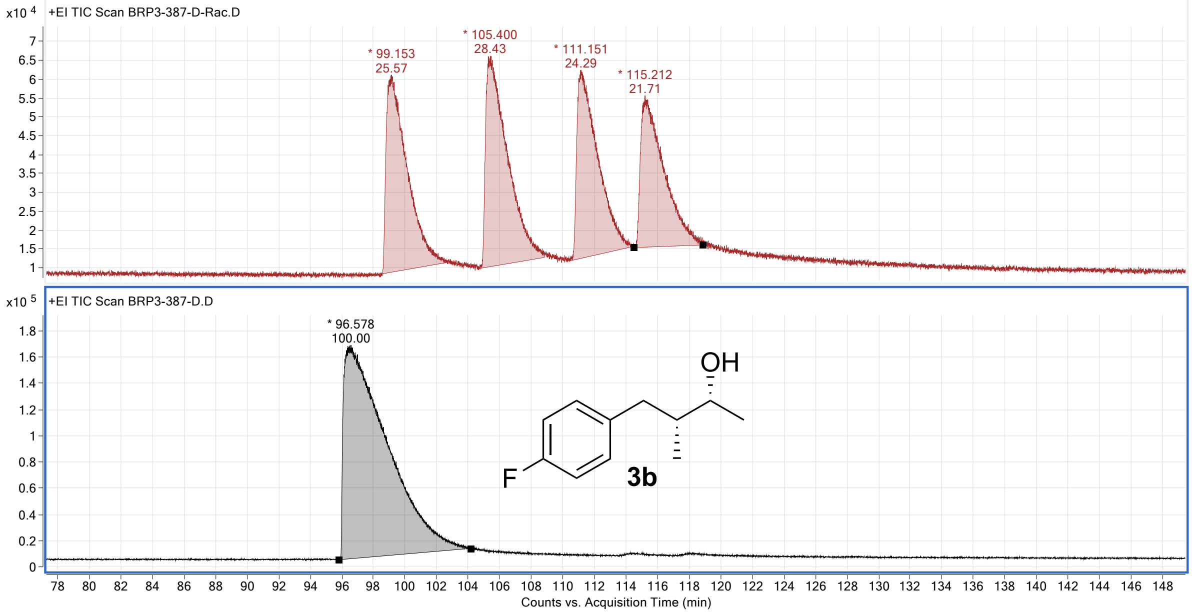

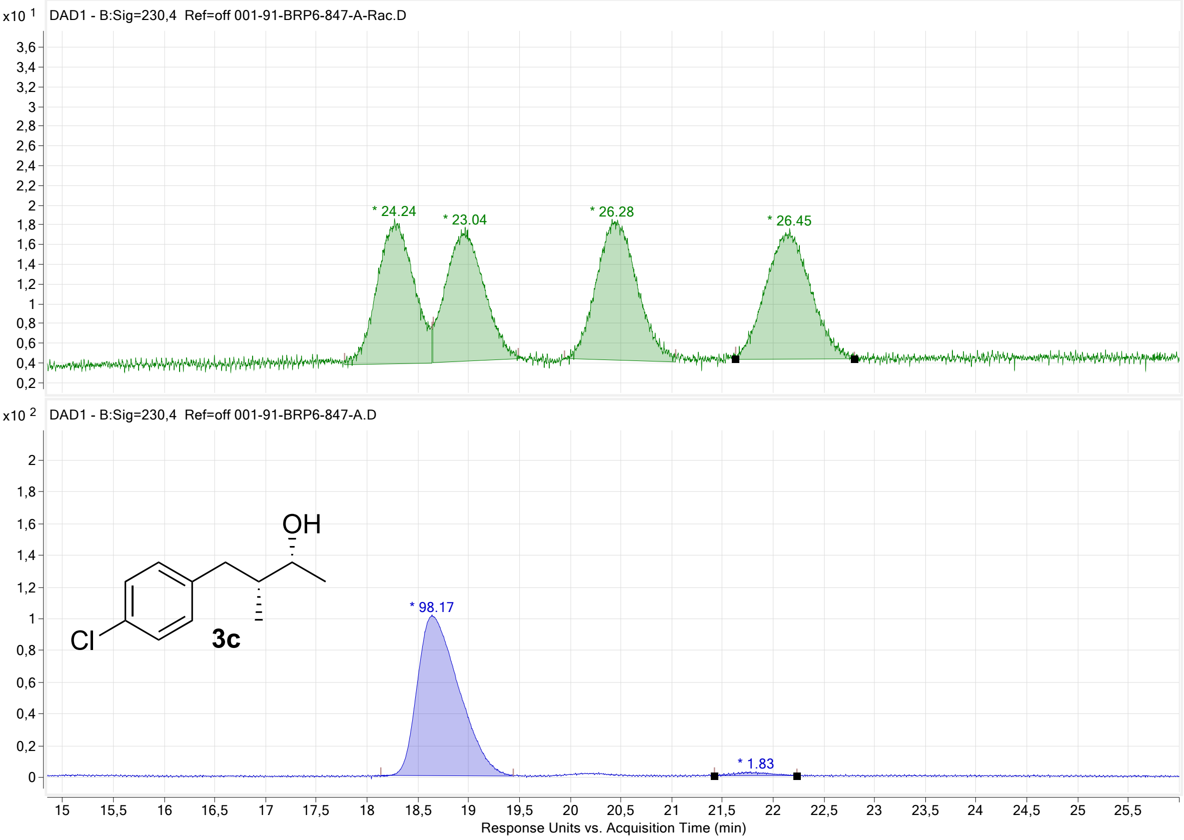
x10 1
DAD1 - B:Sig=230,4 Ref=off 001-91-BRP6-847-A-Rac.D
* 24.24
* 23.04
* 26.28
* 26.45
x10 2
DAD1 - B:Sig=230,4 Ref=off 001-91-BRP6-847-A.D
OH
Cl
3c
* 98.17
* 1.83
Response Units vs. Acquisition Time (min)

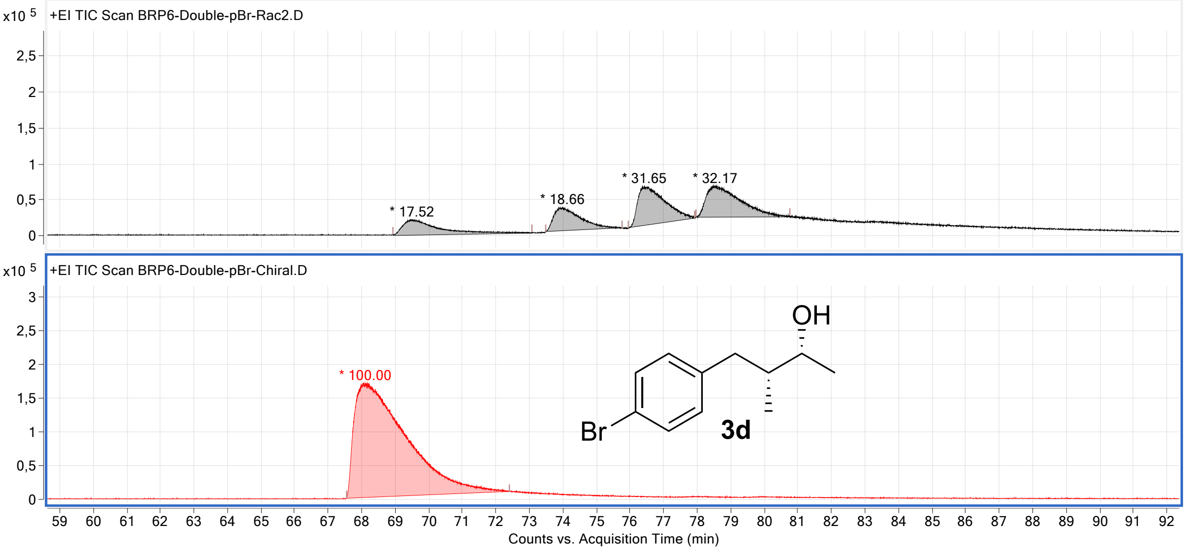
x10 5
+EI TIC Scan BRP6-Double-pBr-Rac2.D
* 17.52
* 18.66
* 31.65
* 32.17
x10 5
+EI TIC Scan BRP6-Double-pBr-Chiral.D
* 100.00
OH
Br
3d
Counts vs. Acquisition Time (min)

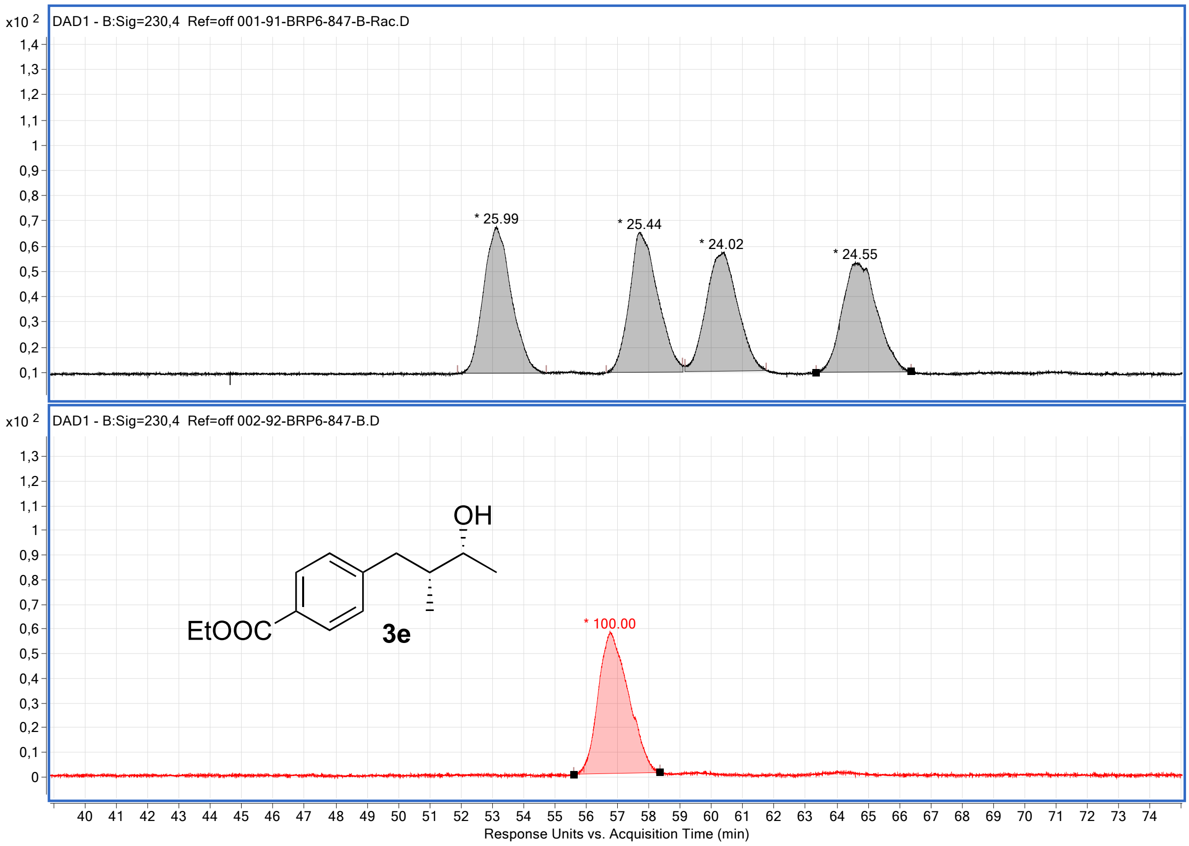

DAD1 - B:Sig=230,4 Ref=off 001-91-BRP6-847-B-Rac.D
* 25.99
* 25.44
* 24.02
* 24.55
DAD1 - B:Sig=230,4 Ref=off 002-92-BRP6-847-B.D
OH
EtOOC
3e
* 100.00
Response Units vs. Acquisition Time (min)

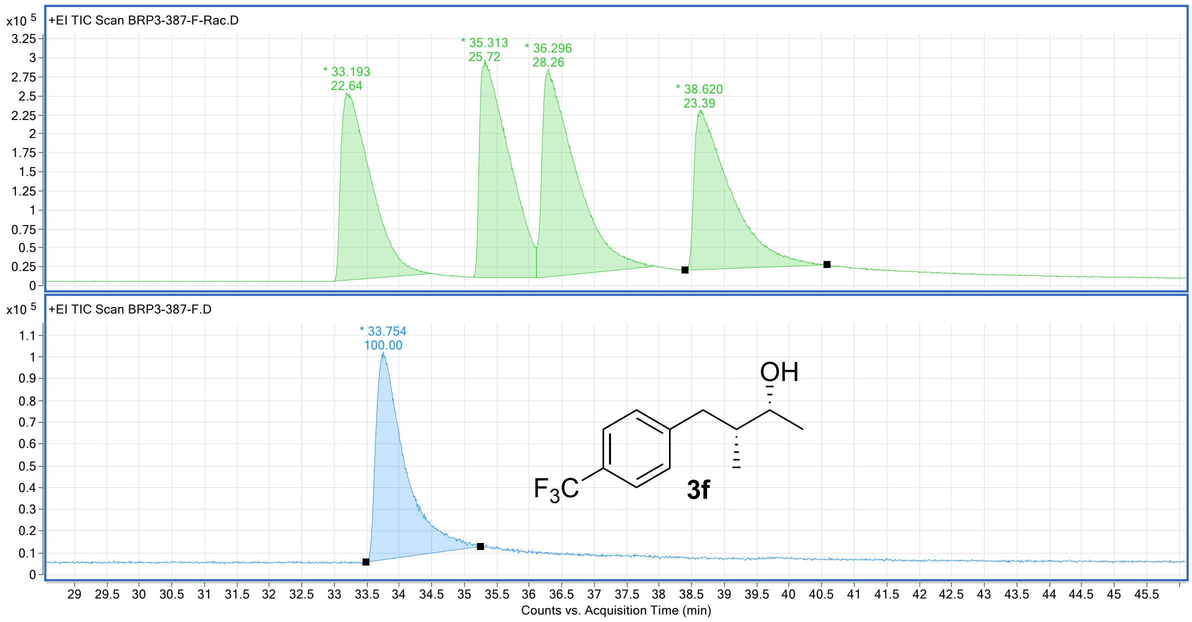

+EI TIC Scan BRP3-387-F-Rac.D
* 33.193
22.64
* 35.313
25.72
* 36.296
28.26
* 38.620
23.39
+EI TIC Scan BRP3-387-F.D
* 33.754
100.00
OH
F3C
3f
Counts vs. Acquisition Time (min)

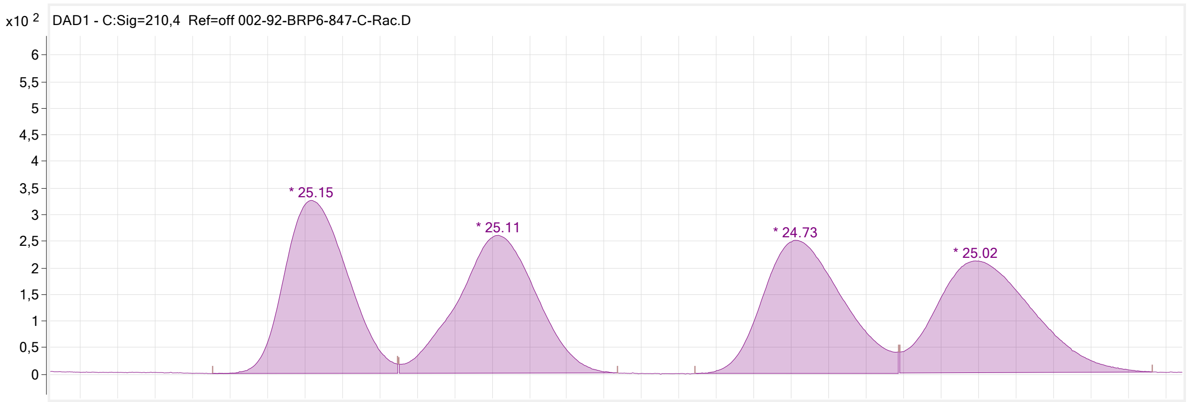
x10 2
DAD1 - C:Sig=210,4 Ref=off 002-92-BRP6-847-C-Rac.D
* 25.15
* 25.11
* 24.73
* 25.02

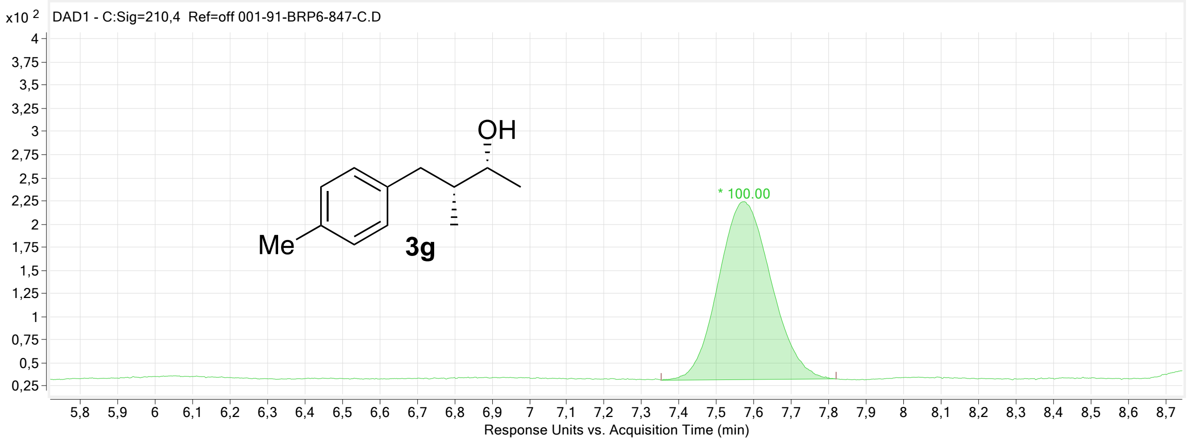
x10 2
DAD1 - C:Sig=210,4 Ref=off 001-91-BRP6-847-C.D
OH
Me
3g
* 100.00
Response Units vs. Acquisition Time (min)

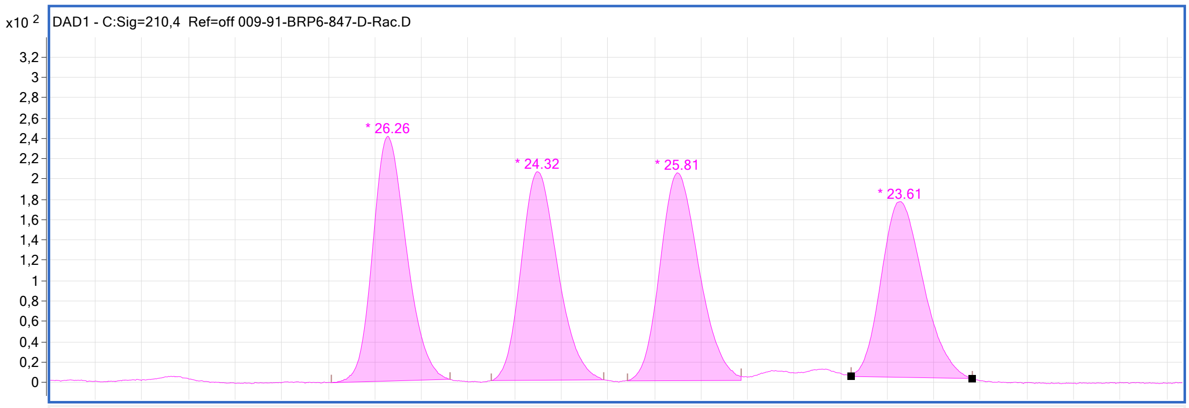
x10 2
DAD1 - C:Sig=210,4 Ref=off 009-91-BRP6-847-D-Rac.D
* 26.26
* 24.32
* 25.81
* 23.61

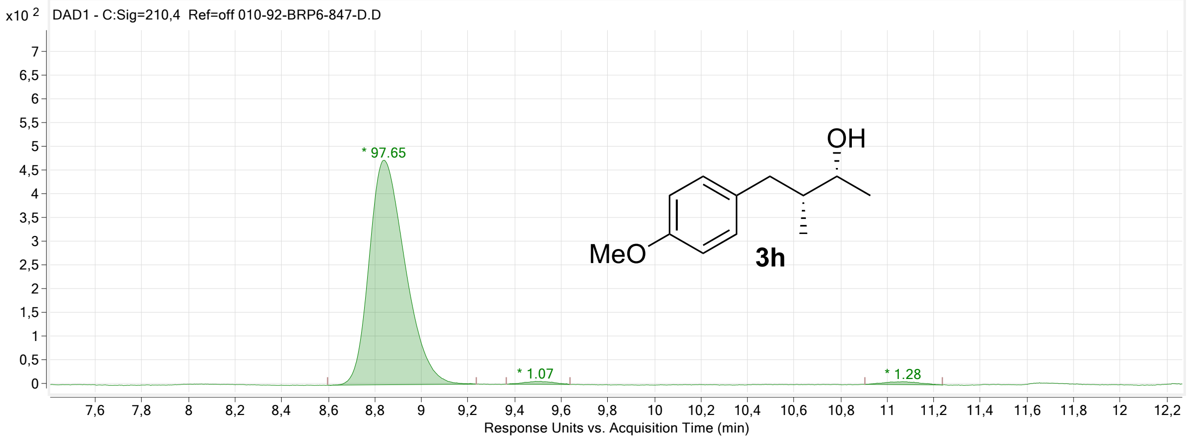
x10 2
DAD1 - C:Sig=210,4 Ref=off 010-92-BRP6-847-D.D
* 97.65
* 1.07
* 1.28
OH
MeO
3h
Response Units vs. Acquisition Time (min)

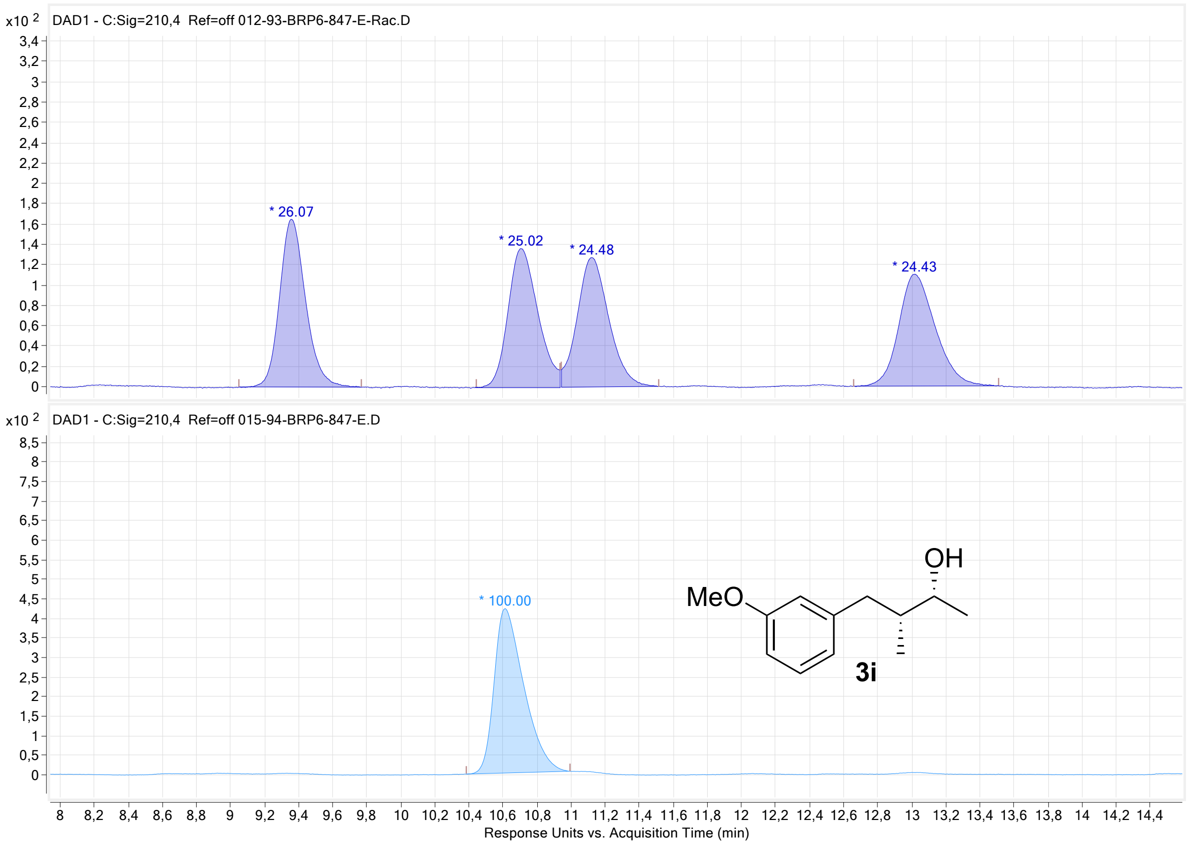
DAD1 - C:Sig=210,4 Ref=off 012-93-BRP6-847-E-Rac.D
x10 2
* 26.07
* 25.02
* 24.48
* 24.43
DAD1 - C:Sig=210,4 Ref=off 015-94-BRP6-847-E.D
x10 2
* 100.00
MeO
OH
3i
Response Units vs. Acquisition Time (min)

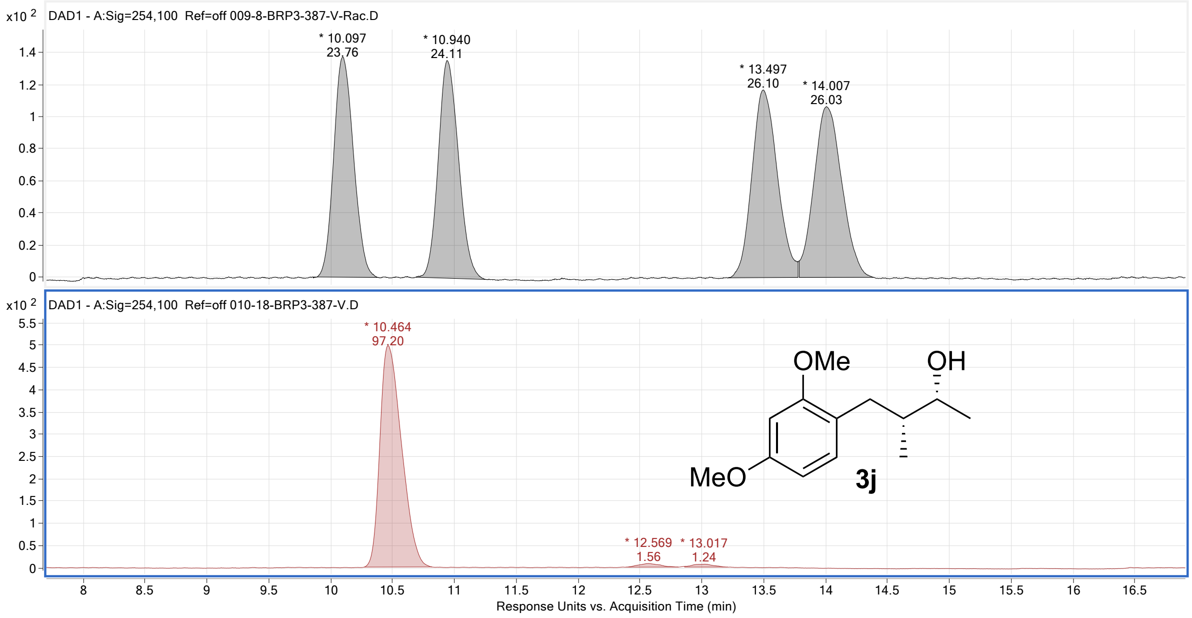
DAD1 - A:Sig=254,100 Ref=off 009-8-BRP3-387-V-Rac.D
x10 2
* 10.097
23.76
* 10.940
24.11
* 13.497
26.10
* 14.007
26.03
DAD1 - A:Sig=254,100 Ref=off 010-18-BRP3-387-V.D
x10 2
* 10.464
97.20
* 12.569
1.56
* 13.017
1.24
OMe
OH
MeO
3j
Response Units vs. Acquisition Time (min)

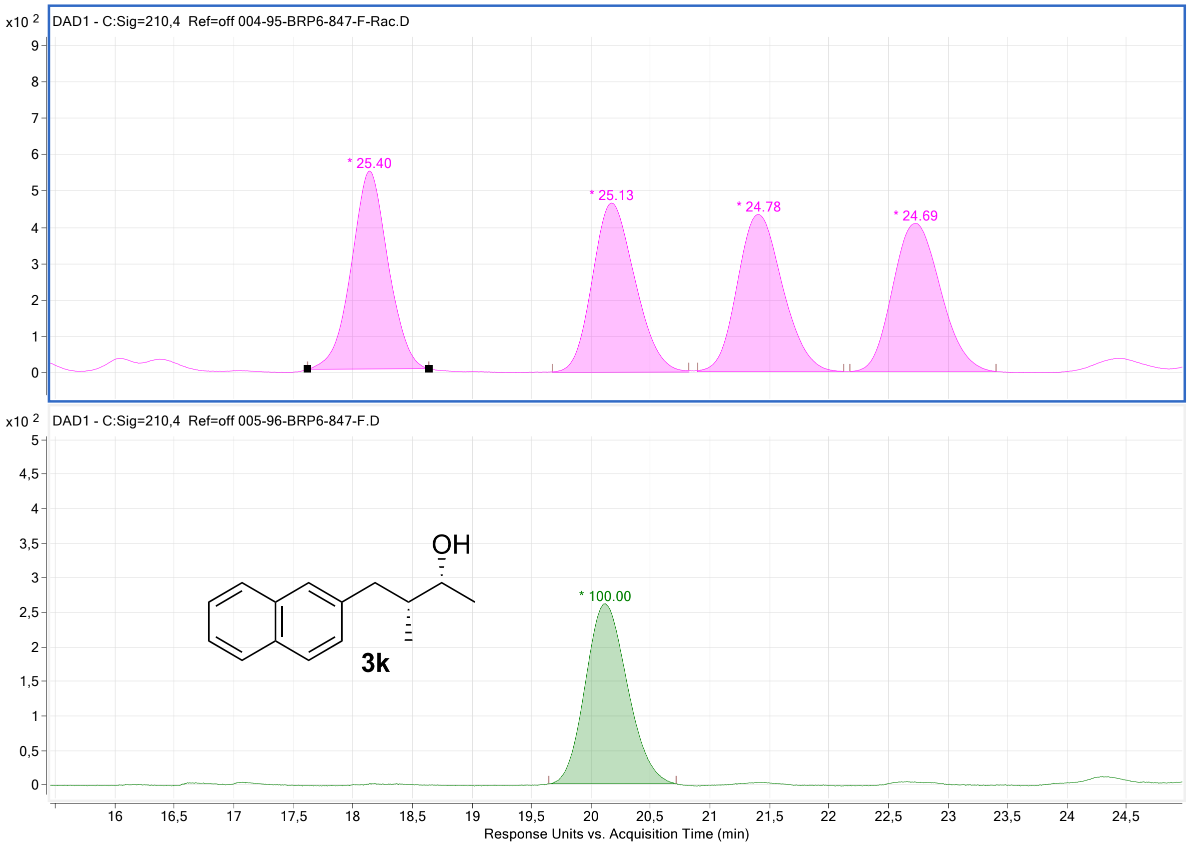
x10 2
DAD1 - C:Sig=210,4 Ref=off 004-95-BRP6-847-F-Rac.D
* 25.40
* 25.13
* 24.78
* 24.69
x10 2
DAD1 - C:Sig=210,4 Ref=off 005-96-BRP6-847-F.D
OH
3k
* 100.00
Response Units vs. Acquisition Time (min)

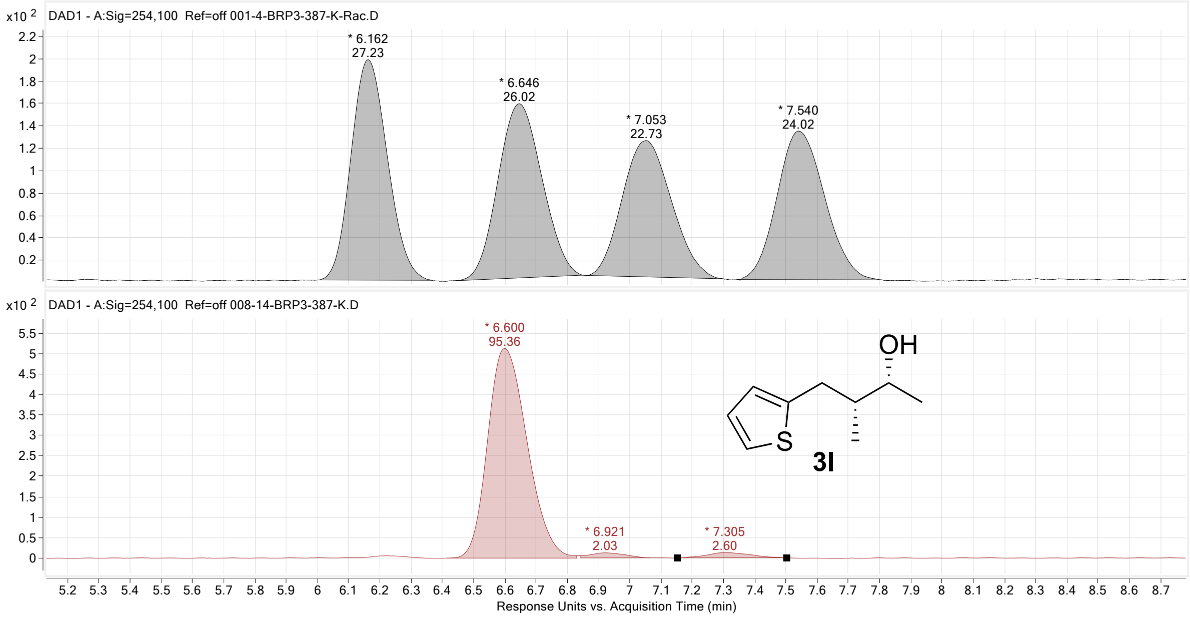
x10 2
DAD1 - A:Sig=254,100 Ref=off 001-4-BRP3-387-K-Rac.D
* 6.162
27.23
* 6.646
26.02
* 7.053
22.73
* 7.540
24.02
x10 2
DAD1 - A:Sig=254,100 Ref=off 008-14-BRP3-387-K.D
* 6.600
95.36
* 6.921
2.03
* 7.305
2.60
OH
S
3l
Response Units vs. Acquisition Time (min)

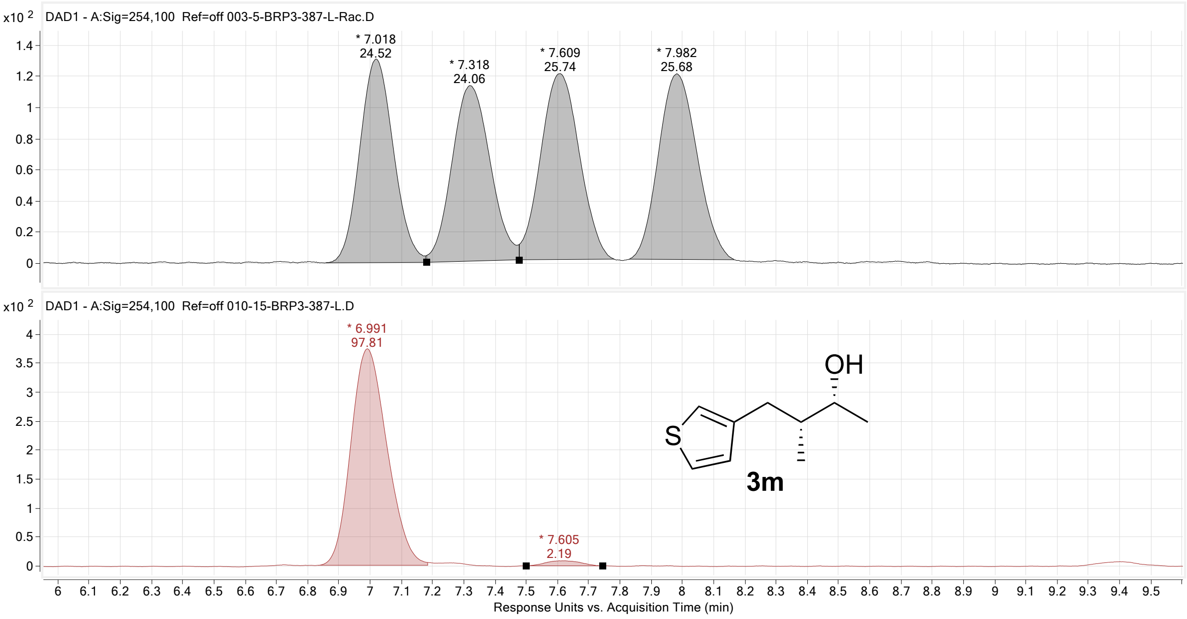
x10 2
DAD1 - A:Sig=254,100 Ref=off 003-5-BRP3-387-L-Rac.D
* 7.018
24.52
* 7.318
24.06
* 7.609
25.74
* 7.982
25.68
x10 2
DAD1 - A:Sig=254,100 Ref=off 010-15-BRP3-387-L.D
* 6.991
97.81
* 7.605
2.19
OH
S
3m
Response Units vs. Acquisition Time (min)

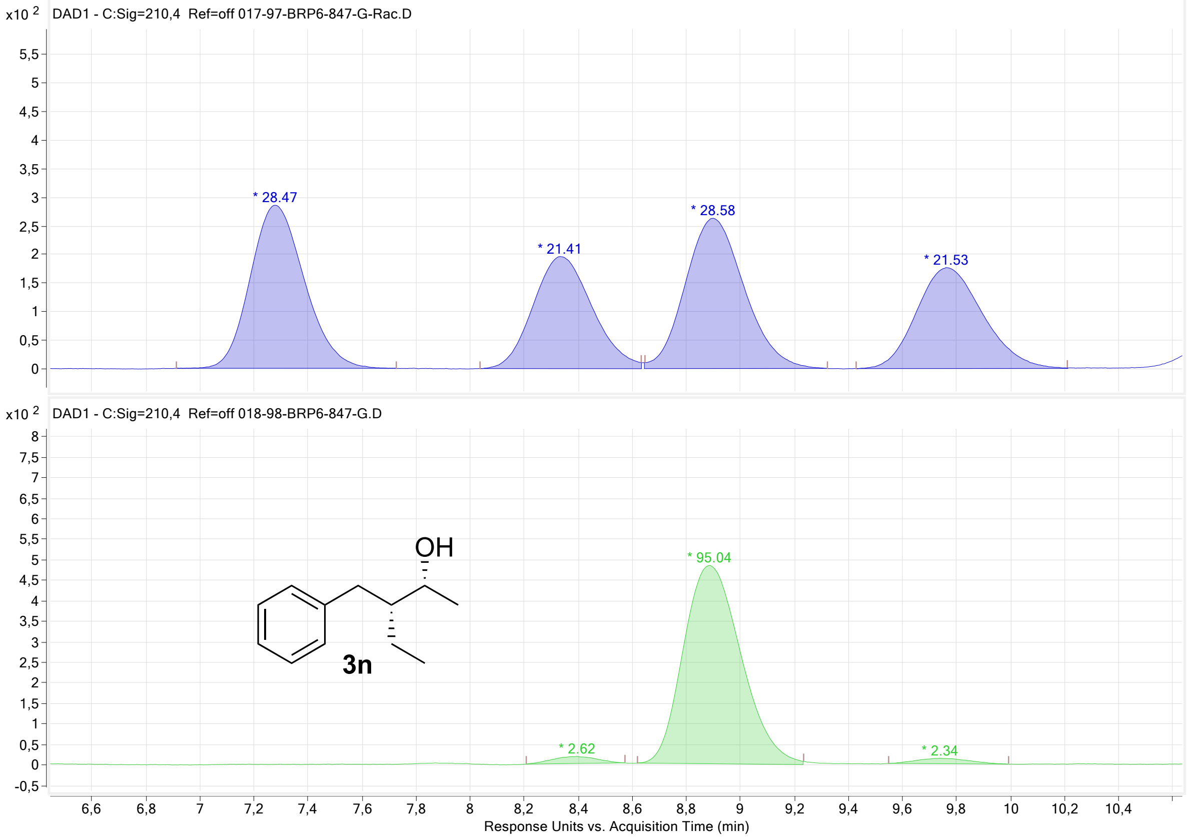
x10 2
DAD1 - C:Sig=210,4 Ref=off 017-97-BRP6-847-G-Rac.D
* 28.47
* 21.41
* 28.58
* 21.53
x10 2
DAD1 - C:Sig=210,4 Ref=off 018-98-BRP6-847-G.D
OH
3n
* 95.04
* 2.62
* 2.34
Response Units vs. Acquisition Time (min)

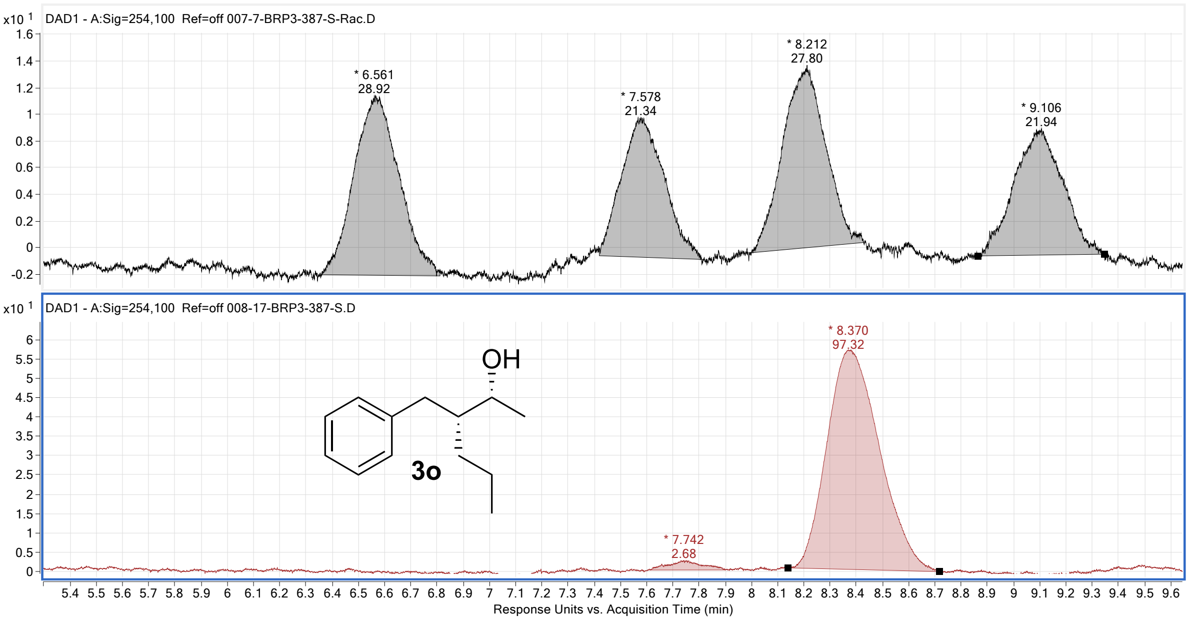
x10 1
DAD1 - A:Sig=254,100 Ref=off 007-7-BRP3-387-S-Rac.D
* 6.561
28.92
* 7.578
21.34
* 8.212
27.80
* 9.106
21.94
x10 1
DAD1 - A:Sig=254,100 Ref=off 008-17-BRP3-387-S.D
OH
3o
* 7.742
2.68
* 8.370
97.32
Response Units vs. Acquisition Time (min)

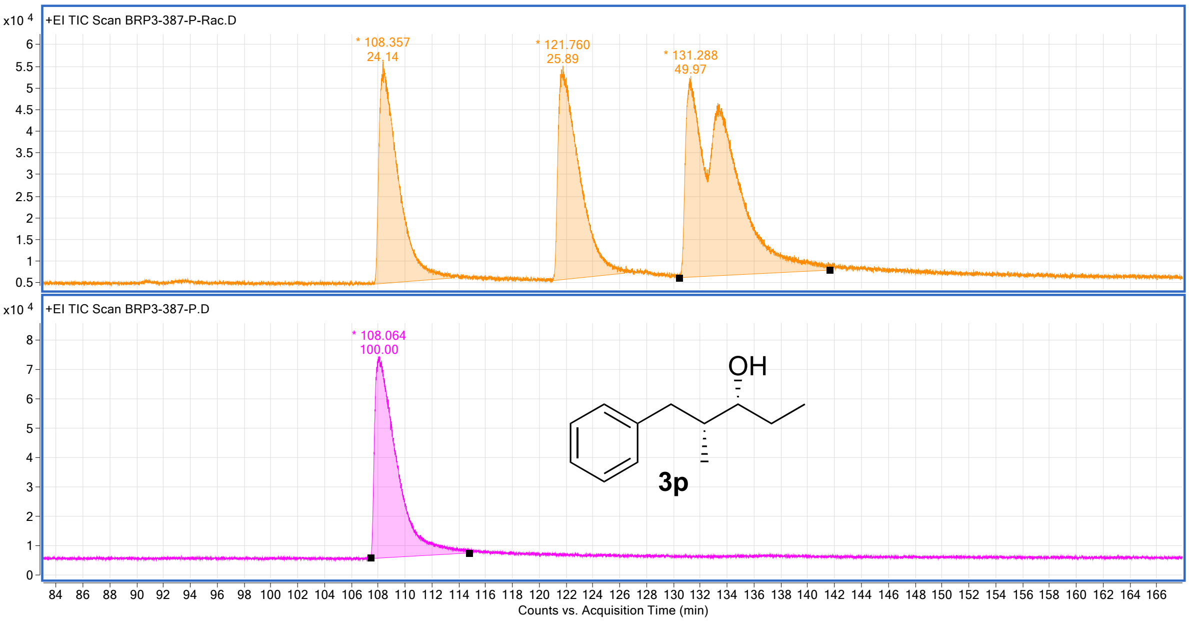
x10 4
+EI TIC Scan BRP3-387-P-Rac.D
* 108.357
24.14
* 121.760
25.89
* 131.288
49.97
x10 4
+EI TIC Scan BRP3-387-P.D
* 108.064
100.00
OH
3p
Counts vs. Acquisition Time (min)

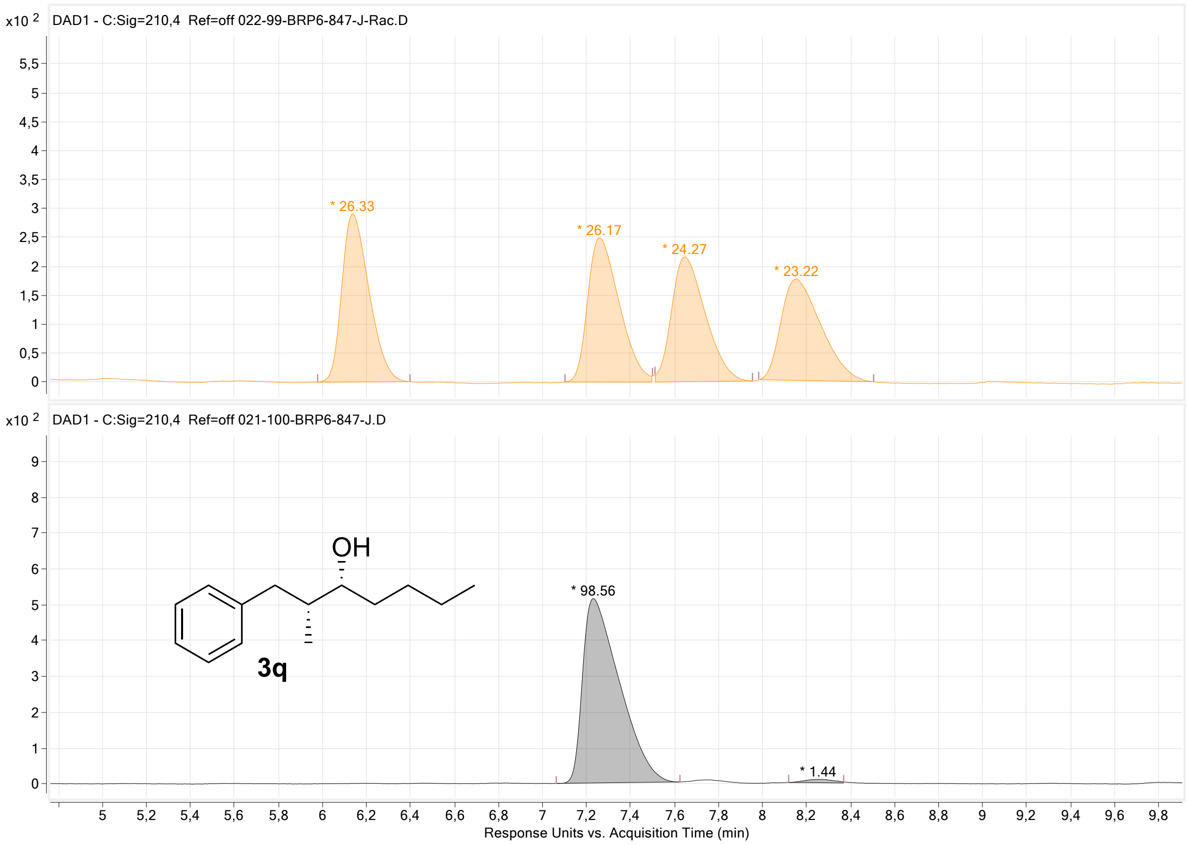
DAD1 - C:Sig=210,4 Ref=off 022-99-BRP6-847-J-Rac.D
* 26.33
* 26.17
* 24.27
* 23.22
DAD1 - C:Sig=210,4 Ref=off 021-100-BRP6-847-J.D
OH
3q
* 98.56
* 1.44
Response Units vs. Acquisition Time (min)

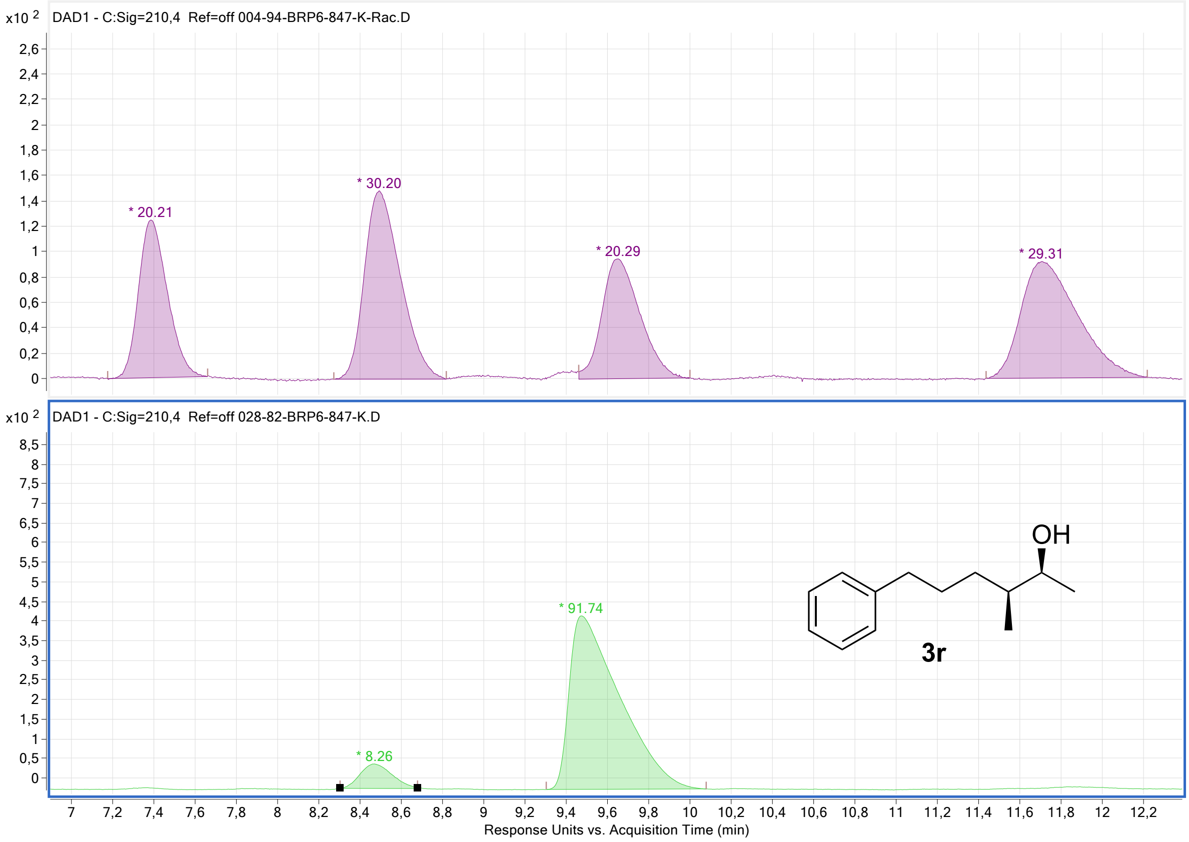
DAD1 - C:Sig=210,4 Ref=off 004-94-BRP6-847-K-Rac.D
* 20.21
* 30.20
* 20.29
* 29.31
DAD1 - C:Sig=210,4 Ref=off 028-82-BRP6-847-K.D
* 8.26
* 91.74
OH
3r
Response Units vs. Acquisition Time (min)
